# Supplementary material for: Mammalian RNA Decay Pathways Are Highly Specialized and Widely Linked to Translation
Source: Mol Cell. 2020 Mar 19;77(6):1222–1236.e13. doi: 10.1016/j.molcel.2020.01.007 (PMC7083229; doi:10.1016/j.molcel.2020.01.007)
Supplement: Document S1. Figures S1–S6 [file mmc1.pdf]

**Molecular Cell, Volume *77***

## Supplemental Information

# Mammalian RNA Decay Pathways Are Highly Specialized and Widely Linked to Translation

**Alex Charles Tuck, Aneliya Rankova, Alaaddin Bulak Arpat, Luz Angelica Liechti, Daniel Hess, Vytautas Iesmantavicius, Violeta Castelo-Szekely, David Gatfield, and Marc Bühler**

## SUPPLEMENTAL FIGURE LEGENDS

**Figure S1: Profiling mammalian RNA decay pathways (related to Figure 1). A** Western blots showing expression and biotinylation of endogenously tagged 3xFLAG-AviTag SKIV2L, XRN1 and MTR4. **B** Scree plot for the PCA in Figure 1C. **C** Western blot analysis of a co-immunoprecipitation using anti-HA to capture 2xHA-FKBP12-tagged DIS3L, and probing inputs and eluates with anti-FLAG (to detect 3xFLAG-Avi-SKIV2L) and anti-MTR4. **D** tSNE representation of mRNAs based on relative binding to MTR4, SKIV2L and XRN1, and highlighting functional classes of mRNAs. **E** RT-qPCR analysis of *Skiv2l*$^{fl/fl}$ mRNA expression levels in clones 5F and 9E (Table S1) after 0.1 µM 4OHT treatment for 0, 2, 4 and 6 days. Error bars denote standard deviation of two technical replicates. Values are normalized to TBP expression and then to untreated samples (day 0). **F** Boxplot for the 7240 mRNAs analyzed in Figure 1I, grouped by differential expression after four days of *Skiv2l* knockout, and showing the extent to which they bind SKIV2L in WT cells (relative binding based on CRAC, defined in Figure 1E). Box widths are proportional to the number of contained transcripts. $p < 10^{-15}$ for upregulated versus downregulated transcripts (Mann-Whitney U test). **G** $Log_2$-fold changes in transcriptome-wide mRNA half-lives for *Skiv2l*$^{fl/fl}$ cells following 0.1 µM 4OHT treatment for 4 or 0 days. Half-lives were calculated by fitting an exponential decay model to RNA-seq counts from an actinomycin D-mediated transcription shut-off time course. The x-axis shows the extent of SKIV2L binding as a fraction of total SKIV2L+XRN1 binding (i.e. SKIV2L CRAC divided by SKIV2L+XRN1 CRAC, using “relative binding” values defined in Figure 1E). High confidence SKIV2L targets are indicated in red, and are defined as mRNAs with an increased half-life after *Skiv2l* knockout (y-axis > 0.1), high SKIV2L binding (x-axis > 0.5) and significant RNA increase after 4 days of *Skiv2l* knockout (Figure 1I). The half-lives of the 200 most highly SKIV2L-bound mRNAs (right of green dashed line) were compared to those of all other mRNAs using a Student’s t-test. **H** RNA levels for high confidence SKIV2L targets (red circles in G) for wild-type, tagged (*Skiv2l*$^{3xFLAG\text{-}Avi/3xFLAG\text{-}Avi}$, *Mtr4*$^{3xFLAG\text{-}Avi/3xFLAG\text{-}Avi}$ and *Xrn1*$^{3xFLAG\text{-}Avi/3xFLAG\text{-}Avi}$) and *Skiv2l*$^{fl/fl}$ (+ 4 days 4OHT) cells. Each point refers to a separate cell line. See also Tables S1-S3.

**Figure S2: Defining triggers of RNA decay (related to Figure 2). A** Proportion of CRAC mRNA reads with non-templated homopolymeric 3' tails, for XRN1, SKIV2L and MTR4. **B** SKIV2L specific enrichment at amino acid pairs, compared to a panel of control datasets. For each amino acid pair, the proportion of hexamers encoding that amino acid pair and bound by SKIV2L is shown. This calculation was performed for in-frame hexamers (y-axis) and out-of-frame hexamers (x-axis). **C** Disome profiling (A sites) and SKIV2L and XRN1 CRAC coverage (3' end of RNA fragments) around runs of 3-4 identical amino acids (e.g. KKK/KKKK), normalized to downstream signal. **D** Pileups around polypurine tracts for monosome and disome profiling and SKIV2L, MTR4 and XRN1 CRAC signal. Polypurine tracts are divided by lysine content (top) or A versus G content ($\geq$ 50 % A, or > 50 % G).

**Figure S3: AVEN modulates SKIV2L binding (related to Figure 4). A** Scree plot for the PCA in Figure 4C. **B** Left, schematic of CRISPR-Cas9-induced *Aven* knockout and RNA-seq tracks in the parental *Skiv2l*$^{3xFLAG\text{-}Avi/3xFLAG\text{-}Avi}$ and *Aven*$^{-/-}$ clones 4H and 6G. Right, western blot analysis of AVEN expression. **C** RT-qPCR analysis of *Focad* mRNA levels in *Focad*$^{-/-}$ clones 2F and 4B and WT cells. Error bars denote standard deviation of four technical replicates. **D and E** Boxplot representation of the data shown in Figure 4F, including all transcripts (not only those differentially bound or expressed). AVEN CRAC counts in WT are shown (y-axis) for transcripts grouped by changes in SKIV2L binding (B; $p < 10^{-15}$) or RNA abundance (C; p = 0.002) in *Aven*$^{-/-}$ versus WT (x-axis). P-values calculated using a Mann-Whitney U test to compare up- and down-regulated transcripts. See also Tables S1 and S3.

**Figure S4: AVEN and SKIV2L counteract ribosome stalling (related to Figures 4 and 5). A** Changes in mRNA monosome and disome densities in *Aven*$^{-/-}$ versus WT. Transcripts are colored by AVEN binding in WT cells (calculated as in Figure 4F; high, n = 777; low, n = 5471), and a linear best fit line plotted for each group (shaded area = 95 % confidence interval). **B** Western blot analysis of SKIV2L levels in *Aven*$^{-/-}$

*Skiv2l*$^{3xFLAG\text{-}Avi/3xFLAG\text{-}Avi}$ *Skiv2l*$^{fl/fl}$ cells after treatment with 0.1 µM 4OHT for 0, 2 or 4 days to induce *Skiv2l* knockout. **C** $Log_2$ fold change in the half-lives of AVEN targets, which are defined as mRNAs that accumulate upon combined knockout of *Aven* and *Skiv2l* ($p < 0.01$; Figure 5D) and that have increased SKIV2L binding in *Aven*$^{-/-}$ cells (differential binding > 0.5, Figure 5D). Three comparisons are shown (left to right): (i) *Skiv2l*$^{fl/fl}$ 0 vs 4 days 4OHT (i.e. *Skiv2l* knockout vs wild-type), (ii) *Skiv2l*$^{fl/fl}$ vs *Aven*$^{-/-}$ *Skiv2l*$^{3xFLAG\text{-}Avi/3xFLAG\text{-}Avi}$ *Skiv2l*$^{fl/fl}$ with no 4OHT treatment (i.e. *Aven* knockout vs wild-type), and (iii) *Aven*$^{-/-}$ *Skiv2l*$^{3xFLAG\text{-}Avi/3xFLAG\text{-}Avi}$ *Skiv2l*$^{fl/fl}$ 0 vs 4 days 4OHT (i.e. *Skiv2l Aven* double knockout versus *Aven* knockout). Half-lives are calculated from RNA-seq decay curves following actinomycin D-mediated transcription shut-off. Replication-dependent histone mRNAs are colored red. For each of the three comparisons, a Student's t-test was used to test whether the distribution of half-life $log_2$-fold changes for the set of mRNAs shown here (AVEN targets) differs from that of all other mRNAs. **D** Top, scheme depicting mESC cell cycle synchronization at the G1/S boundary using a double-thymidine block. Bottom, flow cytometry analysis of DAPI-stained asynchronous or thymidine-synchronized cells at 0, 4 and 8 hours after release from the second thymidine block for the indicated cell lines. **E** Boxplot showing the change in uORF monosome profiling counts (normalized to main CDS counts) for *Aven*$^{-/-}$ versus WT cells. uORFs are categorized (x-axis) by their AVEN CRAC counts in WT cells (n = 558, 215 and 53 for low, medium and high categories; Mann-Whitney U test $p = 3.19 \times 10^{-6}$ comparing high and low categories). **E** Western blot analysis of ATF4 and eIF2α-phospho-Serine51 levels in WT and *Aven*$^{-/-}$ cells after 0, 0.5, 2 and 4 hours of treatment with 200 nM thapsigargin. See also Tables S3 and S4.

**Figure S5: Defining sites dependent on AVEN and SKIV2L (related to Figure 6). A** $Log_2$ fold changes in SKIV2L CRAC counts for 1 kb windows tiling the genome, in *Aven*$^{-/-}$ versus WT cells. Points are colored by the proportion of U-tailed reads within each window in WT (left) or *Aven*$^{-/-}$ (right) cells. **B** SKIV2L CRAC, disome and monosome profiling reads piled up around predicted structured regions (minimum free

energy <-12 kcal/mol and continuous stretch of paired nucleotides ≥10), normalized to the downstream region. For SKIV2L CRAC in *Aven*$^{-/-}$, U-tailed reads are also shown.

**Figure S6: Features of translated non-coding RNAs (related to Figure 7). A** GC content and **B** predicted free energy for the "non-coding" 1 kb genomic windows analyzed in Figure 7C. Windows are split into four categories based on differential SKIV2L CRAC counts in *Aven*$^{-/-}$ versus WT cells (defined in Figure 7C). **C** PhyloCSF scores for predicted small ORFs within these windows. Windows are classified based upon whether SKIV2L CRAC and monosome profiling counts increase in *Aven*$^{-/-}$ versus WT cells, or not. See also Tables S6 and S7.

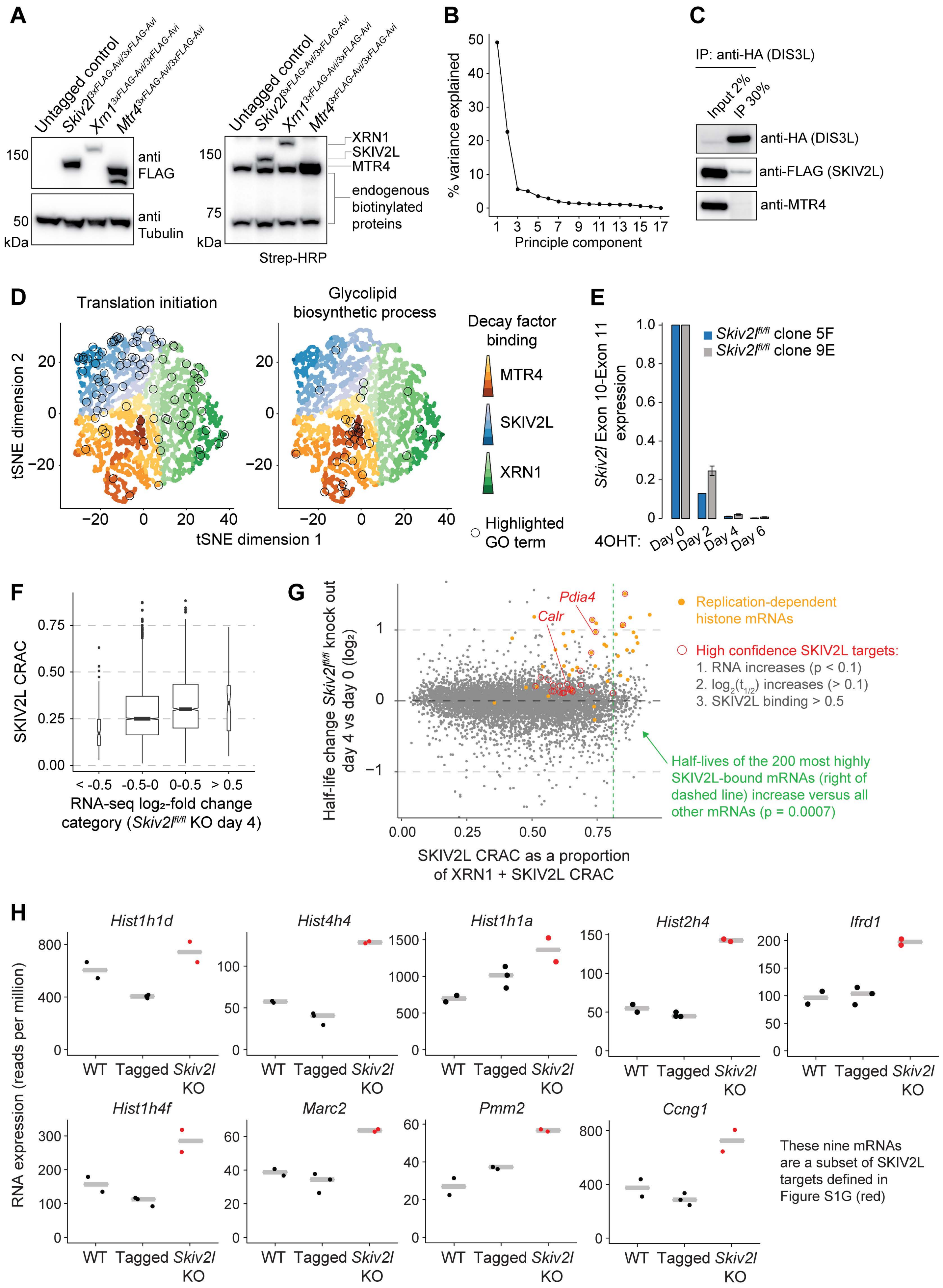

A
Untagged control
Skiv2l3xFLAG-Avi/3xFLAG-Avi
Xrn13xFLAG-Avi/3xFLAG-Avi
Mtr43xFLAG-Avi/3xFLAG-Avi
150
50
kDa
anti FLAG
anti Tubulin
75
XRN1
SKIV2L
MTR4
endogenous biotinylated proteins
Strep-HRP
B
% variance explained
Principle component
C
IP: anti-HA (DIS3L)
Input 2%
IP 30%
anti-HA (DIS3L)
anti-FLAG (SKIV2L)
anti-MTR4
D
Translation initiation
Glycolipid biosynthetic process
tSNE dimension 1
tSNE dimension 2
Decay factor binding
MTR4
SKIV2L
XRN1
Highlighted GO term
E
Skiv2l Exon 10-Exon 11 expression
Skiv2lfl/fl clone 5F
Skiv2lfl/fl clone 9E
4OHT:
Day 0
Day 2
Day 4
Day 6
F
SKIV2L CRAC
< -0.5
-0.5-0
0-0.5
> 0.5
RNA-seq log₂-fold change category (Skiv2lfl/fl KO day 4)
G
Half-life change Skiv2lfl/fl knock out day 4 vs day 0 (log₂)
SKIV2L CRAC as a proportion of XRN1 + SKIV2L CRAC
Pdia4
Calr
Replication-dependent histone mRNAs
High confidence SKIV2L targets:
1. RNA increases (p < 0.1)
2. log2(t1/2) increases (> 0.1)
3. SKIV2L binding > 0.5
Half-lives of the 200 most highly SKIV2L-bound mRNAs (right of dashed line) increase versus all other mRNAs (p = 0.0007)
H
RNA expression (reads per million)
Hist1h1d
Hist4h4
Hist1h1a
Hist2h4
Ifrd1
Hist1h4f
Marc2
Pmm2
Ccng1
WT
Tagged
Skiv2l KO
These nine mRNAs are a subset of SKIV2L targets defined in Figure S1G (red)


Figure S1

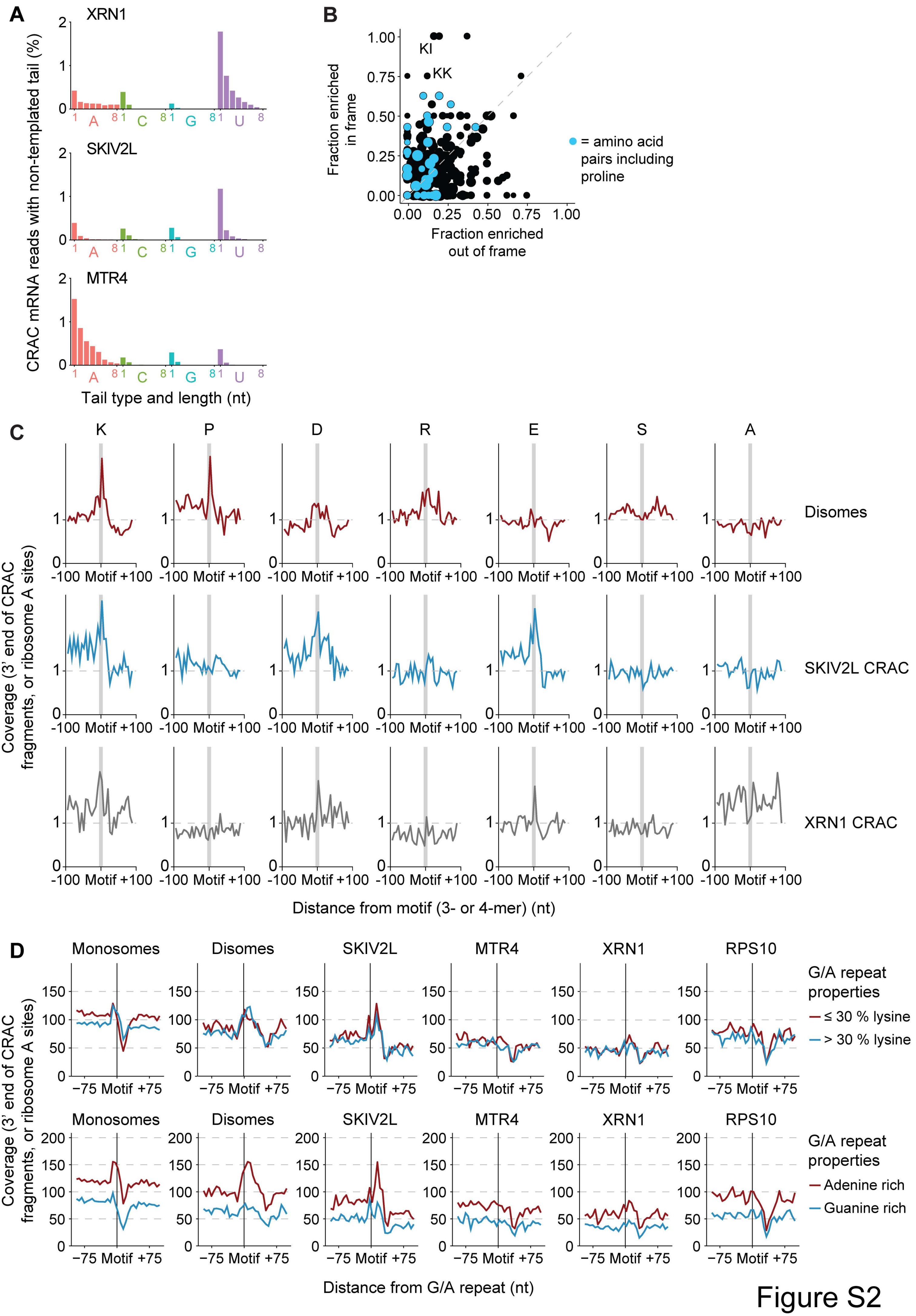

A
XRN1
SKIV2L
MTR4
CRAC mRNA reads with non-templated tail (%)
Tail type and length (nt)
B
KI
KK
Fraction enriched in frame
Fraction enriched out of frame
= amino acid pairs including proline
C
K
P
D
R
E
S
A
Disomes
SKIV2L CRAC
XRN1 CRAC
Coverage (3' end of CRAC fragments, or ribosome A sites)
-100 Motif +100
Distance from motif (3- or 4-mer) (nt)
D
Monosomes
Disomes
SKIV2L
MTR4
XRN1
RPS10
G/A repeat properties
≤ 30 % lysine
> 30 % lysine
Adenine rich
Guanine rich
−75 Motif +75
Distance from G/A repeat (nt)


Figure S2

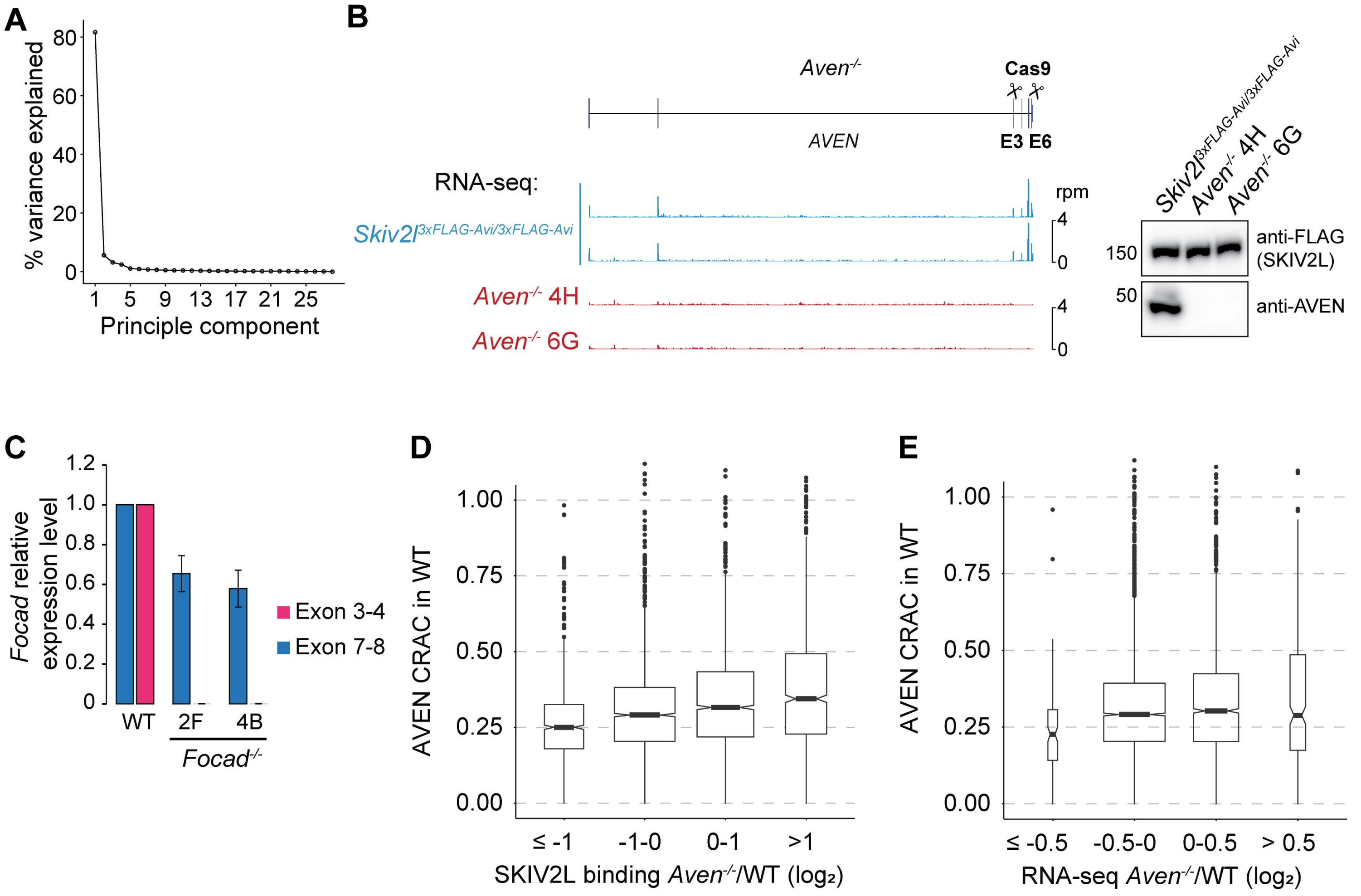

A
% variance explained
Principle component
B
Aven-/-
Cas9
AVEN
E3 E6
RNA-seq:
Skiv2l3xFLAG-Avi/3xFLAG-Avi
Aven-/- 4H
Aven-/- 6G
rpm
Skiv2l3xFLAG-Avi/3xFLAG-Avi
Aven-/- 4H
Aven-/- 6G
150
50
anti-FLAG
(SKIV2L)
anti-AVEN
C
Focad relative
expression level
Exon 3-4
Exon 7-8
WT
2F
4B
Focad-/-
D
AVEN CRAC in WT
≤ -1
-1-0
0-1
>1
SKIV2L binding Aven-/-/WT (log2)
E
AVEN CRAC in WT
≤ -0.5
-0.5-0
0-0.5
> 0.5
RNA-seq Aven-/-/WT (log2)


Figure S3

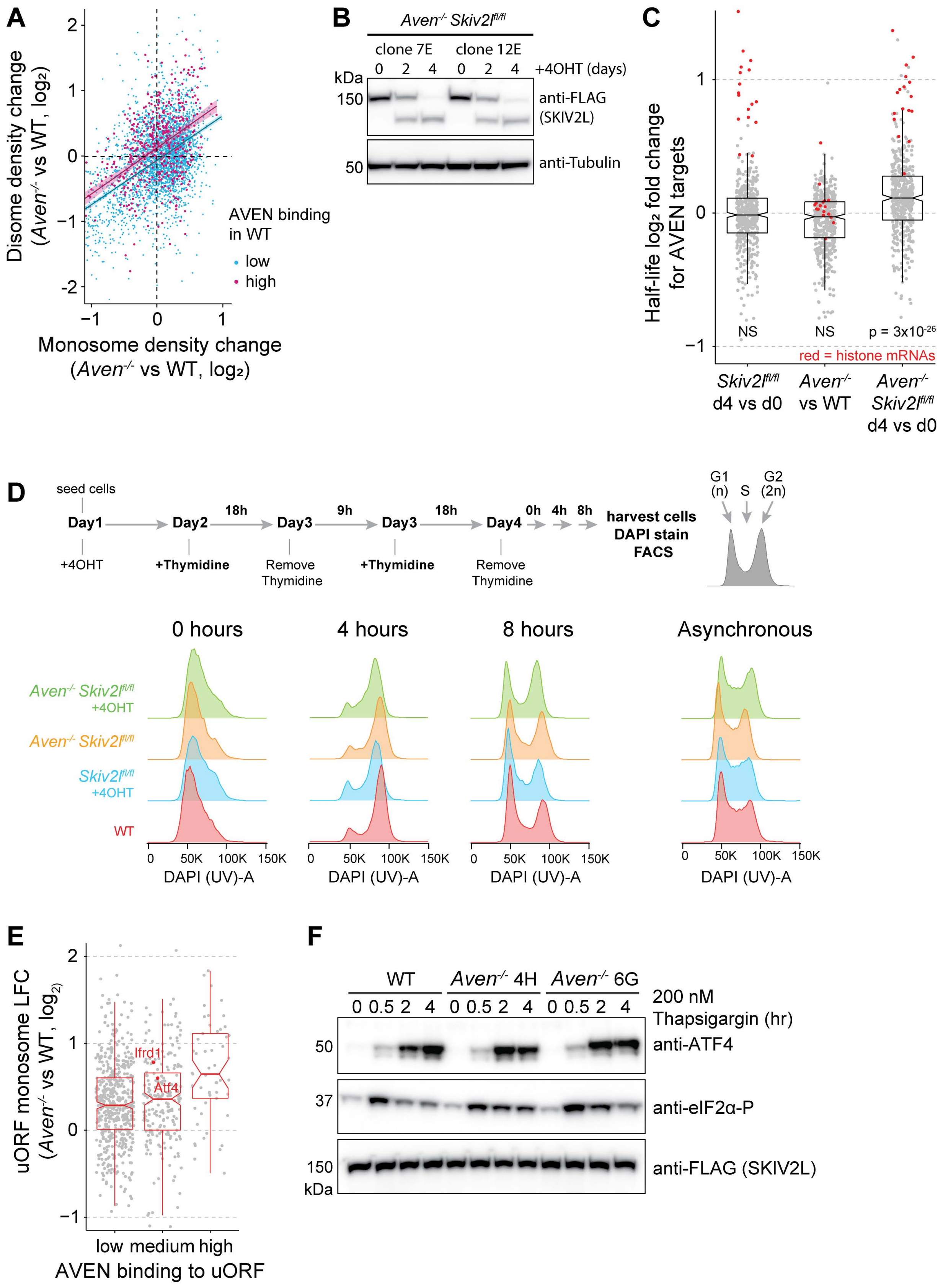

A
Disome density change (Aven-/- vs WT, log2)
Monosome density change (Aven-/- vs WT, log2)
AVEN binding in WT
low
high
B
Aven-/- Skiv2lfl/fl
clone 7E
clone 12E
kDa
0 2 4 0 2 4 +4OHT (days)
150
50
anti-FLAG (SKIV2L)
anti-Tubulin
C
Half-life log2 fold change for AVEN targets
NS
NS
p = 3x10-26
red = histone mRNAs
Skiv2lfl/fl d4 vs d0
Aven-/- vs WT
Aven-/- Skiv2lfl/fl d4 vs d0
D
seed cells
Day1
+4OHT
Day2
+Thymidine
18h
Day3
Remove Thymidine
9h
Day3
+Thymidine
18h
Day4
Remove Thymidine
0h 4h 8h
harvest cells
DAPI stain
FACS
G1 (n)
S
G2 (2n)
0 hours
4 hours
8 hours
Asynchronous
Aven-/- Skiv2lfl/fl +4OHT
Aven-/- Skiv2lfl/fl
Skiv2lfl/fl +4OHT
WT
DAPI (UV)-A
E
uORF monosome LFC (Aven-/- vs WT, log2)
Ifrd1
Atf4
low medium high
AVEN binding to uORF
F
WT
Aven-/- 4H
Aven-/- 6G
0 0.5 2 4 0 0.5 2 4 0 0.5 2 4
200 nM Thapsigargin (hr)
anti-ATF4
anti-eIF2α-P
anti-FLAG (SKIV2L)
kDa


Figure S4

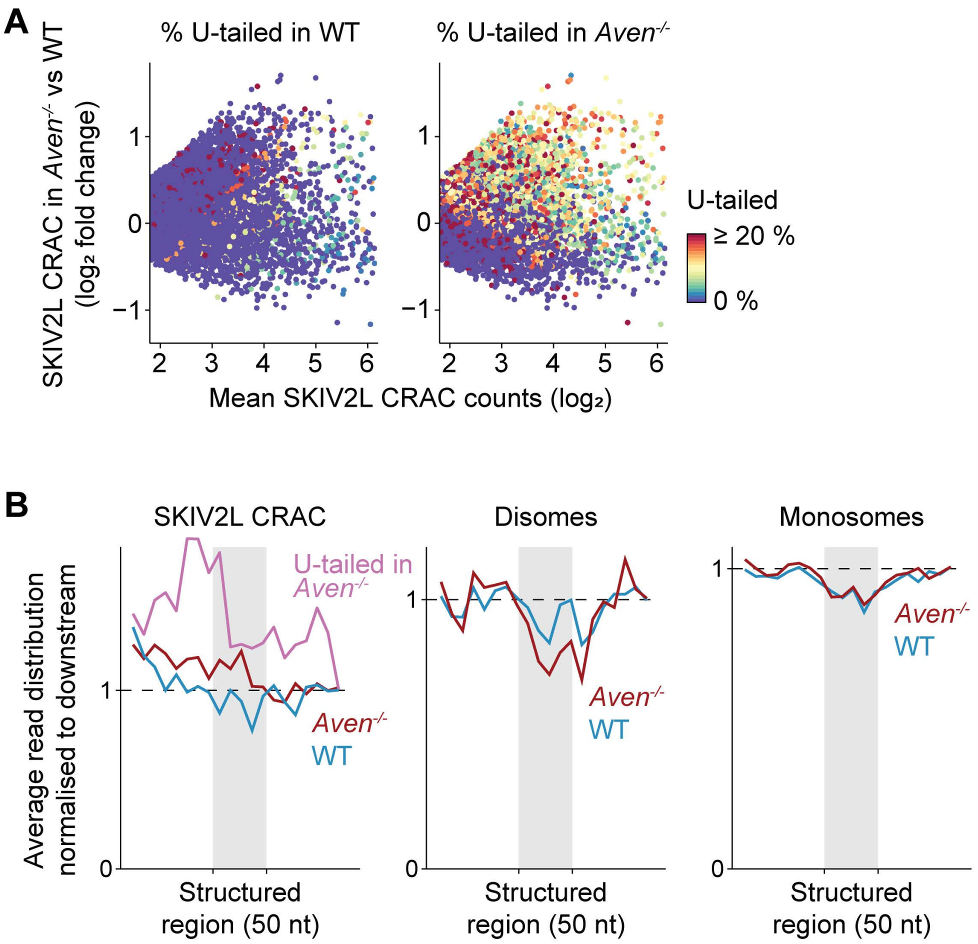
A
% U-tailed in WT
% U-tailed in Aven-/-
SKIV2L CRAC in Aven-/- vs WT
(log₂ fold change)
1
0
−1
2 3 4 5 6
Mean SKIV2L CRAC counts (log₂)
U-tailed
≥ 20 %
0 %
B
SKIV2L CRAC
Disomes
Monosomes
Average read distribution
normalised to downstream
U-tailed in
Aven-/-
Aven-/-
WT
Structured
region (50 nt)

Figure S5

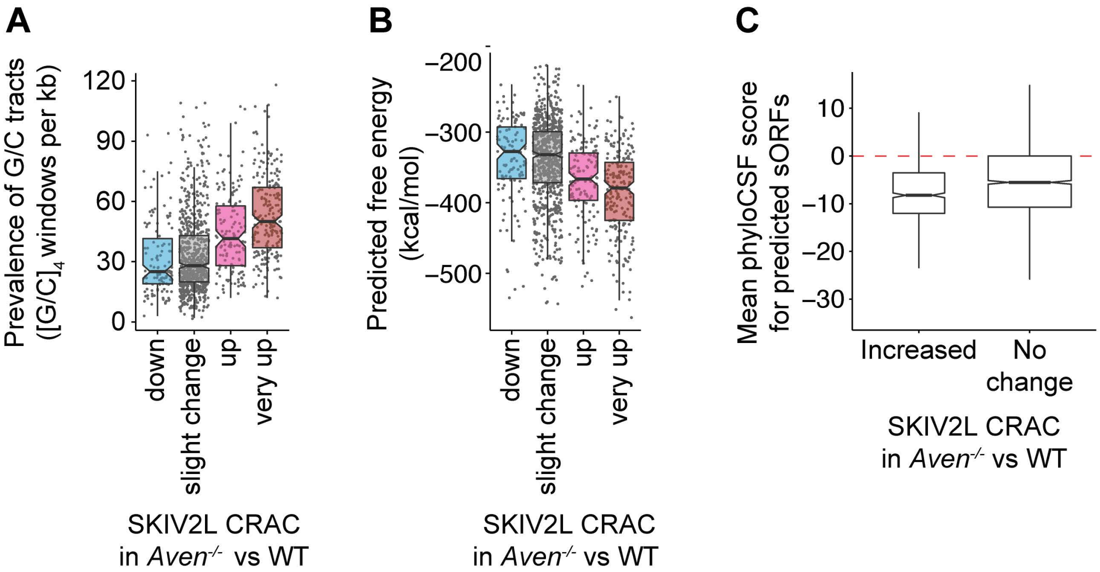
A
Prevalence of G/C tracts
([G/C]4 windows per kb)
120
90
60
30
0
down
slight change
up
very up
SKIV2L CRAC
in Aven-/- vs WT
B
Predicted free energy
(kcal/mol)
−200
−300
−400
−500
down
slight change
up
very up
SKIV2L CRAC
in Aven-/- vs WT
C
Mean phyloCSF score
for predicted sORFs
10
0
−10
−20
−30
Increased
No
change
SKIV2L CRAC
in Aven-/- vs WT

Figure S6
