# Supplementary material for: Mammalian RNA Decay Pathways Are Highly Specialized and Widely Linked to Translation
Source: Mol Cell. 2020 Mar 19;77(6):1222–1236.e13. doi: 10.1016/j.molcel.2020.01.007 (PMC7083229; doi:10.1016/j.molcel.2020.01.007)
Supplement: Document S2. Article plus Supplemental Information [file mmc9.pdf]

Article

Molecular Cell

Mammalian RNA Decay Pathways Are Highly Specialized and Widely Linked to Translation

Graphical Abstract

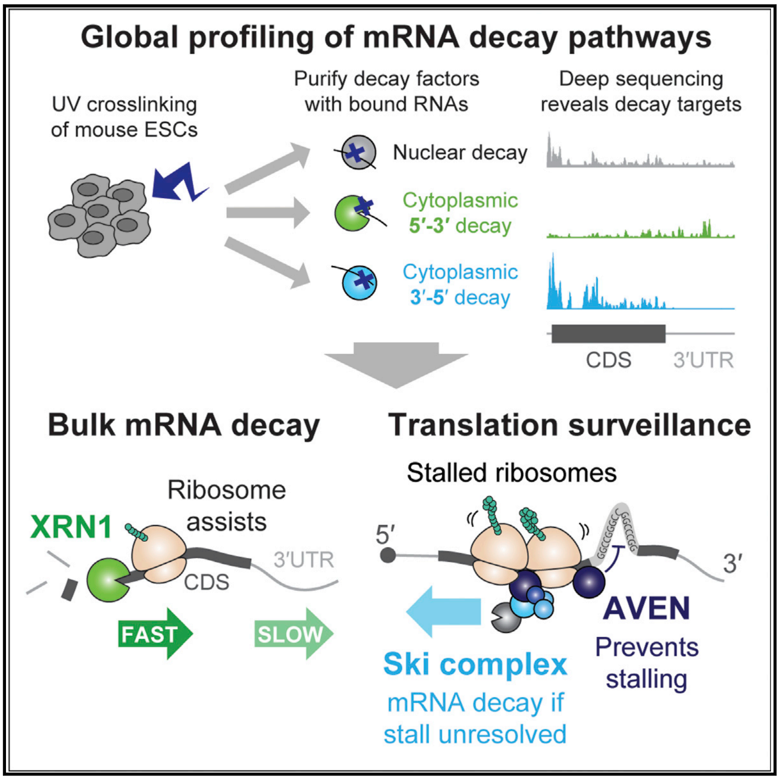

Authors

Alex Charles Tuck, Aneliya Rankova, Alaaddin Bulak Arpat, ..., Violeta Castelo-Szekely, David Gatfield, Marc Bühler

Correspondence

marc.buehler@fmi.ch

In Brief

Tuck, Rankova, et al. globally profile mRNA decay and aberrant translation events in mouse embryonic stem cells, finding that mRNA decay pathways perform specialized roles rather than acting redundantly. They uncover widespread crosstalk between mRNA decay and translation and identify AVEN as a factor that counteracts ribosome stalling.

Highlights

- Global profiling of mRNA decay pathways and aberrant translation events in mESCs
- XRN1 mediates mRNA turnover, whereas SKIV2L acts widely in translation surveillance
- AVEN interacts with ribosomes and the Ski complex and counteracts ribosome stalling
- Histone mRNAs, uORFs, and small ORFs are key targets of SKIV2L and AVEN

Tuck et al., 2020, Molecular Cell *77*, 1222–1236
March 19, 2020 © 2020 The Author(s). Published by Elsevier Inc.
https://doi.org/10.1016/j.molcel.2020.01.007

Molecular Cell
Article

Mammalian RNA Decay Pathways Are Highly Specialized and Widely Linked to Translation

Alex Charles Tuck,[1,4] Aneliya Rankova,[1,4] Alaaddin Bulak Arpat,[3] Luz Angelica Liechti,[3] Daniel Hess,[1] Vytautas Iesmantavicius,[1] Violeta Castelo-Szekely,[3] David Gatfield,[3] and Marc Bühler[1,2,5,*]
[1]Friedrich Miescher Institute for Biomedical Research, Maulbeerstrasse 66, 4058 Basel, Switzerland
[2]University of Basel, Petersplatz 10, 4003 Basel, Switzerland
[3]Center for Integrative Genomics, University of Lausanne, 1015 Lausanne, Switzerland
[4]These authors contributed equally
[5]Lead Contact
*Correspondence: marc.buehler@fmi.ch
https://doi.org/10.1016/j.molcel.2020.01.007

SUMMARY

RNA decay is crucial for mRNA turnover and surveillance and misregulated in many diseases. This complex system is challenging to study, particularly in mammals, where it remains unclear whether decay pathways perform specialized versus redundant roles. Cytoplasmic pathways and links to translation are particularly enigmatic. By directly profiling decay factor targets and normal versus aberrant translation in mouse embryonic stem cells (mESCs), we uncovered extensive decay pathway specialization and crosstalk with translation. XRN1 (5′-3′) mediates cytoplasmic bulk mRNA turnover whereas SKIV2L (3′-5′) is universally recruited by ribosomes, tackling aberrant translation and sometimes modulating mRNA abundance. Further exploring translation surveillance revealed AVEN and FOCAD as SKIV2L interactors. AVEN prevents ribosome stalls at structured regions, which otherwise require SKIV2L for clearance. This pathway is crucial for histone translation, upstream open reading frame (uORF) regulation, and counteracting ribosome arrest on small ORFs. In summary, we uncovered key targets, components, and functions of mammalian RNA decay pathways and extensive coupling to translation.

INTRODUCTION

RNA decay ensures steady-state mRNA expression, eliminates aberrant transcripts, and remodels the transcriptome upon changing conditions (Bresson et al., 2017; Pérez-Ortín et al., 2013; Sohrabi-Jahromi et al., 2019; Tuck and Tollervey, 2013). In the nucleus, mRNAs are mainly degraded 3′–5′ by the exosome complex, assisted by factors including the helicase Mtr4 (MTR4) (Kilchert et al., 2016; LaCava et al., 2005; Mitchell et al., 1997; Schmid and Jensen, 2018). Cytoplasmic mRNA turnover is initiated by poly(A) tail removal and proceeds via 3′–5′ exoribonucleolysis by the exosome or decapping followed by 5′–3′ degradation by the exoribonuclease Xrn1 (XRN1) (Hsu and Stevens, 1993; Łabno et al., 2016; Parker, 2012; Zinder and Lima, 2017). Cytoplasmic exosome activity requires the Ski complex (Anderson and Parker, 1998), comprising the scaffold Ski3 (TTC37), two copies of Ski8 (WDR61), and the helicase Ski2 (SKIV2L). Ski2, like its homolog Mtr4, unwinds RNA and channels it to the exosome (Halbach et al., 2013). Many pathologies are linked to dysregulation of these factors. For example, XRN1 is downregulated in osteosarcoma (Pashler et al., 2016), exosome mutations are linked to cancer (Robinson et al., 2015) and neurological disorders (Morton et al., 2018), and Ski complex impairment causes a congenital bowl disorder (Fabre et al., 2012; Hartley et al., 2010).

The complexity of RNA decay makes it hard to study and fundamental questions remain. For example, do decay pathways act redundantly or target specific transcripts? If the latter, how is specificity achieved, and what advantage does it confer? Analyses of *S. cerevisiae* mutants suggest that Xrn1 contributes more than the exosome to cytoplasmic turnover (Parker, 2012). However, compensation between decay pathways and secondary effects make it unclear whether this reflects the physiological situation. Furthermore, higher eukaryotes have extra factors and pathways, including 3′ uridyltransferases acting in cytoplasmic decay (Łabno et al., 2016; Lim et al., 2014) and diverse MTR4-containing nuclear exosome adaptor complexes (Lubas et al., 2011; Meola et al., 2016).

A further challenge is that RNA decay is coupled to other RNA life cycle events. For example, the nuclear exosome is recruited during transcription to remove early termination products, introns, or full-length mRNAs (Kilchert et al., 2016). In the cytoplasm, there is crosstalk between translation and RNA decay, epitomized by surveillance pathways targeting mRNAs with premature termination codons (nonsense-mediated decay [NMD]), translational roadblocks (no-go decay [NGD]), or no stop codon (nonstop decay [NSD]) (Roy and Jacobson, 2013). A key event is mRNA cleavage at stalled ribosomes, which generates 5′ and 3′ RNA fragments that are cleared by the exosome and Xrn1 (Gatfield and Izaurralde, 2004; Ghosh and Jacobson, 2010; Guydosh and Green, 2017). Coupling between translation and degradation could be widespread and extend beyond surveillance (Ibrahim et al., 2018). In support of this, Xrn1 can act co-translationally (Hu et al., 2009; Pelechano et al., 2015), and

 © 2020 The Author(s). Published by Elsevier Inc.
This is an open access article under the CC BY license (http://creativecommons.org/licenses/by/4.0/).

structures capture the yeast Ski complex or Xrn1 bound to ribosomes (Schmidt et al., 2016; Tesina et al., 2019). There is intense interest in understanding whether decay factor interactions with the ribosome are conserved in higher eukaryotes, the functional relevance, and whether this constitutes a major decay route.

Here, we address key questions about mammalian mRNA decay. First, what are the physiological targets of major decay pathways? Second, focusing on cytoplasmic decay, to what extent is this coupled to translation, and what factors influence this? To reveal direct, physiological targets of decay factors, we used crosslinking and analysis of cDNAs (CRAC) to compare the transcriptome-wide interactions of XRN1, SKIV2L, and MTR4 in mouse embryonic stem cells (mESCs). Our data suggest that most mRNA turnover occurs via the 5′–3′ pathway, but some mRNAs (particularly those encoding histones) depend on cytoplasmic 3′–5′ decay. We find that SKIV2L and XRN1 directly bind ribosomes, and translation appears to assist bulk mRNA turnover by XRN1. Strikingly, SKIV2L is specifically and pervasively recruited to ribosome-occupied regions, suggesting it acts exclusively in translation-associated mRNA surveillance. Our data reveal triggers of ribosome stalling and SKIV2L recruitment, which we explore by globally mapping stalled ribosomes. Proteomic analyses identify the RNA-binding factor AVEN and uncharacterized protein FOCAD as Ski complex interactors. We observe AVEN binding to GC-rich RNAs predicted to be structured and increased SKIV2L binding, decay, and ribosome stalling at these regions upon *Aven* knockout. We conclude that AVEN and SKIV2L cooperate to counteract aberrant translation, with AVEN preventing ribosome stalls at structured regions and SKIV2L eliminating transcripts if these events accumulate. The AVEN-SKIV2L pathway acts on diverse substrates, including histone mRNAs, upstream open reading frames (uORFs), and small ORF (sORF)-containing RNAs. In summary, we uncover specialization between mammalian RNA decay pathways and widespread crosstalk with translation and establish SKIV2L and AVEN as components of a universal translation surveillance program.

RESULTS

Mammalian RNA Decay Pathways Target Distinct Transcripts

To examine the specificity of RNA decay pathways (Figure 1A), we applied the CRAC approach to SKIV2L, XRN1, and MTR4 in mESCs (Granneman et al., 2009; Tuck et al., 2018). After endogenously 3xFLAG-Avi tagging these proteins (Figure S1A; Table S1) (Flemr and Bühler, 2015), we crosslinked cells with UV (254 nm) to fix protein-RNA interactions, purified ribonucleoproteins (RNPs) under denaturing conditions, performed a limited RNase digestion, and sequenced the RNA fragments (Figure 1B). We performed five or six technical replicates (including three published MTR4 datasets; Table S2). Global comparison of mRNAs bound by SKIV2L, MTR4, and XRN1 using principal-component analysis (PCA) or correlation coefficients revealed highly reproducible differences (Figures 1C, 1D, and S1B; Table S3). To explore the specificity of individual transcripts, we used t-distributed stochastic neighbor embedding (t-SNE) to arrange mRNAs by relative binding to the three proteins (Figure 1E). Although some transcripts bound similarly to SKIV2L, XRN1, and MTR4 (e.g., *Trim28*; Figure 1F), others had a clear preference (e.g., *Sfpq* or *Pim3*; Figure 1F), suggesting that for many transcripts, one decay route dominates. Furthermore, functionally related mRNAs shared binding preferences (Figures 1E and S1D) (e.g., histone mRNAs bound abundantly to SKIV2L).

As XRN1-dependent 5′–3′ decay is assumed to be the main determinant of RNA half-life and steady-state abundance we were intrigued by transcripts bound highly by SKIV2L (e.g., Figure 1G). SKIV2L assists the exosome in 3′–5′ decay, and a co-immunoprecipitation confirmed that 3xFLAG-Avi-tagged SKIV2L interacts with the cytoplasmic exosome component DIS3L (Figure S1C). We therefore suspected that highly SKIV2L-bound mRNAs are degraded in a 3′–5′ SKIV2L-dependent manner. To test this, we generated *Skiv2l^{fl/fl}* conditional knockout cells by integrating *loxP* sites into introns 10 and 17 in CreERT2-expressing mESCs (Flemr and Bühler, 2015) (Figure 1H). We treated these cells with 4-hydroxytamoxifen (4OHT) to induce *loxP* recombination and production of truncated SKIV2L without a catalytic domain (Figures 1H and S1E) and profiled gene expression by RNA sequencing (RNA-seq) (Table S3). There were many changes after 6 days of 4OHT treatment, but these did not correlate with SKIV2L CRAC (Figure 1I, right) so are likely indirect effects. Conversely, after 4 days of 4OHT treatment, transcript upregulation correlated with SKIV2L CRAC (Figure 1I, left, and Figure S1F). Measuring transcriptome-wide half-lives following transcription shut off by actinomycin D confirmed that highly SKIV2L-bound transcripts are stabilized upon *Skiv2l* knockout, exemplified by replication-dependent histone mRNAs (Figure S1G; Table S3). Some stabilized SKIV2L targets (e.g., *Calr* and *Pdia4*; Figure S1G) did not increase in abundance (Figure 1I), suggesting that cells partially compensate for the loss of SKIV2L. Of note, high-confidence SKIV2L targets (Figure S1G) were expressed at wild-type (WT) levels in our tagged cell lines, confirming that tagged SKIV2L is functional (Figure S1H). We conclude that SKIV2L-dependent 3′–5′ decay contributes to the steady-state abundance of a subset of mRNAs, including most replication-dependent histone mRNAs. Our approach thus reveals physiological targets of mRNA decay pathways.

Cytoplasmic RNA Decay Is Widely Influenced by Translation

As cytoplasmic decay pathways are less well studied, we now focused on XRN1 and SKIV2L. A key question is to what extent they interact with translation. Remarkably, CRAC reads mapping to ribosomal RNA revealed specific, reproducible binding of SKIV2L and XRN1 to the 40S subunit mRNA entry and exit regions (Figure 2A), resembling yeast structures (Schmidt et al., 2016; Tesina et al., 2019). Therefore, SKIV2L and XRN1 ribosome interactions are conserved to mammals and occur in unperturbed cells.

To explore whether SKIV2L and/or XRN1 activity is widely coupled to translation, we examined binding across individual mRNAs (e.g., Figures 1F and 1G). SKIV2L binding was strongly biased toward regions occupied by ribosomes, i.e., 5′ UTRs, coding sequences (CDSs), and uORFs (e.g., *Ifrd1*; Figure 1G).

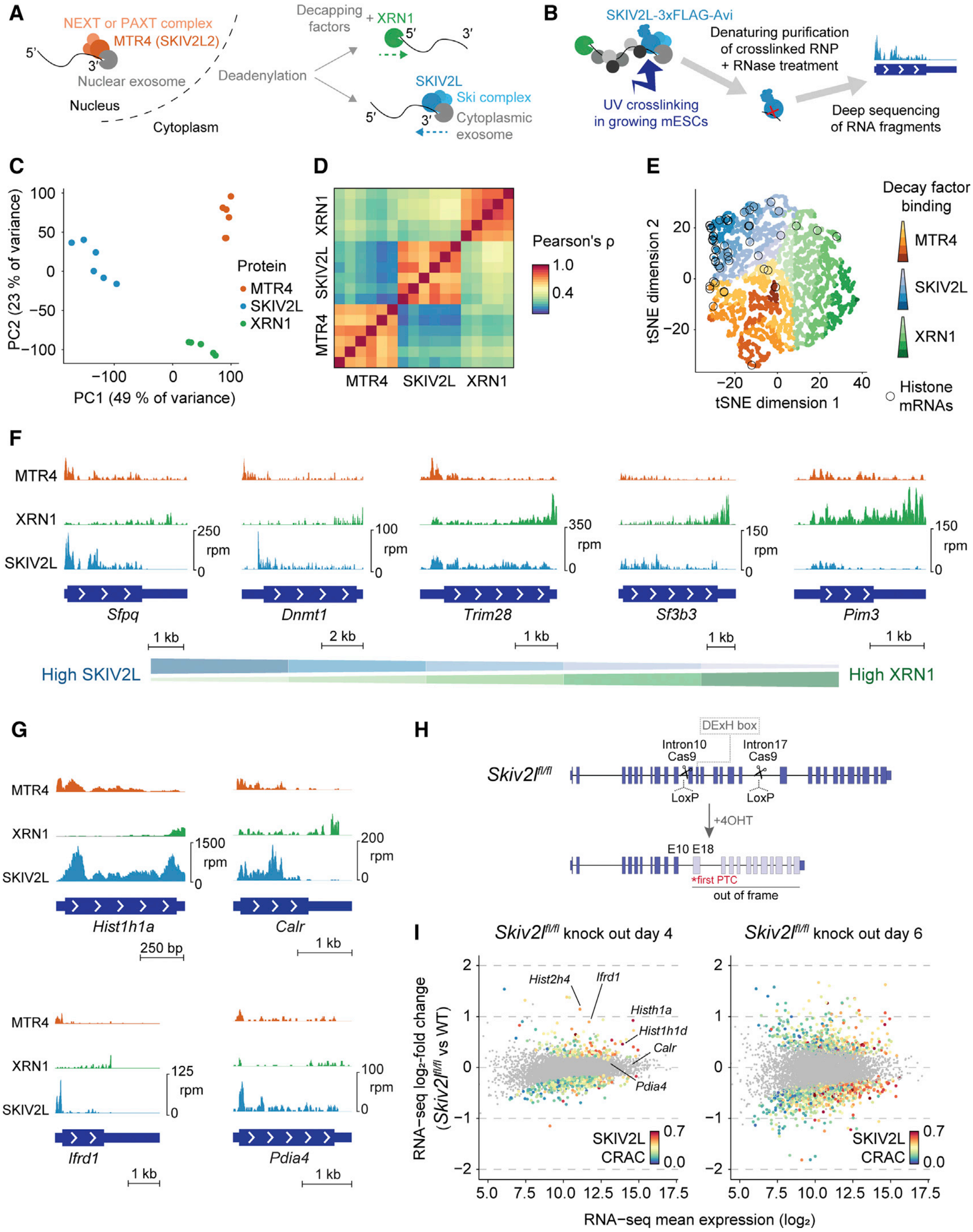

Figure 1. Mammalian mRNA Decay Pathways Target Distinct Transcripts

(A) RNA decay pathways.

(B) CRAC outline.

(C and D) PCA (C) and correlation matrix (D) based on decay factor binding (CRAC counts) to mRNAs. Replicates correspond to separate experiments for the same cell line.

(E) t-SNE representation of mRNAs based on relative binding to MTR4, SKIV2L, and XRN1.

(F and G) CRAC coverage across individual mRNAs. Transcripts in (F) illustrate different XRN1:SKIV2L ratios, whereas (G) depicts transcripts highlighed in panel (I).

(H) Conditional knockout strategy for *Skiv2l*.

(I) Differential expression analysis for *Skiv2l* knockout for the mRNAs in (E), with significantly changing transcripts (DESeq2 padj < 0.05) colored by SKIV2L binding (as in E).

See also Figure S1 and Tables S1, S2, and S3.

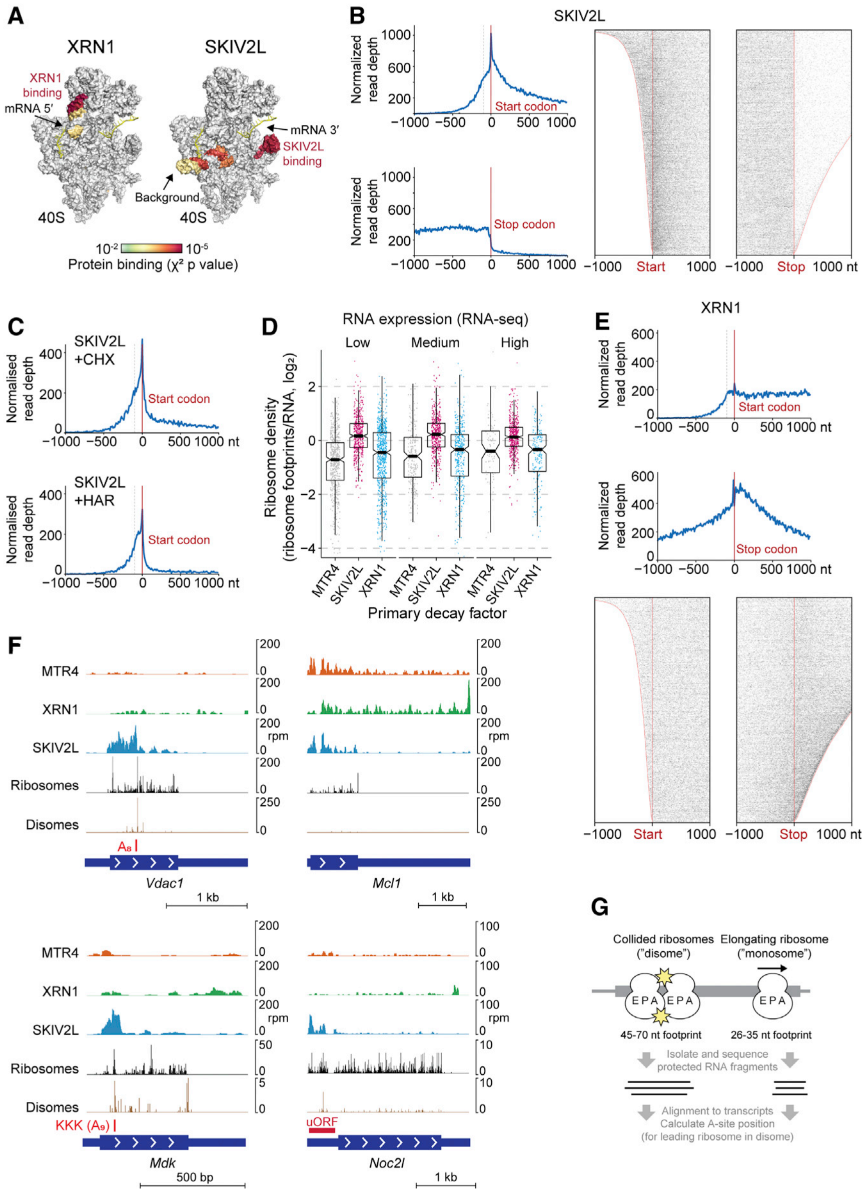

Figure 2. Cytoplasmic mRNA Decay Is Widely Influenced by Translation

(A) CRAC signal for SKIV2L and XRN1 on the ribosomal 40S subunit, based on the mouse rRNA sequence and human structure (Khatter et al., 2015). Significantly bound regions are colored by χ^2 p value, and the mRNA path (yellow) is taken from Schmidt et al. (2016).

(B and C) CRAC signal for SKIV2L around start and stop codons, summed (left) or for individual mRNAs (right). Data in (B) correspond to untreated cells, whereas those in (C) correspond to 30-min cycloheximide or harringtonine treatment.

(D) Ribosome densities for mRNAs grouped by expression and most abundantly bound decay factor (defined in Figure 1E).

(E) XRN1 CRAC signal around start and stop codons.

(F) CRAC, monosome, and disome profiling for individual mRNAs.

(G) Monosome and disome profiling approach.

See also Figure S2 and Table S4.

Global analysis of binding around start and stop codons (Figure 2B) revealed this pattern is universal. Treating cells with translation inhibitors led to a redistribution of SKIV2L binding (Figure 2C) that parallels changes in ribosome occupancy, confirming that active translation directs SKIV2L binding. Harringtonine blocks translation post-initiation to deplete ribosomes from CDS regions, where we observed loss of SKIV2L binding. In contrast, cycloheximide stalls elongating ribosomes, leading to queuing and initiation upstream of the canonical start codon (Kearse et al., 2019). Consistently, SKIV2L accumulated in 5′ UTRs (Figure 2C). Further supporting the role of ribosomes in recruiting SKIV2L, we found that SKIV2L CRAC correlates with the number of ribosomes on a transcript, which we measured by ribosome profiling (Figure 2D; Table S4). We conclude that SKIV2L is specifically and universally recruited to translated regions via ribosome interactions.

In contrast to SKIV2L, XRN1 bound the full length of mRNAs, consistent with its major role being in bulk mRNA turnover. Strong XRN1 enrichment in 3′ UTRs (Figure 2E) supports a model where XRN1 follows the last translating ribosome, which helps remove obstacles. In the 3′ UTR, XRN1 may stall at RNA structures or protein-bound sites. The pattern of XRN1 binding around the stop codon is less well defined than that of SKIV2L, supporting this looser relationship with the ribosome. We conclude that both cytoplasmic decay pathways are widely influenced by translation, but only XRN1 degrades full-length mRNAs.

SKIV2L Functions in Universal Translation Surveillance

We next sought to identify translation events leading to SKIV2L recruitment. Unlike the relatively even ribosome profiling coverage across mRNAs, SKIV2L CRAC signal was enriched at specific sites (e.g., Figure 2F). Cytoplasmic 3′–5′ decay acts in many surveillance pathways (e.g., NMD, NGD, and NSD), so we suspected that SKIV2L peaks reflect RNA features that arrest or stall ribosomes. Endonucleolytic cleavage at ribosome stall sites (D'Orazio et al., 2019; Gatfield and Izaurralde, 2004; Glover et al., 2019; Guydosh and Green, 2017) may enable SKIV2L to engage the 3′ end of the upstream fragment (Schmidt et al., 2016). Consistent with this, some SKIV2L-bound RNA fragments had non-templated 3′ U-tails (Figure S2A). Uridylation facilitates mRNA degradation by XRN1, DIS3L2, and the exosome (Lim et al., 2014; Slevin et al., 2014) and may act as a landing pad for SKIV2L. The U-tails confirm that SKIV2L binds cleaved RNAs. We also found U-tails on XRN1-bound RNA fragments, consistent with 5′–3′ and 3′–5′ pathways being able to act on a single

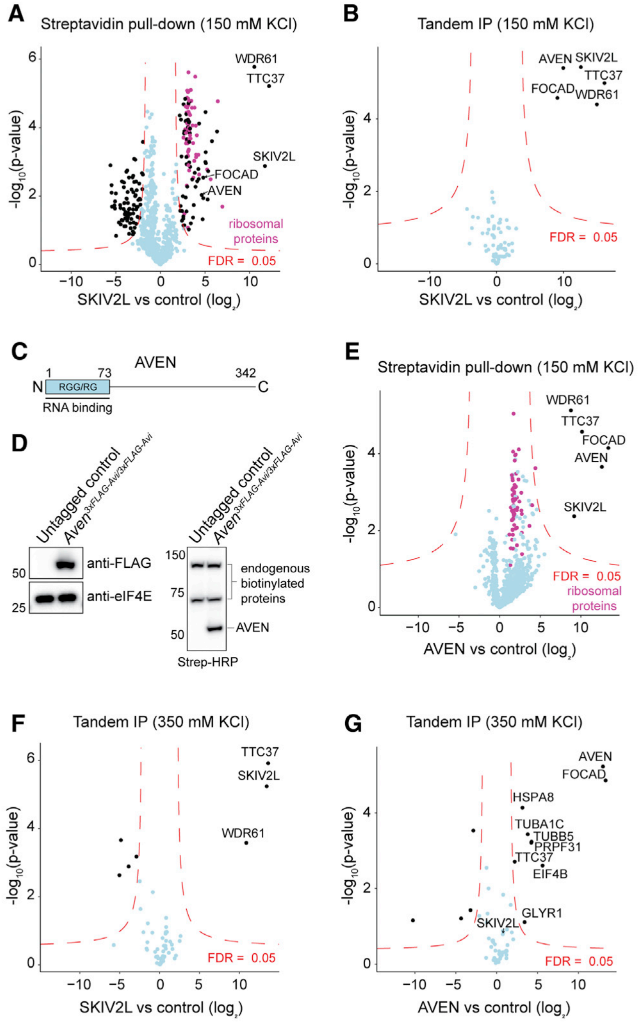

Figure 3. AVEN and FOCAD are Ski Complex Interactors

(A and B) Mass spectrometry (MS) of streptavidin (A) or tandem FLAG-streptavidin (B) purification of 3xFLAG-Avi-SKIV2L.

(C) Mouse AVEN protein.

(D) Western blot analysis of endogenously tagged *Aven*$^{3xFLAG\text{-}Avi/3xFLAG\text{-}Avi}$ expression and biotinylation.

(E) MS of streptavidin purification of 3xFLAG-Avi-AVEN.

(F and G) MS of tandem FLAG-streptavidin purification of 3xFLAG-Avi-SKIV2L (F) and 3xFLAG-Avi-AVEN (G) using high salt. All experiments include RNase treatment, three technical replicates, and untagged mESCs as a control. FDR, false discovery rate.

See also Table S5.

mRNA and as reported by studies of yeast antiviral activity (Widner and Wickner, 1993) and for histone mRNAs (Mullen and Marzluff, 2008).

We reasoned that 3′ ends of SKIV2L-bound RNA fragments should reveal endogenous triggers of ribosome stalling. Indeed, 3′ ends were enriched at specific codon pairs, including those encoding lysine-lysine or proline (Figure S2B). Enrichment at proline codons was weak but had a clear frame preference, corroborating reports that proline in the nascent peptide triggers stalling (Ingolia et al., 2011; Pavlov et al., 2009). Examining longer codon runs, SKIV2L binding was elevated at poly-proline, -lysine, -glutamate, -aspartate, and -arginine (Figure S2C; *Mdk* in Figure 2F). These preferences resemble codons reported to stall ribosomes based upon mESC ribosome profiling (Ingolia et al., 2011). As SKIV2L peaks occurred at purine-rich codon runs, we suspected that for these, the RNA sequence is more important than the amino acid. Examining runs of ≥12 purines, SKIV2L enrichment was equivalent at lysine-rich and lysine-poor sequences but more pronounced at A-rich than G-rich sequences (Figure S2D). This suggests that A-rich sequences trigger ribosome stalling and SKIV2L surveillance, as exemplified by *Vdac1* and *Mdk* (Figure 2F, red boxes), and agrees with a reporter-based study (Arthur et al., 2015). XRN1 showed slight enrichment at some of these sites (Figures S2C and S2D), likely reflecting a minor role in surveillance.

To verify that SKIV2L-bound sites reflect ribosome stalls, we used a new method (disome profiling) to map collided ribosome pairs (disomes) (Figure 2G) (Arpat et al., 2019). Disomes form at ribosome stall sites (Ikeuchi et al., 2019; Juszkiewicz et al., 2018; Simms et al., 2017) and can be identified from 45- to 70-nt protected RNA fragments (Arpat et al., 2019; Guydosh and Green, 2014). We also performed standard ribosome profiling (monosome profiling). We calculated A-site positions of monosomes and leading ribosomes in disomes (Figure 2G). This revealed disome enrichments over codon and sequence runs (Figures S2C and S2D) and individual sites (e.g., *Vdac1*, *Mdk*, and *Noc2l*; Figure 2F) with elevated SKIV2L binding, confirming these reflect ribosome stalling. In some cases (e.g., polyproline; Figure S2C), the disome signal was stronger than the SKIV2L CRAC signal. This suggests that some stalls potently trigger RNA cleavage, but others (e.g., polyproline) are resolved without mRNA decay.

We conclude that although SKIV2L and XRN1 can target the same transcript, their roles are highly specialized. XRN1 mediates bulk mRNA decay, with a minor surveillance role, whereas SKIV2L responds exclusively to aberrant translation.

AVEN and FOCAD Are Ski Complex Interactors

We next wondered if SKIV2L is recruited solely by ribosome and mRNA interactions or if other factors participate. MTR4 is targeted by adaptor proteins, so analogous Ski complex adapters could also exist. To identify SKIV2L interactors, we performed streptavidin affinity purification (including RNase treatment) and immunoprecipitation mass spectrometry (IP-MS). Using tagged SKIV2L as bait, we identified various RNA binders, Ski complex components WDR61 and TTC37, and ribosomal proteins (Figure 3A; Table S5), consistent with the SKIV2L-ribosome interaction detected by CRAC (Figure 2A). To enrich for more direct SKIV2L interactions, we repeated the experiment adding a FLAG immunoprecipitation (tandem IP-MS). This recovered just two proteins, AVEN and FOCAD (KIAA1797), besides the Ski complex (Figure 3B).

FOCAD is a poorly characterized protein whose loss is associated with glioma (Brockschmidt et al., 2012) and colorectal cancer (Weren et al., 2015). Remarkably, its *Arabidopsis* homolog binds the Ski complex (Lange et al., 2019). AVEN is widely expressed and contributes to acute leukemia/lymphoma (Eißmann et al., 2013). Its disordered N-terminal glycine- and arginine-rich (RGG/RG) domain (Figure 3C) interacts with RNA

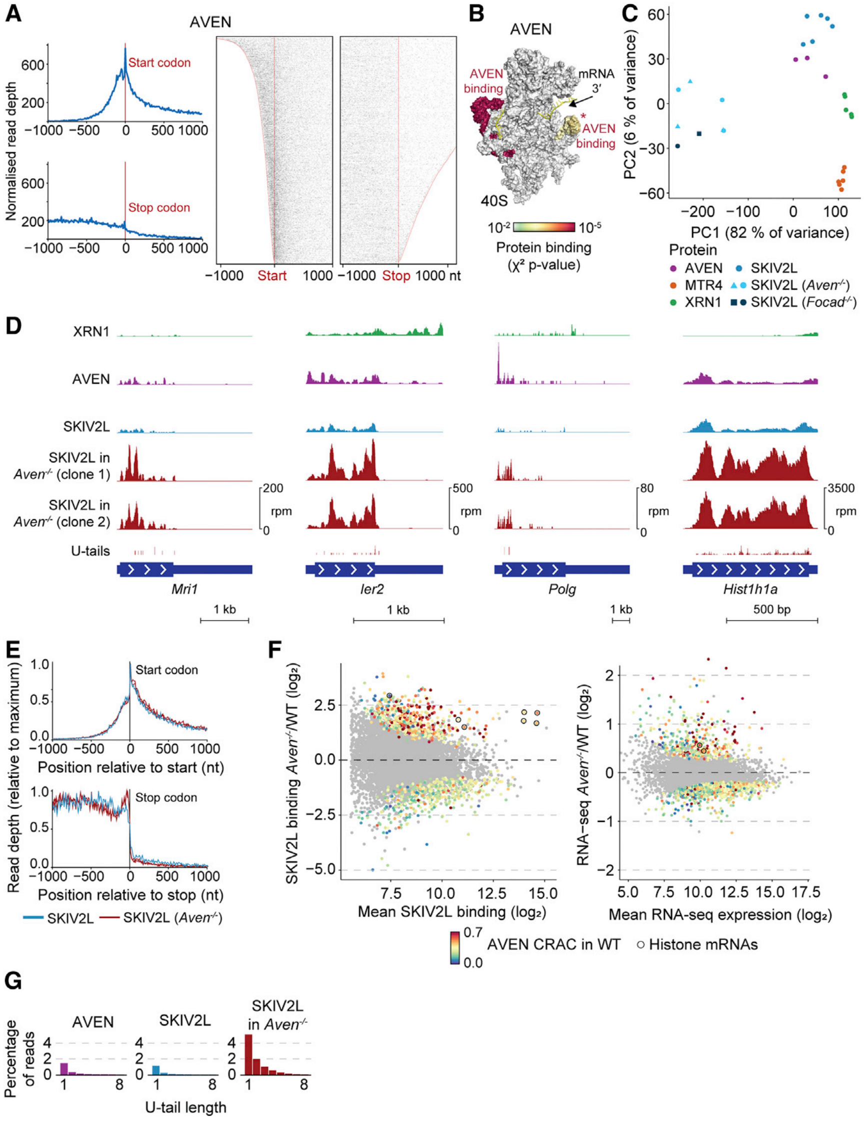

Figure 4. SKIV2L Binding and 3′–5′ Decay Increase upon *Aven* Knockout

(A and B) CRAC signal for AVEN around start and stop codons (A) and on the ribosomal 40S subunit (B).

(C) PCA based on mRNA counts. Shapes indicate different clones.

(D) CRAC coverage for individual mRNAs.

(E) SKIV2L CRAC around start and stop codons in WT and *Aven*$^{-/-}$ cells.

(F) Changes in SKIV2L CRAC binding (left) and RNA-seq counts (right) for *Aven*$^{-/-}$ versus WT cells. Significantly up/downregulated transcripts (padj < 0.05) are colored by AVEN CRAC counts in WT cells, relative to SKIV2L+XRN1+MTR4 counts, and replication-dependent histone mRNAs are circled.

(G) Proportion of AVEN or SKIV2L CRAC reads in mRNAs with 3′ U-tails.

See also Figure S3 and Table S3.

and localizes AVEN to polysomes (Thandapani et al., 2015). Furthermore, AVEN aids translation through G-quadruplexes in two mRNAs, and IP-MS using human AVEN as bait retrieved the Ski complex and FOCAD (Thandapani et al., 2015). These studies support our MS results.

To confirm the SKIV2L-AVEN interaction, we endogenously 3xFLAG-Avi-tagged *Aven* (Figure 3D) and performed IP-MS with AVEN as bait, recovering the Ski complex and FOCAD (Figure 3E). We repeated the tandem IP-MS using a higher salt concentration and SKIV2L or AVEN as bait (Figures 3F and 3G). AVEN now recovered FOCAD, but not the Ski complex, suggesting AVEN-FOCAD and SKIV2L-WDR61-TTC37 (Ski complex) are separable complexes that associate transiently with each other.

SKIV2L Binding and 3′–5′ Decay Increase upon *Aven* Knockout

As AVEN associates with polysomes (Thandapani et al., 2015) and the Ski complex (Figure 3B), we speculated it might recruit SKIV2L to targets. To test this, we performed CRAC on 3xFLAG-Avi-tagged AVEN mESCs to map AVEN-binding sites. Like SKIV2L, AVEN bound the 5′ UTR and CDS of mRNAs (Figure 4A), albeit with a stronger 5′ bias. AVEN CRAC also revealed ribosome contacts, one overlapping that of SKIV2L (Figure 4B, marked with an asterisk [*]). PCA based on mRNA binding revealed that AVEN and SKIV2L bound common targets (Figures 4C and S3A), and AVEN and SKIV2L bound similar regions on individual mRNAs (Figure 4D). These similarities suggest that AVEN and SKIV2L function in the same pathway.

To determine whether AVEN affects Ski complex recruitment to mRNAs, we generated *Aven*$^{-/-}$ mESCs by deleting the C-terminal portion of the protein (Figure S3B). This led to near-complete knockdown of the entire *Aven* mRNA (Figure S3B). SKIV2L CRAC revealed that while the average binding pattern of SKIV2L along mRNAs was unaffected in *Aven*$^{-/-}$ (Figure 4E), there were strong differences in which mRNAs were bound, apparent from a PCA (Figures 4C and S3A). AVEN thus plays a role in SKIV2L targeting. SKIV2L binding was similarly perturbed in *Focad*$^{-/-}$ mESCs (Figures 4C and S3C), suggesting that AVEN and FOCAD functionally overlap. Due to its size and low abundance, FOCAD was challenging to work with, so we focused on AVEN.

In contrast to our prediction, SKIV2L binding to mRNAs was not reduced in *Aven*$^{-/-}$ cells but instead increased at many sites (Figure 4F; examples in Figure 4D). To account for changes in RNA abundance, we normalized CRAC to RNA-seq counts from WT and *Aven*$^{-/-}$ cells (Table S3). Increased SKIV2L binding was accompanied by elevated 3′ uridylation of bound RNAs (Figure 4G), indicating increased 3′–5′ decay. This suggests that

unlike WT conditions, where SKIV2L transiently scans all translation events, upon *Aven* deletion, SKIV2L assists intensively in 3′–5′ decay at specific sites. These sites are bound by AVEN in WT cells (Figure 4F, left, and Figure S3D), exemplified by replication-dependent histone mRNAs (circled in Figure 4F), suggesting that changes in SKIV2L binding are a direct consequence of losing AVEN. Changes in mRNA levels in *Aven*$^{-/-}$ cells (Figure 4F, right) were smaller than changes in SKIV2L CRAC and correlate poorly with AVEN binding (Figure S3E) so likely represent secondary effects.

In summary, AVEN does not recruit the Ski complex. Instead, loss of AVEN increases SKIV2L binding and 3′–5′ RNA decay at many sites. As aberrant translation events recruit SKIV2L and AVEN may assist translation (Thandapani et al., 2015), we hypothesize that AVEN prevents ribosome stalls that otherwise trigger SKIV2L binding and mRNA decay.

AVEN and SKIV2L Counteract Ribosome Stalling

To globally assess how AVEN affects translation and ribosome stalling, we performed monosome and disome profiling for WT and *Aven*$^{-/-}$ mESCs (Table S4). We plotted changes in mRNA disome and monosome densities (Figure 5A), distinguishing mRNAs with increased, decreased, or unchanged SKIV2L binding in *Aven*$^{-/-}$ versus WT (pink/blue/gray points in Figure 5A) and calculated best fit lines. This revealed that in *Aven*$^{-/-}$, changes in monosome and disome density occur for all categories of mRNAs and are correlated, as expected. However, on top of these changes, mRNAs gaining SKIV2L binding in *Aven*$^{-/-}$ display a further increase in disome occupancy (upward shift of pink points in Figure 5A; exemplified by replication-dependent histone mRNAs in Figures 5B and 5C). mRNAs accumulating disomes upon *Aven* knockout were bound by AVEN in WT (Figure S4A), and disome changes in individual transcripts overlapped with AVEN and SKIV2L binding (Figure 5C). These data suggest that stalled ribosomes accumulating in *Aven*$^{-/-}$ drive increased SKIV2L recruitment, which presumably clears these mRNAs.

According to this model, the combined absence of AVEN and SKIV2L should have an additive effect, as SKIV2L would not be available to clear stalled messenger RNPs (mRNPs) arising in the absence of AVEN. AVEN targets should thus be stabilized and accumulate in a double knockout. To test this, we generated a *Skiv2l*$^{fl/fl}$ conditional knockout in *Aven*$^{-/-}$ mESCs and performed RNA-seq after 4OHT treatment (Figure S4B). In contrast to the single *Skiv2l*$^{fl/fl}$ knockout, where transcripts accumulated after 4 days of 4OHT (Figure 1I), we observed widespread changes in *Aven*$^{-/-}$ *Skiv2l*$^{fl/fl}$ after 2 days (Figure 5D; Table S3). Upregulated transcripts displayed high AVEN binding in WT (Figure 5D, left) and increased SKIV2L occupancy in *Aven*$^{-/-}$ (Figure 5D, right), suggesting they are direct SKIV2L and AVEN targets. Transcriptome-wide half-life measurements following actinomycin D transcription shut off confirmed that these targets are stabilized in the double knockout (Figure S4C; Table S3). The accumulation of replication-dependent histone mRNAs was particularly striking (Figures 5D, circled). These results support a model whereby AVEN and SKIV2L cooperate in translation-coupled RNA surveillance, with AVEN opposing translational stalls and SKIV2L eliminating mRNAs if aberrant events accumulate. Furthermore, SKIV2L and AVEN maintain normal histone translation and RNA levels.

AVEN and SKIV2L Affect Expression of Many mRNAs

As replication-dependent histone levels are coupled to DNA synthesis, with histone mRNAs accumulating until they are degraded at the end of S-phase, we suspected that perturbed histone expression in the absence of AVEN and SKIV2L might alter cell-cycle progression. To test this, we synchronized mESCs at G1/S using a double thymidine block and monitored DNA content by DAPI staining following release (Figure S4D). *Aven*$^{-/-}$ *Skiv2l*$^{fl/fl}$ double knockout cells exhibited delayed progression through S phase, into G2, and ultimately into G1, in line with their altered histone mRNA abundance (compared to WT or single knockouts). We conclude that the AVEN-SKIV2L pathway contributes to cell cycle progression.

While examining individual mRNAs, we noticed that besides main CDS regions, SKIV2L and ribosomes also accumulate in uORFs in *Aven*$^{-/-}$ (Figures 5E and 5F). AVEN occupied these uORFs in WT cells (Figure 5F), and increased ribosome occupancy in *Aven*$^{-/-}$ cells correlated with WT AVEN binding (Figure S4E) and increased SKIV2L binding in *Aven*$^{-/-}$ (Figure 5E). Whereas *Aven* knockout resulted in increased disome occupancy in main CDS regions, changes across uORFs occurred for monosomes, disomes, or both. AVEN thus has a complex effect on 5′ UTR translation.

As uORF translation can alter mRNA stability or main CDS translation (Calvo et al., 2009), we wondered whether such changes occur upon loss of AVEN and/or SKIV2L. We focused on *Atf4* and *Ifrd1* mRNAs, with functional uORFs bound by AVEN and SKIV2L (Figure 5F). Under normal conditions, *Ifrd1* uORF translation destabilizes the mRNA via NMD (Zhao et al., 2010). *Ifrd1* RNA accumulated after 4 days of *Skiv2l* knockout and 2 days of *Aven Skiv2l* double knockout (Figure 5G), suggesting that SKIV2L participates in *Ifrd1* mRNA clearance, and this is enhanced by increased uORF ribosome occupancy in *Aven*$^{-/-}$.

In contrast to the destabilizing effect of the *Ifrd1* uORF, within *Atf4*, two uORFs modulate translation of the main CDS (Harding et al., 2000; Vattem and Wek, 2004). Ribosomes normally translate uORF1 then reinitiate at uORF2, preventing them from translating the main CDS, but during the integrated stress response (ISR), phosphorylation of the translation factor eIF2α reduces preinitiation complex availability. Ribosomes now scan past uORF2 and reinitiate at the downstream main CDS, producing ATF4 protein. To examine the effects of increased ribosome occupancy over *Atf4* uORFs in *Aven*$^{-/-}$, we monitored ATF4 accumulation upon activation of the ISR with thapsigargin. Compared to WT cells, ATF4 levels peaked earlier in *Aven*$^{-/-}$, despite similar levels of eIF2α phosphorylation and basal ATF4 pre-induction (Figure S4F). Therefore, binding of AVEN and SKIV2L to uORFs modifies transcript stability (*Ifrd1*) and main CDS translation (*Atf4*).

In summary, the roles played by AVEN and SKIV2L in counteracting aberrant translation are crucial for expression of uORF-containing and histone mRNAs, among others.

AVEN Acts on Structured RNAs

We next asked what makes mRNAs dependent on AVEN. AVEN crosslinks to G-quadruplexes in *Mll1* and *Mll4* mRNAs

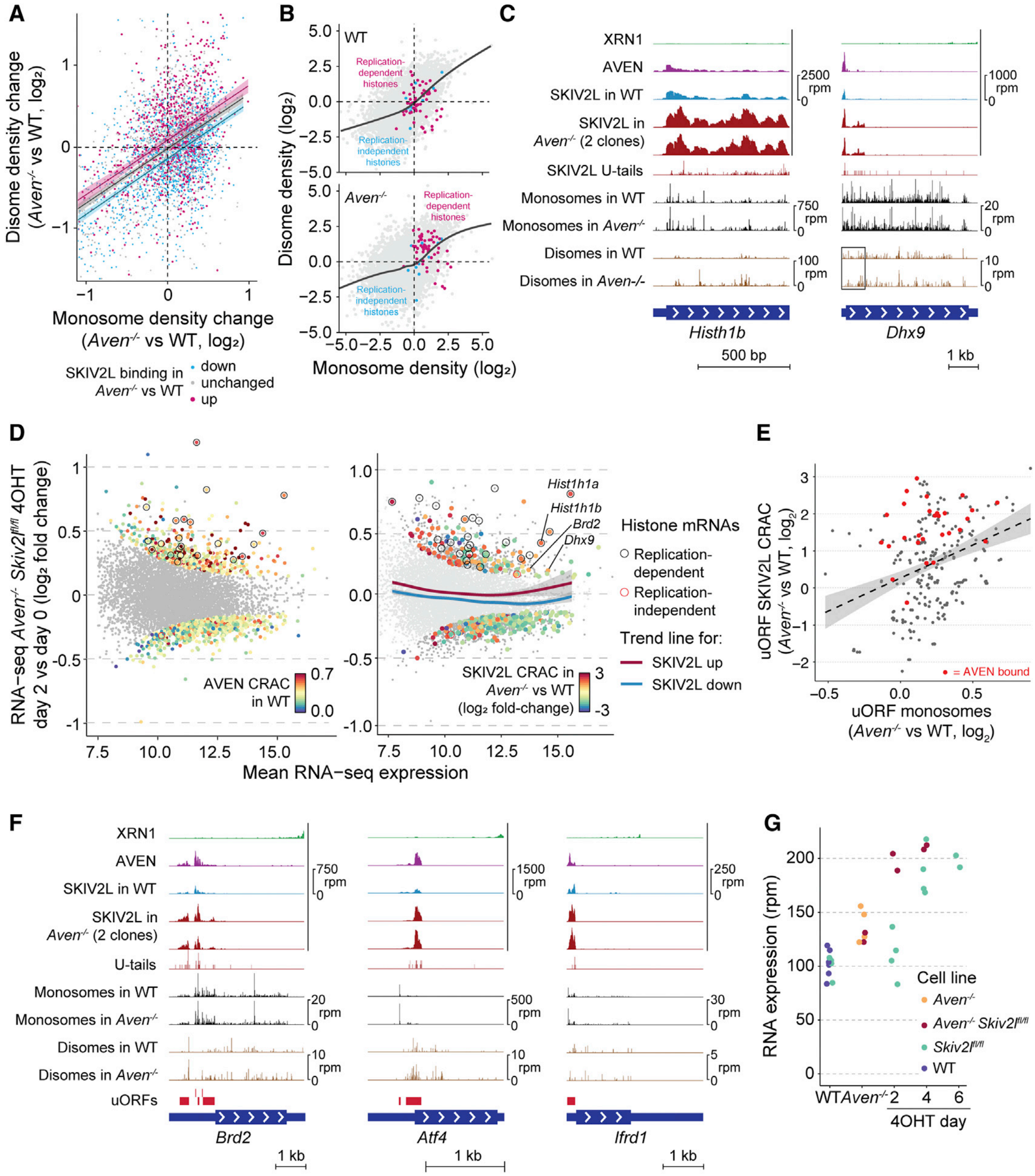

Figure 5. AVEN and SKIV2L Counteract Ribosome Stalling

(A) Changes in mRNA monosome and disome densities in *Aven*$^{-/-}$ versus WT. Transcripts are colored by changes in SKIV2L binding in *Aven*$^{-/-}$ versus WT (threshold $\log_2$ fold change = ±0.5; up, n = 1,856; down, n = 2,019; unchanged, n = 2,373), and a linear best fit is plotted for each group (shaded area represents 95% confidence interval).

(B) Monosome and disome densities in WT (top) and *Aven*$^{-/-}$ (bottom), highlighting histone mRNAs and with a cubic regression trendline.

(C) CRAC and monosome/disome profiling for individual mRNAs.

(D) Changes in mRNA abundance for *Skiv2l*$^{fl/fl}$ *Aven*$^{-/-}$ cells after 2-day 4-OHT. Significantly changing mRNAs (padj < 0.05) colored by AVEN CRAC in WT (left) or SKIV2L CRAC changes in *Aven*$^{-/-}$ versus WT (right). Cubic regression trendlines are shown for all mRNAs, grouped by increased/decreased SKIV2L binding in *Aven*$^{-/-}$ versus WT (right).

(E) Changes in uORF SKIV2L CRAC and monosome profiling counts for *Aven*$^{-/-}$ versus WT cells. Both datasets normalized to main CDS monosome profiling counts. A best-fit line is shown, with 95% confidence intervals, and AVEN-bound uORFs (defined in Figure S4E) are colored red.

(F) CRAC and monosome/disome profiling for individual mRNAs. uORFs identified from monosome profiling are shown in red.

(G) RNA-seq counts for *Ifrd1* in various cell lines showing individual replicates.

See also Figure S4 and Tables S3 and S4.

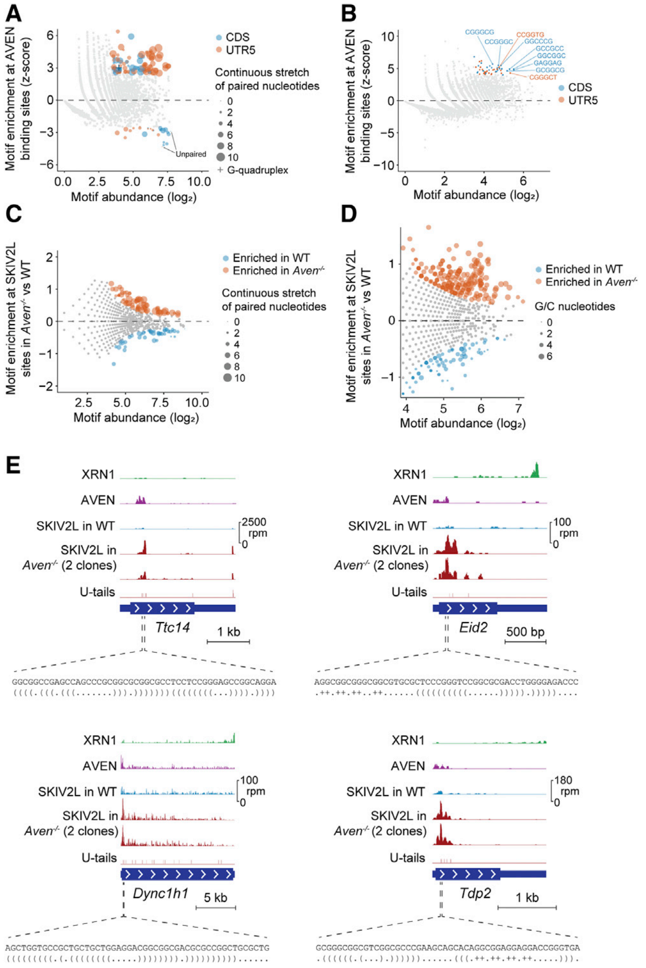

Figure 6. AVEN Acts on Structured RNAs

(A and B) Structure (A) and sequence motif analysis (B) for AVEN-binding sites, based on CRAC versus RNA-seq enrichments in 50-nt 5′ UTR and CDS windows. Points (structure motifs) in (A) are scaled by paired nucleotide content.

(C and D) Structure (C) and sequence motif analysis (D) for SKIV2L binding sites, comparing *Aven*$^{-/-}$ and WT cells. Points in (C) are scaled as for (A), and points in (D) are scaled by GC content.

(E) Examples of AVEN-bound windows showing their sequence, predicted structure (bracket/dot annotation for paired/unpaired nucleotides; + = G-quadruplex), and CRAC coverage for various proteins.

See also Figure S5.

(Thandapani et al., 2015), and RGG/RG motifs like AVEN's can melt G-rich or G-quadruplex sequences (Loughlin et al., 2019; Meyer et al., 2019). To test whether AVEN binds specific RNA sequences or structures, we examined the highest AVEN-bound 50-nt windows from each mRNA 5′ UTR and CDS (based on CRAC). Compared to control regions, AVEN-bound regions were enriched for stretches of paired nucleotides or G-quadruplexes, based on RNAfold predictions (Figure 6A), and GC-rich sequences (Figure 6B). The same was true of regions binding SKIV2L in *Aven*$^{-/-}$ (Figures 6C and 6D), and these preferences were clear for individual mRNAs (Figure 6E). This suggests that AVEN binding to GC-rich sites with structural propensity avoids sustained SKIV2L recruitment.

Interestingly, many SKIV2L-bound RNA fragments possessed 3′ U-tails in *Aven*$^{-/-}$ cells (Figure 4G), particularly where SKIV2L binding increased (Figure S5A). As U-tails are added to 5′ RNA cleavage products, we reasoned they could pinpoint sites of mRNA cleavage and decay in *Aven*$^{-/-}$. Indeed, U-tailed SKIV2L-bound RNA fragments in *Aven*$^{-/-}$ were enriched upstream of predicted structured regions (Figure S5B). Disomes aligned here in *Aven*$^{-/-}$ (but not WT), and SKIV2L binding increased (Figure S5B). This suggests that structure-prone regions impede translation in *Aven*$^{-/-}$, leading to ribosome stalling, RNA cleavage, SKIV2L recruitment, and decay. We speculate that AVEN helps suppress or melt RNA structures, consistent with its binding to structure-prone regions.

sORF Surveillance by AVEN and SKIV2L

As SKIV2L and AVEN specialize in translation surveillance, we did not expect them to bind non-coding RNAs (ncRNAs). However, upon *Aven* knockout, SKIV2L bound transcripts from intergenic, upstream, and antisense loci, and AVEN bound these ncRNAs in WT cells (see examples in Figure 7A). To examine this globally we divided the genome into 1 kb windows classified by protein-coding gene overlap, and calculated $\log_2$-fold changes in SKIV2L CRAC in *Aven*$^{-/-}$ versus WT (Table S6). This revealed increased SKIV2L binding to RNAs from hundreds of non-coding regions (Figure 7B), accompanied by increased U-tailing (Figure S5A). These transcripts were GC rich and predicted to form strong secondary structures (Figures S6A and S6B), suggesting the same mechanism drives SKIV2L recruitment to ncRNAs and mRNAs upon *Aven* deletion.

We wondered whether SKIV2L binding is due to ectopic ribosome occupancy on these "non-coding" RNAs. Indeed, monosomes and disomes accumulated at sites bound by SKIV2L in *Aven*$^{-/-}$ (e.g., Figure 7A), which often overlapped small ORFs (sORFs; Table S7), suggesting they are translated. Looking globally, we calculated $\log_2$-fold changes in monosome and disome counts for the non-coding 1-kb windows defined in Figure 7B, classified by differential SKIV2L binding in *Aven*$^{-/-}$. This revealed a correlation between gain of SKIV2L binding and increased monosome and disome occupancy (Figure 7C, "SKIV2L CRAC," "Monosomes", and "Disomes"). Elevated disome occupancy was particularly strong, suggesting increased ribosome stalling. The peptides generated by these translation events do not appear to perform conserved functions, as their sequences have low phyloCSF scores (Figure S6C). Changes in SKIV2L binding correlated with AVEN occupancy in WT cells, supporting a direct role for AVEN (Figure 7C, "AVEN vs SKIV2L CRAC"). Overall, our data suggest that loss of AVEN results in ribosome stalling on sORFs in structured ncRNAs, which is resolved by surveillance involving RNA cleavage and SKIV2L-dependent decay.

A prediction of this is that upon *Aven* deletion, these ncRNAs should become reliant on SKIV2L-dependent 3′–5′ decay, which specializes in degrading RNAs with arrested ribosomes. Presumably, alternative pathways remove these transcripts when AVEN is present. Indeed, these ncRNAs do not strongly accumulate in *Aven*$^{-/-}$ and only slowly accumulate upon *Skiv2l*

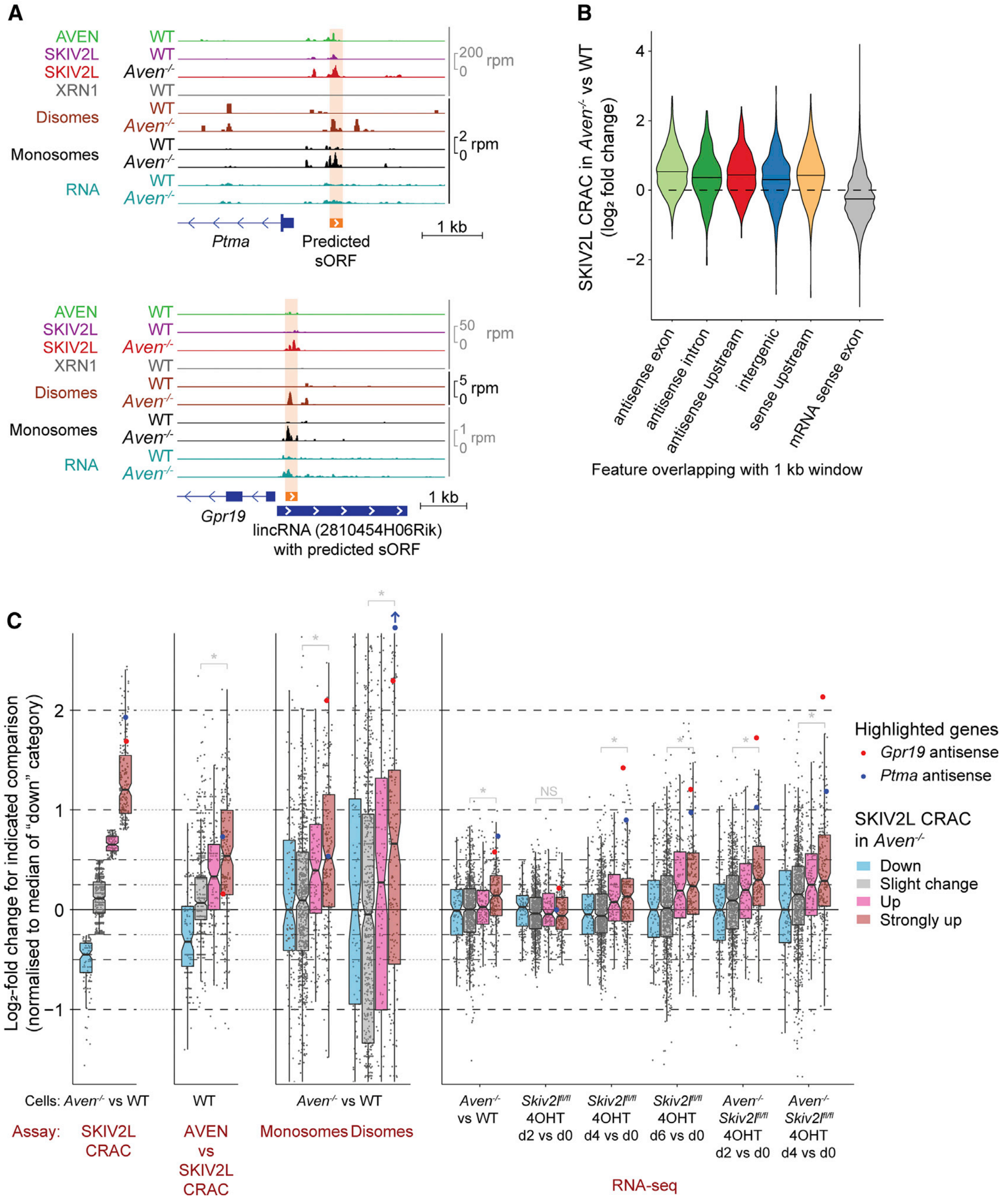

Figure 7. Small ORF Surveillance by AVEN and SKIV2L

(A) CRAC, monosome, and disome profiling across ncRNA regions in WT and *Aven*$^{-/-}$ cells, with small ORFs indicated. RNA-seq of ribosome profiling inputs shown in blue.

(B) SKIV2L CRAC changes (*Aven*$^{-/-}$ versus WT) for 1-kb genomic windows classified by overlap with protein-coding genes.

(C) CRAC, ribosome profiling, and RNA-seq changes for the indicated comparisons, for non-coding 1-kb genomic windows defined in (B). Windows are categorized by their change in SKIV2L CRAC for *Aven*$^{-/-}$ versus WT cells (defined in the leftmost plot). The two genes in (A) are highlighted. *$p < 10^{-4}$ ("Slight change" versus "Strongly up" categories; Mann-Whitney U test with Bonferroni correction).

See also Figure S6 and Tables S6 and S7.

knockout but rapidly accumulate when *Skiv2l* is knocked out in *Aven*$^{-/-}$ cells (Figure 7C, "RNA-seq"). We conclude that the absence of AVEN renders cells dependent on SKIV2L to clear ncRNAs with trapped ribosomes. The AVEN-SKIV2L pathway thus plays a universal role in counteracting aberrant translation on coding RNAs and ncRNAs.

DISCUSSION

Mammalian mRNA Decay: Specialization and Links to Translation

We are struck by the widespread coupling between cytoplasmic mRNA decay and translation revealed by our study. Evidence of such crosstalk has been mounting, from reports that SKIV2L and XRN1 associate with polysomes (Mangus and Jacobson, 1999; Qu et al., 1998) to analyses of decay intermediates (Antic et al., 2015; Hu et al., 2009; Pelechano et al., 2015) and structures of the Ski complex and Xrn1 bound to yeast ribosomes (Schmidt et al., 2016; Tesina et al., 2019). We show that XRN1 and SKIV2L ribosome binding sites are conserved to mammals, these interactions occur under physiological conditions, and remarkably, SKIV2L is exclusively and universally recruited by ribosomes.

Ski2 was thought to act redundantly with Xrn1 in bulk RNA decay, based on synthetic lethality in yeast (Anderson and Parker, 1998; Johnson and Kolodner, 1995). However, yeast Ski2 binding to 3′ UTRs (Sohrabi-Jahromi et al., 2019; Tuck and Tollervey, 2013) relies on fungus-specific factors such as Ska1 to antagonize ribosome interactions (Zhang et al., 2019). Our data argue that mammalian SKIV2L does not function in full-length mRNA decay but acts almost exclusively in translation-associated RNA surveillance. As the Ski complex is indispensable for cytoplasmic exosome activity (Anderson and Parker, 1998; Araki et al., 2001; van Hoof et al., 2000), this implies that the cytoplasmic exosome acts similarly exclusively in surveillance. We note that mammals possess an exosome-independent 3′–5′ decay pathway (DIS3L2). This might assist XRN1 in bulk decay, in line with a report that XRN1 and DIS3L2 knockdowns result in broader mRNA changes than DIS3L (exosome) knockdown (Lubas et al., 2013). The 3′ UTR accumulation of XRN1 suggests a passive role for translation in 5′–3′ decay. Future biochemical studies should help clarify these possible differences between SKIV2L- and XRN1-ribosome interactions.

Interestingly, we found that SKIV2L acts in bulk decay of a few mRNAs. Unique features might render these accessible to, or dependent on, ribosome-bound SKIV2L. For example, cleavage of *Ifrd1* might generate an access point for SKIV2L (Ottens et al., 2017), and ribosome-bound SKIV2L could reach the end of short histone mRNA 3′ UTRs. This was proposed for *S. cerevisiae* mRNAs (Zhang et al., 2019), and we see SKIV2L binding to very short 3′ UTRs (Figure 2B). Alternatively, surveillance-inducing ribosome collisions may be rife within histone mRNAs, whose decay requires stalled ribosome factors HBS1 and PELOTA (Slevin et al., 2014). Although this pathway is wasteful, as it eliminates the nascent polypeptide, for replication-dependent histones, this may help to tightly restrict their expression to S-phase.

For most mRNAs, however, there is a clear division of labor, with XRN1 specializing in bulk RNA decay (albeit with a minor role in surveillance) and SKIV2L in surveillance. This ensures that translation is not interrupted by bulk RNA turnover, as XRN1 follows the last ribosome, and may reflect a need for dedicated surveillance factors to wrestle mRNAs from arrested ribosomes. Indeed, it is even possible that SKIV2L could perform additional roles in resolving stalled mRNA-ribosome complexes, beyond assisting the exosome in 3′–5′ decay.

Defining Translation-Dependent mRNA Surveillance

Our data also reveal triggers and components of RNA surveillance. SKIV2L pervasively interacts with ribosome-occupied regions, establishing it as a central component of translation surveillance. Based on the low level of U-tailing (a proxy for RNA cleavage), we suggest SKIV2L binding in WT cells mostly reflects dynamic probing of translation, which rarely triggers a full surveillance response. Nonetheless, SKIV2L and disomes were enriched at A-rich tracts, proline sequences, and uORFs, suggesting they occasionally trigger ribosome stalling and RNA decay. For A-rich tracts, the sequence appears key, consistent with reports that ∼11 As attenuate translation in human cells (Arthur et al., 2015). We find this occurs at many endogenous sites with 8–9 As sufficient.

Besides defining SKIV2L targets, we established AVEN and FOCAD as components of this pathway. AVEN was reported to interact with the Ski complex and FOCAD in human cells (Thandapani et al., 2015) and identified in an NMD screen (Alexandrov et al., 2017), and the plant FOCAD homolog Rst1 interacts with the Ski complex and exosome (Lange et al., 2019). AVEN is conserved from mammals to flies (Zou et al., 2011) and FOCAD to plants (Lange et al., 2019), so their RNA decay roles may be evolutionarily important.

AVEN as an Anti-stalling Factor

We propose that AVEN prevents ribosome stalls, which otherwise trigger mRNA cleavage and decay. The RNA-binding preferences and position of AVEN on the ribosome might let it directly melt structures arresting translation, potentially via its RGG/RG domain. Supporting this, FUS and AUF1 RGG/RG domains remodel RNA (Loughlin et al., 2019; Meyer et al., 2019). Alternatively, AVEN might recruit a helicase (Thandapani et al., 2015), although besides SKIV2L, we did not detect helicase partners for AVEN.

In our model, AVEN acts prior to SKIV2L, to prevent ribosome stalling, and is potentially loaded with scanning ribosomes. However, our IP-MS data suggest that AVEN and SKIV2L directly interact. To resolve this paradox, we propose that the AVEN-SKIV2L interaction is transient, perhaps serving as a handover to ensure unresolved ribosome stalls are not left unchecked. Transient "connections" are common in RNA surveillance, as reported for Ski complex-exosome (Kalisiak et al., 2017) and nuclear MTR4-ZFC3H1-PABPN1 interactions (Meola et al., 2016).

Exploring the AVEN-SKIV2L pathway revealed that uORF-containing and histone mRNAs are particularly sensitive. AVEN prevents cell-cycle arrest in osteosarcoma and *Drosophila* cells (Baranski et al., 2015; Zou et al., 2011) and delays mitotic entry in *Xenopus* egg extracts (Guo et al., 2008; Zou et al., 2011). Our data suggest AVEN also plays a direct role in cell-cycle progression via reducing ribosome stalling on histone mRNAs. The most surprising AVEN and SKIV2L substrates, however, were ncRNAs. Here, an appealing model is that AVEN assists in functional small peptide production. Although AVEN-dependent sORFs have low phyloCSF scores and we

could not detect derived peptides, AVEN could enable cells to express peptides that eventually evolve to become stable and perform important roles. Alternatively, AVEN and SKIV2L may target nuclear ncRNAs escaping to the cytoplasm. These structured RNAs could function in the nucleus but in the cytoplasm might become stuck on ribosomes if left unchecked.

In conclusion, we find that mammalian RNA decay pathways are highly specialized and cytoplasmic decay is widely coupled to translation. While normal translation may assist bulk mRNA turnover, aberrant translation events pose a diverse threat counteracted by the concerted activity of AVEN and SKIV2L.

STAR★METHODS

Detailed methods are provided in the online version of this paper and include the following:

- KEY RESOURCES TABLE
- LEAD CONTACT AND MATERIALS AVAILABILITY
- EXPERIMENTAL MODEL AND SUBJECT DETAILS
- METHOD DETAILS
    - Generation of endogenously tagged cell lines
    - Generation of straight KO cell lines
    - Generation of conditional KO cell lines
    - Transfections
    - RNA sequencing
    - CRAC
    - Translation inhibition experiments for CRAC
    - Ribosome profiling
    - Western Blotting
    - Co-immunoprecipitations
    - Affinity purification for LC–MS/MS
    - Mass spectrometry analysis
    - Cell cycle analysis
    - ATF4 induction with Thapsigargin
    - RT-qPCR
- QUANTIFICATION AND STATISTICAL ANALYSIS
    - CRAC data preprocessing and alignment
    - CRAC quantification of non-templated 3′ tails
    - CRAC PCA, correlation matrix and tSNE
    - Differential SKIV2L binding analysis
    - Identification of rRNA binding sites by CRAC
    - CRAC plots around start and stop codons
    - CRAC enrichment at amino acid combinations
    - CRAC and disome profiling repeat analyis
    - RNA-seq analysis
    - RNA half-life analysis
    - CRAC sequence and structure motif analysis
    - CRAC/ribosome profiling at structured regions
    - CRAC/ribosome profiling for genomic windows
    - Ribosome profiling analysis
- DATA AND CODE AVAILABILITY

SUPPLEMENTAL INFORMATION

Supplemental Information can be found online at https://doi.org/10.1016/j.molcel.2020.01.007.

ACKNOWLEDGMENTS

We thank the FMI Functional Genomics, FACS and Protein Analysis facilities, and the University of Lausanne Genomics Technology Facility. This work was supported by the Swiss National Science Foundation (SNSF), NCCR RNA & Disease (grant 141735), and the Novartis Research Foundation. A.C.T. is supported by the Wellcome Trust (103977), and D.G. is supported by the SNSF (grant 179190) and the University of Lausanne.

AUTHOR CONTRIBUTIONS

A.C.T. and A.R. conceived the project, designed and performed experiments, analyzed data, and wrote the manuscript. A.C.T. performed bioinformatics analyses. M.B. and D.G. conceived the project, designed experiments, and edited the manuscript. A.B.A. and V.C.-S. analyzed ribosome profiling data. L.A.L. performed ribosome profiling experiments. D.H. and V.I. performed MS analyses.

DECLARATION OF INTERESTS

The Friedrich Miescher Institute for Biomedical Research (FMI) receives significant financial contributions from the Novartis Research Foundation.

Received: July 29, 2019
Revised: November 11, 2019
Accepted: January 7, 2020
Published: February 10, 2020

REFERENCES

Alexandrov, A., Shu, M.-D., and Steitz, J.A. (2017). Fluorescence amplification method for forward genetic discovery of factors in human mRNA degradation. Mol. Cell *65*, 191–201.

Anderson, J.S., and Parker, R.P. (1998). The 3′ to 5′ degradation of yeast mRNAs is a general mechanism for mRNA turnover that requires the SKI2 DEVH box protein and 3′ to 5′ exonucleases of the exosome complex. EMBO J. *17*, 1497–1506.

Antic, S., Wolfinger, M.T., Skucha, A., Hosiner, S., and Dorner, S. (2015). General and microRNA-mediated mRNA degradation occurs on ribosome complexes in Drosophila cells. Mol. Cell. Biol. *35*, 2309–2320.

Araki, Y., Takahashi, S., Kobayashi, T., Kajiho, H., Hoshino, S., and Katada, T. (2001). Ski7p G protein interacts with the exosome and the Ski complex for 3′-to-5′ mRNA decay in yeast. EMBO J. *20*, 4684–4693.

Arpat, A.B., Liechti, A., Matos, M.D., Dreos, R., Janich, P., and Gatfield, D. (2019). Transcriptome-wide sites of collided ribosomes reveal principles of translational pausing. bioRxiv. https://doi.org/10.1101/710061.

Arthur, L., Pavlovic-Djuranovic, S., Smith-Koutmou, K., Green, R., Szczesny, P., and Djuranovic, S. (2015). Translational control by lysine-encoding A-rich sequences. Sci. Adv. *1*, e1500154.

Baranski, Z., Booij, T.H., Cleton-Jansen, A.M., Price, L.S., van de Water, B., Bovée, J.V., Hogendoorn, P.C., and Danen, E.H. (2015). Aven-mediated checkpoint kinase control regulates proliferation and resistance to chemotherapy in conventional osteosarcoma. J. Pathol. *236*, 348–359.

Bresson, S., Tuck, A., Staneva, D., and Tollervey, D. (2017). Nuclear RNA decay pathways aid rapid remodeling of gene expression in yeast. Mol. Cell *65*, 787–800.

Brockschmidt, A., Trost, D., Peterziel, H., Zimmermann, K., Ehrler, M., Grassmann, H., Pfenning, P.N., Waha, A., Wohlleber, D., Brockschmidt, F.F., et al. (2012). KIAA1797/FOCAD encodes a novel focal adhesion protein with tumour suppressor function in gliomas. Brain *135*, 1027–1041.

Calvo, S.E., Pagliarini, D.J., and Mootha, V.K. (2009). Upstream open reading frames cause widespread reduction of protein expression and are polymorphic among humans. Proc. Natl. Acad. Sci. USA *106*, 7507–7512.

Castelo-Szekely, V., De Matos, M., Tusup, M., Pascolo, S., Ule, J., and Gatfield, D. (2019). Charting DENR-dependent translation reinitiation uncovers

predictive uORF features and links to circadian timekeeping via Clock. Nucleic Acids Res. *47*, 5193–5209.

Chothani, S.P., Adami, E., Viswanathan, S., Hubner, N., Cook, S., Schafer, S., and Rackham, O.J.L. (2017). Reliable detection of translational regulation with Ribo-seq. bioRxiv. https://doi.org/10.1101/234344.

Cox, J., Neuhauser, N., Michalski, A., Scheltema, R.A., Olsen, J.V., and Mann, M. (2011). Andromeda: a peptide search engine integrated into the MaxQuant environment. J. Proteome Res. *10*, 1794–1805.

D'Orazio, K.N., Wu, C.C.-C., Sinha, N., Loll-Krippleber, R., Brown, G.W., and Green, R. (2019). The endonuclease Cue2 cleaves mRNAs at stalled ribosomes during No Go Decay. eLife *8*, e49117.

Dobin, A., Davis, C.A., Schlesinger, F., Drenkow, J., Zaleski, C., Jha, S., Batut, P., Chaisson, M., and Gingeras, T.R. (2013). STAR: ultrafast universal RNA-seq aligner. Bioinformatics *29*, 15–21.

Eißmann, M., Melzer, I.M., Fernández, S.B.M., Michel, G., Hrabě de Angelis, M., Hoefler, G., Finkenwirth, P., Jauch, A., Schoell, B., Grez, M., et al. (2013). Overexpression of the anti-apoptotic protein AVEN contributes to increased malignancy in hematopoietic neoplasms. Oncogene *32*, 2586–2591.

Fabre, A., Charroux, B., Martinez-Vinson, C., Roquelaure, B., Odul, E., Sayar, E., Smith, H., Colomb, V., Andre, N., Hugot, J.-P., et al. (2012). SKIV2L mutations cause syndromic diarrhea, or trichohepatoenteric syndrome. Am. J. Hum. Genet. *90*, 689–692.

Flemr, M., and Bühler, M. (2015). Single-step generation of conditional knockout mouse embryonic stem cells. Cell Rep. *12*, 709–716.

Flicek, P., Ahmed, I., Amode, M.R., Barrell, D., Beal, K., Brent, S., Carvalho-Silva, D., Clapham, P., Coates, G., Fairley, S., et al. (2013). Ensembl 2013. Nucleic Acids Res. *41*, D48–D55.

Gatfield, D., and Izaurralde, E. (2004). Nonsense-mediated messenger RNA decay is initiated by endonucleolytic cleavage in Drosophila. Nature *429*, 575–578.

Ghosh, S., and Jacobson, A. (2010). RNA decay modulates gene expression and controls its fidelity. Wiley Interdiscip. Rev. RNA *1*, 351–361.

Glover, M.L., Burroughs, A.M., Egelhofer, T.A., Pule, M.N., Aravind, L., and Arribere, J.A. (2019). NONU-1 encodes a conserved endonuclease required for mRNA translation surveillance. bioRxiv. https://doi.org/10.1101/674358.

Granneman, S., Kudla, G., Petfalski, E., and Tollervey, D. (2009). Identification of protein binding sites on U3 snoRNA and pre-rRNA by UV cross-linking and high-throughput analysis of cDNAs. Proc. Natl. Acad. Sci. USA *106*, 9613–9618.

Grozdanov, P., Georgiev, O., and Karagyozov, L. (2003). Complete sequence of the 45-kb mouse ribosomal DNA repeat: analysis of the intergenic spacer. Genomics *82*, 637–643.

Guo, J.Y., Yamada, A., Kajino, T., Wu, J.Q., Tang, W., Freel, C.D., Feng, J., Chau, B.N., Wang, M.Z., Margolis, S.S., et al. (2008). Aven-dependent activation of ATM following DNA damage. Curr. Biol. *18*, 933–942.

Guydosh, N.R., and Green, R. (2014). Dom34 rescues ribosomes in 3′ untranslated regions. Cell *156*, 950–962.

Guydosh, N.R., and Green, R. (2017). Translation of poly(A) tails leads to precise mRNA cleavage. RNA *23*, 749–761.

Halbach, F., Reichelt, P., Rode, M., and Conti, E. (2013). The yeast ski complex: crystal structure and RNA channeling to the exosome complex. Cell *154*, 814–826.

Harding, H.P., Novoa, I., Zhang, Y., Zeng, H., Wek, R., Schapira, M., and Ron, D. (2000). Regulated translation initiation controls stress-induced gene expression in mammalian cells. Mol. Cell *6*, 1099–1108.

Hartley, J.L., Zachos, N.C., Dawood, B., Donowitz, M., Forman, J., Pollitt, R.J., Morgan, N.V., Tee, L., Gissen, P., Kahr, W.H., et al. (2010). Mutations in TTC37 cause trichohepatoenteric syndrome (phenotypic diarrhea of infancy). Gastroenterology *138*, 2388–2398, 2398.e1–2398.e2.

Hsu, C.L., and Stevens, A. (1993). Yeast cells lacking 5′–>3′ exoribonuclease 1 contain mRNA species that are poly(A) deficient and partially lack the 5′ cap structure. Mol. Cell. Biol. *13*, 4826–4835.

Hu, W., Sweet, T.J., Chamnongpol, S., Baker, K.E., and Coller, J. (2009). Co-translational mRNA decay in Saccharomyces cerevisiae. Nature *461*, 225–229.

Ibrahim, F., Maragkakis, M., Alexiou, P., and Mourelatos, Z. (2018). Ribothrypsis, a novel process of canonical mRNA decay, mediates ribosome-phased mRNA endonucleolysis. Nat. Struct. Mol. Biol. *25*, 302–310.

Ikeuchi, K., Tesina, P., Matsuo, Y., Sugiyama, T., Cheng, J., Saeki, Y., Tanaka, K., Becker, T., Beckmann, R., and Inada, T. (2019). Collided ribosomes form a unique structural interface to induce Hel2-driven quality control pathways. EMBO J. *38*, e100276.

Ingolia, N.T., Lareau, L.F., and Weissman, J.S. (2011). Ribosome profiling of mouse embryonic stem cells reveals the complexity and dynamics of mammalian proteomes. Cell *147*, 789–802.

Janich, P., Arpat, A.B., Castelo-Szekely, V., Lopes, M., and Gatfield, D. (2015). Ribosome profiling reveals the rhythmic liver translatome and circadian clock regulation by upstream open reading frames. Genome Res. *25*, 1848–1859.

Johnson, A.W., and Kolodner, R.D. (1995). Synthetic lethality of sep1 (xrn1) ski2 and sep1 (xrn1) ski3 mutants of Saccharomyces cerevisiae is independent of killer virus and suggests a general role for these genes in translation control. Mol. Cell. Biol. *15*, 2719–2727.

Juszkiewicz, S., Chandrasekaran, V., Lin, Z., Kraatz, S., Ramakrishnan, V., and Hegde, R.S. (2018). ZNF598 is a quality control sensor of collided ribosomes. Mol. Cell *72*, 469–481.

Kalisiak, K., Kuliński, T.M., Tomecki, R., Cysewski, D., Pietras, Z., Chlebowski, A., Kowalska, K., and Dziembowski, A. (2017). A short splicing isoform of HBS1L links the cytoplasmic exosome and SKI complexes in humans. Nucleic Acids Res. *45*, 2068–2080.

Kearse, M.G., Goldman, D.H., Choi, J., Nwaezeapu, C., Liang, D., Green, K.M., Goldstrohm, A.C., Todd, P.K., Green, R., and Wilusz, J.E. (2019). Ribosome queuing enables non-AUG translation to be resistant to multiple protein synthesis inhibitors. Genes Dev. *33*, 871–885.

Khatter, H., Myasnikov, A.G., Natchiar, S.K., and Klaholz, B.P. (2015). Structure of the human 80S ribosome. Nature *520*, 640–645.

Kilchert, C., Wittmann, S., and Vasiljeva, L. (2016). The regulation and functions of the nuclear RNA exosome complex. Nat. Rev. Mol. Cell Biol. *17*, 227–239.

Knuckles, P., Carl, S.H., Musheev, M., Niehrs, C., Wenger, A., and Bühler, M. (2017). RNA fate determination through cotranscriptional adenosine methylation and microprocessor binding. Nat. Struct. Mol. Biol. *24*, 561–569.

Łabno, A., Tomecki, R., and Dziembowski, A. (2016). Cytoplasmic RNA decay pathways: enzymes and mechanisms. Biochim. Biophys. Acta *1863*, 3125–3147.

LaCava, J., Houseley, J., Saveanu, C., Petfalski, E., Thompson, E., Jacquier, A., and Tollervey, D. (2005). RNA degradation by the exosome is promoted by a nuclear polyadenylation complex. Cell *121*, 713–724.

Lange, H., Ndecky, S.Y.A., Gomez-Diaz, C., Pflieger, D., Butel, N., Zumsteg, J., Kuhn, L., Piermaria, C., Chicher, J., Christie, M., et al. (2019). RST1 and RIPR connect the cytosolic RNA exosome to the Ski complex in Arabidopsis. Nat. Commun. *10*, 3871.

Langmead, B., and Salzberg, S.L. (2012). Fast gapped-read alignment with Bowtie 2. Nat. Methods *9*, 357–359.

Li, H., Handsaker, B., Wysoker, A., Fennell, T., Ruan, J., Homer, N., Marth, G., Abecasis, G., and Durbin, R.; 1000 Genome Project Data Processing Subgroup (2009). The Sequence Alignment/Map format and SAMtools. Bioinformatics *25*, 2078–2079.

Lim, J., Ha, M., Chang, H., Kwon, S.C., Simanshu, D.K., Patel, D.J., and Kim, V.N. (2014). Uridylation by TUT4 and TUT7 marks mRNA for degradation. Cell *159*, 1365–1376.

Lorenz, R., Bernhart, S.H., Höner Zu Siederdissen, C., Tafer, H., Flamm, C., Stadler, P.F., and Hofacker, I.L. (2011). ViennaRNA Package 2.0. Algorithms Mol. Biol. *6*, 26.

Loughlin, F.E., Lukavsky, P.J., Kazeeva, T., Reber, S., Hock, E.-M., Colombo, M., Von Schroetter, C., Pauli, P., Cléry, A., Mühlemann, O., et al. (2019). The

solution structure of FUS bound to RNA reveals a bipartite mode of RNA recognition with both sequence and shape specificity. Mol. Cell *73*, 490–504.e6.

Love, M.I., Huber, W., and Anders, S. (2014). Moderated estimation of fold change and dispersion for RNA-seq data with DESeq2. Genome Biol. *15*, 550.

Lubas, M., Christensen, M.S., Kristiansen, M.S., Domanski, M., Falkenby, L.G., Lykke-Andersen, S., Andersen, J.S., Dziembowski, A., and Jensen, T.H. (2011). Interaction profiling identifies the human nuclear exosome targeting complex. Mol. Cell *43*, 624–637.

Lubas, M., Damgaard, C.K., Tomecki, R., Cysewski, D., Jensen, T.H., and Dziembowski, A. (2013). Exonuclease hDIS3L2 specifies an exosome-independent 3′-5′ degradation pathway of human cytoplasmic mRNA. EMBO J. *32*, 1855–1868.

Mangus, D.A., and Jacobson, A. (1999). Linking mRNA turnover and translation: assessing the polyribosomal association of mRNA decay factors and degradative intermediates. Methods *17*, 28–37.

Martin, M. (2011). Cutadapt removes adapter sequences from high-throughput sequencing reads. EMBnet.journal *17*, 10–12.

Meola, N., Domanski, M., Karadoulama, E., Chen, Y., Gentil, C., Pultz, D., Vitting-Seerup, K., Lykke-Andersen, S., Andersen, J.S., Sandelin, A., and Jensen, T.H. (2016). Identification of a nuclear exosome decay pathway for processed transcripts. Mol. Cell *64*, 520–533.

Meyer, A., Golbik, R.P., Sänger, L., Schmidt, T., Behrens, S.E., and Friedrich, S. (2019). The RGG/RG motif of AUF1 isoform p45 is a key modulator of the protein's RNA chaperone and RNA annealing activities. RNA Biol. *16*, 960–971.

Mitchell, P., Petfalski, E., Shevchenko, A., Mann, M., and Tollervey, D. (1997). The exosome: a conserved eukaryotic RNA processing complex containing multiple 3′–>5′ exoribonucleases. Cell *91*, 457–466.

Mohn, F., Weber, M., Rebhan, M., Roloff, T.C., Richter, J., Stadler, M.B., Bibel, M., and Schübeler, D. (2008). Lineage-specific polycomb targets and de novo DNA methylation define restriction and potential of neuronal progenitors. Mol. Cell *30*, 755–766.

Morton, D.J., Kuiper, E.G., Jones, S.K., Leung, S.W., Corbett, A.H., and Fasken, M.B. (2018). The RNA exosome and RNA exosome-linked disease. RNA *24*, 127–142.

Mullen, T.E., and Marzluff, W.F. (2008). Degradation of histone mRNA requires oligouridylation followed by decapping and simultaneous degradation of the mRNA both 5′ to 3′ and 3′ to 5′. Genes Dev. *22*, 50–65.

Ostapcuk, V., Mohn, F., Carl, S.H., Basters, A., Hess, D., Iesmantavicius, V., Lampersberger, L., Flemr, M., Pandey, A., Thomä, N.H., et al. (2018). Activity-dependent neuroprotective protein recruits HP1 and CHD4 to control lineage-specifying genes. Nature *557*, 739–743.

Ottens, F., Boehm, V., Sibley, C.R., Ule, J., and Gehring, N.H. (2017). Transcript-specific characteristics determine the contribution of endo- and exonucleolytic decay pathways during the degradation of nonsense-mediated decay substrates. RNA *23*, 1224–1236.

Parker, R. (2012). RNA degradation in Saccharomyces cerevisae. Genetics *191*, 671–702.

Pashler, A.L., Towler, B.P., Jones, C.I., and Newbury, S.F. (2016). The roles of the exoribonucleases DIS3L2 and XRN1 in human disease. Biochem. Soc. Trans. *44*, 1377–1384.

Pavlov, M.Y., Watts, R.E., Tan, Z., Cornish, V.W., Ehrenberg, M., and Forster, A.C. (2009). Slow peptide bond formation by proline and other N-alkylamino acids in translation. Proc. Natl. Acad. Sci. USA *106*, 50–54.

Pelechano, V., Wei, W., and Steinmetz, L.M. (2015). Widespread co-translational RNA decay reveals ribosome dynamics. Cell *161*, 1400–1412.

Pérez-Ortín, J.E., Alepuz, P., Chávez, S., and Choder, M. (2013). Eukaryotic mRNA decay: methodologies, pathways, and links to other stages of gene expression. J. Mol. Biol. *425*, 3750–3775.

Pertea, M., Pertea, G.M., Antonescu, C.M., Chang, T.C., Mendell, J.T., and Salzberg, S.L. (2015). StringTie enables improved reconstruction of a transcriptome from RNA-seq reads. Nat. Biotechnol. *33*, 290–295.

Qu, X., Yang, Z., Zhang, S., Shen, L., Dangel, A.W., Hughes, J.H., Redman, K.L., Wu, L.-C., and Yu, C.Y. (1998). The human DEVH-box protein Ski2w from the HLA is localized in nucleoli and ribosomes. Nucleic Acids Res. *26*, 4068–4077.

Quinlan, A.R. (2014). BEDTools: the Swiss-army tool for genome feature analysis. Curr. Protoc. Bioinformatics *47*, 1–34.

Robinson, M.D., McCarthy, D.J., and Smyth, G.K. (2010). edgeR: a Bioconductor package for differential expression analysis of digital gene expression data. Bioinformatics *26*, 139–140.

Robinson, S.R., Oliver, A.W., Chevassut, T.J., and Newbury, S.F. (2015). The 3′ to 5′ exoribonuclease DIS3: from structure and mechanisms to biological functions and role in human disease. Biomolecules *5*, 1515–1539.

Roy, B., and Jacobson, A. (2013). The intimate relationships of mRNA decay and translation. Trends Genet. *29*, 691–699.

Schmid, M., and Jensen, T.H. (2018). Controlling nuclear RNA levels. Nat. Rev. Genet. *19*, 518–529.

Schmidt, C., Kowalinski, E., Shanmuganathan, V., Defenouillère, Q., Braunger, K., Heuer, A., Pech, M., Namane, A., Berninghausen, O., Fromont-Racine, M., et al. (2016). The cryo-EM structure of a ribosome-Ski2-Ski3-Ski8 helicase complex. Science *354*, 1431–1433.

Schmieder, R., and Edwards, R. (2011). Quality control and preprocessing of metagenomic datasets. Bioinformatics *27*, 863–864.

Simms, C.L., Yan, L.L., and Zaher, H.S. (2017). Ribosome collision is critical for quality control during no-go decay. Mol. Cell *68*, 361–373.

Slevin, M.K., Meaux, S., Welch, J.D., Bigler, R., Miliani de Marval, P.L., Su, W., Rhoads, R.E., Prins, J.F., and Marzluff, W.F. (2014). Deep sequencing shows multiple oligouridylations are required for 3′ to 5′ degradation of histone mRNAs on polyribosomes. Mol. Cell *53*, 1020–1030.

Sohrabi-Jahromi, S., Hofmann, K.B., Boltendahl, A., Roth, C., Gressel, S., Baejen, C., Soeding, J., and Cramer, P. (2019). Transcriptome maps of general eukaryotic RNA degradation factors. eLife *8*, e47040.

Team, R.C. (2013). R: A Language and Environment for Statistical Computing (R Foundation for Statistical Computing).

Tesina, P., Heckel, E., Cheng, J., Fromont-Racine, M., Buschauer, R., Kater, L., Beatrix, B., Berninghausen, O., Jacquier, A., Becker, T., and Beckmann, R. (2019). Structure of the 80S ribosome-Xrn1 nuclease complex. Nat. Struct. Mol. Biol. *26*, 275–280.

Thandapani, P., Song, J., Gandin, V., Cai, Y., Rouleau, S.G., Garant, J.-M., Boisvert, F.-M., Yu, Z., Perreault, J.-P., Topisirovic, I., and Richard, S. (2015). Aven recognition of RNA G-quadruplexes regulates translation of the mixed lineage leukemia protooncogenes. eLife *4*, e06234.

Travis, A.J., Moody, J., Helwak, A., Tollervey, D., and Kudla, G. (2014). Hyb: a bioinformatics pipeline for the analysis of CLASH (crosslinking, ligation and sequencing of hybrids) data. Methods *65*, 263–273.

Tuck, A.C., and Tollervey, D. (2013). A transcriptome-wide atlas of RNP composition reveals diverse classes of mRNAs and lncRNAs. Cell *154*, 996–1009.

Tuck, A.C., Natarajan, K.N., Rice, G.M., Borawski, J., Mohn, F., Rankova, A., Flemr, M., Wenger, A., Nutiu, R., Teichmann, S., and Bühler, M. (2018). Distinctive features of lincRNA gene expression suggest widespread RNA-independent functions. Life Sci. Alliance *1*, e201800124.

Tyanova, S., Temu, T., Sinitcyn, P., Carlson, A., Hein, M.Y., Geiger, T., Mann, M., and Cox, J. (2016). The Perseus computational platform for comprehensive analysis of (prote)omics data. Nat. Methods *13*, 731–740.

van Hoof, A., Staples, R.R., Baker, R.E., and Parker, R. (2000). Function of the ski4p (Csl4p) and Ski7p proteins in 3′-to-5′ degradation of mRNA. Mol. Cell. Biol. *20*, 8230–8243.

Vattem, K.M., and Wek, R.C. (2004). Reinitiation involving upstream ORFs regulates ATF4 mRNA translation in mammalian cells. Proc. Natl. Acad. Sci. USA *101*, 11269–11274.

Webb, S., Hector, R.D., Kudla, G., and Granneman, S. (2014). PAR-CLIP data indicate that Nrd1-Nab3-dependent transcription termination regulates expression of hundreds of protein coding genes in yeast. Genome Biol. *15*, R8.

Welte, T., Tuck, A.C., Papasaikas, P., Carl, S.H., Flemr, M., Knuckles, P., Rankova, A., Bühler, M., and Großhans, H. (2019). The RNA hairpin binder TRIM71 modulates alternative splicing by repressing MBNL1. Genes Dev. *33*, 1221–1235.

Weren, R.D., Venkatachalam, R., Cazier, J.B., Farin, H.F., Kets, C.M., de Voer, R.M., Vreede, L., Verwiel, E.T., van Asseldonk, M., Kamping, E.J., et al. (2015). Germline deletions in the tumour suppressor gene FOCAD are associated with polyposis and colorectal cancer development. J. Pathol. *236*, 155–164.

Wickham, H. (2016). ggplot2: Elegant Graphics for Data Analysis (New York: Springer-Verlag).

Widner, W.R., and Wickner, R.B. (1993). Evidence that the SKI antiviral system of Saccharomyces cerevisiae acts by blocking expression of viral mRNA. Mol. Cell. Biol. *13*, 4331–4341.

Zhang, E., Khanna, V., Dacheux, E., Namane, A., Doyen, A., Gomard, M., Turcotte, B., Jacquier, A., and Fromont-Racine, M. (2019). A specialised SKI complex assists the cytoplasmic RNA exosome in the absence of direct association with ribosomes. EMBO J. *38*, e100640.

Zhao, C., Datta, S., Mandal, P., Xu, S., and Hamilton, T. (2010). Stress-sensitive regulation of IFRD1 mRNA decay is mediated by an upstream open reading frame. J. Biol. Chem. *285*, 8552–8562.

Zinder, J.C., and Lima, C.D. (2017). Targeting RNA for processing or destruction by the eukaryotic RNA exosome and its cofactors. Genes Dev. *31*, 88–100.

Zou, S., Chang, J., LaFever, L., Tang, W., Johnson, E.L., Hu, J., Wilk, R., Krause, H.M., Drummond-Barbosa, D., and Irusta, P.M. (2011). Identification of dAven, a Drosophila melanogaster ortholog of the cell cycle regulator Aven. Cell Cycle *10*, 989–998.

STAR★METHODS

KEY RESOURCES TABLE

REAGENT or RESOURCE	SOURCE	IDENTIFIER
Antibodies		
Mouse anti-FLAG M2	Sigma	Cat#F1804
Anti-FLAG M2 Dynabeads	Sigma	Cat#M8823
Dynabeads M280 streptavidin-coated beads	Thermo Fisher	Cat#11206D
Dynabeads Protein G	Thermo Fisher	Cat# 10004D
Streptavidin-HRP	Sigma	Cat#S2438
Rabbit anti-AVEN	ProScience	Cat#2417
Rabbit anti-MTR4	ThermoFisher Scientific	Cat#PA557927
Rabbit anti-ATF4 D4B8	Cell Signaling	Cat#mAb11815
Rabbit anti-Phospho-eIF2α Ser51 D9G8	Cell Signaling	Cat#mAb3398
Rabbit anti-eIF4E	Bethyl Laboratories	Cat#A301-154A
Rat anti-tubulin clone YL1/2	Abcam	Cat#ab6160
Rat anti-HA	Roche	Cat#11867423001
Chemicals, Peptides, and Recombinant Proteins		
T4 DNA Ligase	Sigma	Cat#10716359001
DMEM	GIBCO	Cat#21969-035
Non-essential amino acids	GIBCO	Cat#11140035
100 mM Sodium pyruvate	GIBCO	Cat#11360070
200 mM L-glutamine	GIBCO	Cat#25030024
Fetal bovine serum	GIBCO	Cat#10270106
Beta-mercaptoethanol	Sigma	Cat#M-7522
Gelatin	Sigma	Cat#G-1890
Trypsin-EDTA	GIBCO	Cat#25300-054
Dulbecco's PBS	GIBCO	Cat#14190
Trypsin (TPCK-treated)	Sigma	Cat#T8802
OptiMEM	GIBCO	Cat#31985070
Lipofectamine 3000 Transfection kit	Invitrogen	Cat#L3000015
cOmplete Protease Inhibitor Cocktail	Roche	Cat#11836145001
Proteinase K	Roche	Cat#3115879001
SuperScript III	Life Technologies	Cat#18080085
3xFLAG peptide	Sigma	Cat#F4799-25MG
RNace-It Ribonuclease Cocktail	Agilent	Cat#400720
TSAP Thermosensitive Alkaline Phosphatase	Promega	Cat#M9910
RNasin Ribonuclease Inhibitor	Promega	Cat#N2115
Recombinant RNasin Ribonuclease Inhibitor	Promega	Cat#N2511
miR-cat 33 conversion oligo pack	IDT	N/A
T4 RNA Ligase 1 (ssRNA Ligase)	NEB	Cat#M0204L
T4 PNK, T4 polynucleotide kinase	NEB	Cat#M0201L
Hybond-C Extra membrane	GE Healthcare	Cat#RPN303E
Kodak BioMax MS autoradiography film	Kodak	Cat#8222648
MetaPhor agarose	Lonza	Cat#50180
NuPAGE 4–12% (wt/vol) polyacrylamide Bis-Tris gels	Life Technologies	Cat#NP0335
NuPAGE LDS sample buffer 4 ×	Life Technologies	Cat#NP0007
NuPAGE SDS-MOPS running buffer	Life Technologies	Cat#NP0001

(*Continued on next page*)

Continued		
REAGENT or RESOURCE	SOURCE	IDENTIFIER
NuPage transfer buffer	Life Technologies	Cat#NP00061
MinElute Gel extraction kit	QIAGEN	Cat#28604
Proteinase K	Roche	Cat#03115836001
RNase H	NEB	Cat#M0297L
TaKaRa long and accurate (LA) Taq	Clontech	Cat#RR002M
γ32P-ATP 0.5 mCi 18.5 MBq Spec act. > 6000 Ci/mmol	Hartman	Cat#SRP-501
NEBNext® High-Fidelity 2X PCR Master Mix	NEB	Cat#M0541
4-hydroxytamoxifen	Sigma	Cat#H6278
Puromycin	Sigma	Cat#P8833
Cycloheximide	Sigma	Cat#C7698
Harringtonine	LKT Laboratories	Cat#H0169
Immobilon Western Chemiluminiscent HRP Substrate	Merck Millipore	Cat#WBKLS0500
Thapsigargin	Invitrogen	Cat#T7459
Critical Commercial Assays		
ScriptSeq RNA-Seq Library Prep Kit	NEB	Cat#E7645
Agilent Absolutely RNA Miniprep Kit	Epicenter	Cat#SSV21106
TruSeq RNA Library Prep Kit v2	Illumina	Cat#RS-122-2001
miRNeasy RNA Extraction kit	QIAGEN	Cat#217004
Ribo-Zero Gold rRNA Removal Kit	Illumina	Cat#MRZG12324
PrimeScript RT-PCR Kit	Takara Bio	Cat#RR036A-1
Qubit dsDNA HS Assay Kit	Thermo Fisher	Cat#Q32854
SsoAdvanced SYBR Green Supermix	Bio-Rad	Cat#172-5274
Deposited Data		
CRAC	This paper	GEO: GSE134020
Ribosome profiling (monosome and disome profiling)	This paper	GEO: GSE134020
RNA-seq	This paper	GEO: GSE134020
Human ribosome structure	PDB	PDB #4UG0
Mouse pre-rRNA sequence	Grozdanov et al., 2003	GenBank BK000964
Mus musculus GRCm38/mm10 genome assembly, Mus_musculus.GRCm38.75.dna.primary_assembly.fa	Ensembl	ftp://ftp.ensembl.org/pub/release-75/fasta/mus_musculus/dna/
Gene annotations: Gencode M16 = Ensembl 91 (GRCm38) (including tRNAs and Appris isoforms)	Gencode	https://www.gencodegenes.org/mouse/release_M16.html
Original uncropped western blot images	This study	N/A
Experimental Models: Cell Lines		
Rosa26$^{Cre\text{-}ERT2/-}$	Flemr and Bühler, 2015	cMB052
Rosa26$^{Cre\text{-}ERT2/BirA\text{-}V5}$	Ostapcuk et al., 2018	cMB063
Rosa26$^{Cre\text{-}ERT2/BirA\text{-}V5}$ *Xrn1*$^{3xFLAG\text{-}Avi/3xFLAG\text{-}Avi}$	This study	cMB315
Rosa26$^{Cre\text{-}ERT2/BirA\text{-}V5}$ *Aven*$^{3xFLAG\text{-}Avi/3xFLAG\text{-}Avi}$	This study	cMB323
Rosa26$^{Cre\text{-}ERT2/BirA\text{-}V5}$ *Skiv2l*$^{3xFLAG\text{-}Avi/3xFLAG\text{-}Avi}$	This study	cMB331
Rosa26$^{Cre\text{-}ERT2/BirA\text{-}V5}$ *Mtr4*$^{1xFlag\text{-}Avi/1xFlag\text{-}Avi}$	Tuck et al., 2018	cMB376
Rosa26$^{Cre\text{-}ERT2/BirA\text{-}V5}$ *Rps10*$^{3xFLAG\text{-}Avi/3xFLAG\text{-}Avi}$	This study	cMB395
Rosa26$^{Cre\text{-}ERT2/BirA\text{-}V5}$ *Skiv2l*$^{3xFLAG\text{-}Avi/3xFLAG\text{-}Avi}$*Focad*$^{-/-}$	This study	cMB396
Rosa26$^{Cre\text{-}ERT2/BirA\text{-}V5}$ *Skiv2l*$^{3xFLAG\text{-}Avi/3xFLAG\text{-}Avi}$*Focad*$^{-/-}$	This study	cMB397
Rosa26$^{Cre\text{-}ERT2/BirA\text{-}V5}$ *Skiv2l*$^{3xFLAG\text{-}Avi/3xFLAG\text{-}Avi}$*Aven*$^{-/-}$	This study	cMB399
Rosa26$^{Cre\text{-}ERT2/BirA\text{-}V5}$ *Skiv2l*$^{3xFLAG\text{-}Avi/3xFLAG\text{-}Avi}$*Aven*$^{-/-}$	This study	cMB400
Rosa26$^{Cre\text{-}ERT2/-}$ *Skiv2l*$^{fl/fl}$	This study	cMB434
Rosa26$^{Cre\text{-}ERT2/-}$ *Skiv2l*$^{fl/fl}$	This study	cMB435

(*Continued on next page*)

REAGENT or RESOURCE	SOURCE	IDENTIFIER
Continued		
Rosa26$^{Cre-ERT2/BirA-V5}$ *Skiv2l*$^{3xFLAG-Avi/3xFLAG-Avi}$ *Aven*$^{-/-}$ *Skiv2l*$^{fl/fl}$	This study	cMB471
Rosa26$^{Cre-ERT2/BirA-V5}$ *Skiv2l*$^{3xFLAG-Avi/3xFLAG-Avi}$ *Aven*$^{-/-}$ *Skiv2l*$^{fl/fl}$	This study	cMB472
Rosa26$^{Cre-ERT2/BirA-V5}$ *Mtr4*$^{3xFlag-Avi/3xFlag-Avi}$	Tuck et al., 2018	cMB503
Rosa26$^{Cre-ERT2/BirA-V5}$ *Skiv2l*$^{3xFLAG-Avi/3xFLAG-Avi}$ *Dis3l*$^{2xHA-FKBP12(F36V)/\ 2xHA-FKBP12(F36V)}$	This study	cMB510
Oligonucleotides		
qPCR primers, see Table S1	This paper	N/A
Donor oligonucleotides for genome editing, see Table S1	This paper	N/A
5′ adapters for CRAC (barcodes highlighted):		N/A
/5InvddT/ACACrGrArCrGrCrUrCrUrUrCrCrGr ArUrCrUrNrNrNrNrUrArArGrC	L5Aa	IDT custom synthesis
/5InvddT/ACACrGrArCrGrCrUrCrUrUrCrCr GrArUrCrUrNrNrNrNrArUrUrArGrC	L5Ab	IDT custom synthesis
/5InvddT/ACACrGrArCrGrCrUrCrUrUrCrCrGr ArUrCrUrNrNrNrNrGrCrGrCrArGrC	L5Ac	IDT custom synthesis
/5InvddT/ACACrGrArCrGrCrUrCrUrUrCrCrGr ArUrCrUrNrNrNrNrCrGrCrUrUrArGrC	L5Ad	IDT custom synthesis
/5InvddT/ACACrGrArCrGrCrUrCrUrUrCrCrGr ArUrCrUrNrNrNrNrArGrArGrC	L5Ba	IDT custom synthesis
/5InvddT/ACACrGrArCrGrCrUrCrUrUrCrCrGr ArUrCrUrNrNrNrNrGrUrGrArGrC	L5Bb	IDT custom synthesis
/5InvddT/ACACrGrArCrGrCrUrCrUrUrCrCrGr ArUrCrUrNrNrNrNrCrArCrUrArGrC	L5Bc	IDT custom synthesis
/5InvddT/ACACrGrArCrGrCrUrCrUrUrCrCrGr ArUrCrUrNrNrNrNrUrCrUrCrUrArGrC	L5Bd	IDT custom synthesis
/5InvddT/ACACrGrArCrGrCrUrCrUrUrCrCrGr ArUrCrUrNrNrNrNrCrUrArGrC	L5Ca	IDT custom synthesis
/5InvddT/ACACrGrArCrGrCrUrCrUrUrCrCrGr ArUrCrUrNrNrNrNrUrGrGrArGrC	L5Cb	IDT custom synthesis
/5InvddT/ACACrGrArCrGrCrUrCrUrUrCrCrGr ArUrCrUrNrNrNrNrArCrUrCrArGrC	L5Cc	IDT custom synthesis
/5InvddT/ACACrGrArCrGrCrUrCrUrUrCrCrGr ArUrCrUrNrNrNrNrGrArCrUrUrArGrC	L5Cd	IDT custom synthesis
AATGATACGGCGACCACCGAGATCTACACT CTTTCCCTACACGACGCTCTTCCGATCT	P5	IDT custom synthesis
CAAGCAGAAGACGGCATACGAGATCGGTCT CGGCATTCCTGGCCTTGGCACCCGAGAATTCC	PE	IDT custom synthesis
Software and Algorithms		
STAR 2.5.0a	Dobin et al., 2013	N/A
Bedtools 2.26.0	Quinlan, 2014	N/A
Samtools 1.6	Li et al., 2009	N/A
R version 3.5.1 Patched (2018-11-02 r75543)	R Core Team, 2013	https://www.r-project.org/
ggplot2 3.1.0	Wickham, 2016	N/A
FASTX Toolkit 0.0.14		http://hannonlab.cshl.edu/fastx_toolkit/
pyCRAC	Webb et al., 2014	N/A
prinseq-lite-0.20.4	Schmieder and Edwards, 2011	N/A
bowtie2-2.3.4.1	Langmead and Salzberg, 2012	N/A

(*Continued on next page*)

Continued		
REAGENT or RESOURCE	SOURCE	IDENTIFIER
DESeq2	Love et al., 2014	N/A
RNAfold 2.1.5	Lorenz et al., 2011	N/A
cutadapt	Martin, 2011	N/A
StringTie 1.3.3b	Pertea et al., 2015	N/A
edgeR v3.16.5	Robinson et al., 2010	N/A

LEAD CONTACT AND MATERIALS AVAILABILITY

Further information and requests for resources and reagents should be directed to and will be fulfilled by the Lead Contact, Marc Bühler (marc.buehler@fmi.ch). All unique reagents generated in this study are available from the Lead Contact with a completed Materials Transfer Agreement.

EXPERIMENTAL MODEL AND SUBJECT DETAILS

Male 129 × C57BL/6 mouse embryonic stem cells (mESC) (Mohn et al., 2008) were grown in serum/LIF media (DMEM (GIBCO 21969-035) supplemented with 15% fetal bovine serum (GIBCO 10270106), 2 mM L-glutamine (GIBCO 25030024), 1x non-essential amino acids (GIBCO 11140035), 1 mM sodium pyruvate (GIBCO 11360070), 0.1 mM 2-mercaptoethanol (Sigma M-7522), 50 mg ml^{-1} penicillin, 80 mg ml^{-1} streptomycin and homemade LIF) at 37 °C in 5% CO2. Cells were cultured on dishes coated with 0.1% gelatin (Sigma G1890).

METHOD DETAILS

Generation of endogenously tagged cell lines

Endogenous gene tagging with a 3xFLAG-AviTag was performed in mES 129 × C57BL/6 cells expressing BirA ligase and CreERT2 from the *Rosa26* locus (cMB063) (Ostapcuk et al., 2018), using TALEN or CRISPR-Cas9 homology-directed repair with single-stranded oligodeoxynucleotide (ssODN) donor templates encoding the tag, flanked by 5′ and 3′ homology arms. The ssODNs donors were synthetized as ultramers by Integrated DNA Technologies. N-terminally tagged $Skiv2l^{3xFLAG\text{-}Avi/3xFLAG\text{-}Avi}$ clone 8F (cMB331) and $Aven^{3xFLAG\text{-}Avi/3xFLAG\text{-}Avi}$ clone 2B (cMB323) were generated using TALENs and Cas9/gRNA, respectively, cutting near the start codon. $Xrn1^{3xFLAG\text{-}Avi/3xFLAG\text{-}Avi}$ clone 4F (cMB315) was C-terminally tagged using Cas9/gRNA cutting near the stop codon. N-terminally tagged *Mtr4* cell lines (cMB376 and cMB503) were previously described (Tuck et al., 2018). C-terminally tagged $Rps10^{3xFLAG\text{-}Avi/3xFLAG\text{-}Avi}$ clone 4E (cMB395) was generated using Cas9/gRNA cutting near the stop codon. N-terminally tagged $Dis3l^{2xHA\text{-}FKBP12(F36V)/\ 2xHA\text{-}FKBP12(F36V)}$ (cMB510) was generated in the $Skiv2l^{3xFLAG\text{-}Avi/3xFLAG\text{-}Avi}$ (cMB331) background using Cas9/gRNA cutting near the start codon. For homology-directed repair, the donor sequence encoding the 2xHA-FKBP12(F36V) tag, flanked by ~550bp *Dis3l* 5′ and 3′ homology arms was cloned into a pBLU plasmid and transfected together with the Cas9/gRNA. All clones were screened for homozygous integration of the tag by PCR and Sanger sequencing and expression of the fusion proteins was confirmed by western blot with an anti-FLAG or anti-HA antibody. Biotinylation of the tag was verified by western blot using streptavidin-HRP. A full list of genome-edited cell lines together with TALENs, gRNAs and donor ssODN ultramer sequences can be found in Table S1.

Generation of straight KO cell lines

$Aven^{-/-}$ clones 4H (cMB399) and 6G (cMB400) were generated in a $Skiv2l^{3xFLAG\text{-}Avi/3xFLAG\text{-}Avi}$ background (cMB331) using Cas9/gRNAs targeting *Aven* exon 3 and exon 6 (last exon), resulting in a deletion of approximately 5.7 kb. $Focad^{-/-}$ clones 2F (cMB396) and 4B (cMB397) were generated in a $Skiv2l^{3xFLAG\text{-}Avi/3xFLAG\text{-}Avi}$ background (cMB331) with Cas9/gRNAs targeting intron 2 and intron 4. The resulting deletion of approximately 7.3 kb introduces a frameshift in exon 5. Homozygous knockout clones were screened by PCR and Sanger sequencing and deletion was confirmed by western blot or RT-qPCR. See also Table S1.

Generation of conditional KO cell lines

$Skiv2l^{fl/fl}$ cell lines were generated in a 129 × C57BL/6 WT background expressing a CreERT2 recombinase fusion from the *Rosa26* locus (cMB052) as well as in $Aven^{-/-}$ cells where *Skiv2l* is endogenously tagged (cMB399). A plasmid expressing Cas9 and gRNAs targeting *Skiv2l* intron 10 and intron 17 was co-transfected with ssODN containing homology arms and LoxP sites for integration. Recombination of the LoxP sites eliminates exons 11-17 containing the catalytic DExH box and results in out-of-frame translation of the last 18 exons. Clones with homozygous insertions of LoxP sites in both intron 10 and intron 17 were screened by PCR and Sanger sequencing. Proper recombination of the LoxP sites was tested by RT-qPCR, or western blot, following treatment with 0.1 μM 4-hydroxytamoxifen (4-OHT) (Sigma) for 2, 4 or 6 days. See also Table S1.

Transfections

For genome editing with CRISPR-Cas9, gRNAs were cloned into the SpCas9-2A-mCherry vector (Knuckles et al., 2017). To generate endogenously tagged *Xrn1* (cMB315), *Aven* (cMB323) and *Rps10* (cMB395), cells were transfected with 1000 ng SpCas9-2A-mCherry, 1400 ng ssODN donor and 100 ng pRRE GFP homologous recombination reporter (Flemr and Bühler, 2015). mCherry and GFP double-positive cells were FACS-sorted 24 hours after the transfection and seeded sparsely (10,000 cells) on 10 cm plates for clonal expansion. After 5-7 days, colonies were individually picked into 96-well plates, expanded and genotyped by PCR. Cells with proper in-frame homozygous insertions of the tag were further confirmed by Sanger sequencing and western blot.

For endogenous tagging of *Skiv2l* (cMB331) with TALENs, cells were transfected with 400 ng of each TALEN, 1000 ng ssODN donor and 100 ng of pRRP puromycin recombination reporter (Flemr and Bühler, 2015). 24 hours post-transfection, the cells were selected with 2 μg/ml puromycin for 28 hours and surviving cells were plated at clonal densities as described above. *Skiv2l*$^{fl/fl}$ cell lines were generated by transfecting 450 ng of each SpCas9-2A-mCherry gRNA plasmid, 500 ng of each LoxP ssODN donor and 50 ng of each pRRP puromycin reporter and selection with 2 μg/ml puromycin.

To create *Aven*$^{-/-}$ (cMB399 and cMB400) and *Focad*$^{-/-}$ (cMB396 and cMB397), cells were transfected with 500 ng of each SpCas9-2A-mCherry gRNA vector and 50 ng of each pRRP or pRRE-GFP reporter. *Aven*$^{-/-}$ cells were selected on 2 μg/ml puromycin and *Focad*$^{-/-}$ cells were selected by FACS-sorting mCherry-GFP double-positives.

To generate endogenously tagged *Dis3l*$^{2xHA\text{-}FKBP12(F36V)/\ 2xHA\text{-}FKBP12(F36V)}$ (cMB510), *Skiv2l*$^{3xFLAG\text{-}Avi/3xFLAG\text{-}Avi}$ (cMB331) cells were transfected with 500 ng SpCas9-2A-mCherry, 700 ng pBLU 2xHA-FKBP12(F36V) donor plasmid and 100 ng pRRP puromycin reporter. The cells were selected with 2 μg/ml puromycin and genotyped as described above. All transfections were carried out with Lipofectamine 3000 reagent at 3 μl per 1 μg of total DNA in OptiMem media. Approximately 500,000 cells were used for each transfection.

RNA sequencing

Total RNA was extracted from ~80% confluent 6 cm dishes using the Agilent Absolutely RNA Miniprep Kit with on-column DNase digestion. After ribosomal RNA depletion with the Illumina Ribozero kit, libraries were constructed using either ScriptSeq v2 or TruSeq v2 kits and sequenced on an Illumina HiSeq2500 platform (50 nt single-end reads). Total RNA from *Skiv2l*$^{fl/fl}$ conditional knockouts was extracted after culturing the cells in media supplemented with 0.1 μM 4OHT for 0, 2, 4 or 6 days to induce *Skiv2l* knockout.

To measure transcriptome-wide RNA half-lives, 300,000 mESCs were seeded per well of a six-well dish and grown for 48 h in serum + LIF medium. The medium was replaced by fresh medium with 5 μM actinomycin D (from a 5 mg/ml stock in DMSO) and the cells were incubated for 120, 240 or 360 min. A mock treatment (360 min) was included, using medium with the same amount of DMSO but no actinomycin D. After the indicated times, cells were washed twice with 37°C PBS and RNA extracted using the Agilent Absolutely RNA Miniprep kit. ERCC RNA spike-ins were added to the lysis buffer (1.7 μL of a 1:10 dilution per sample) before it was added to the cells. Three technical replicates were performed for each cell line, treatment and time point.

CRAC

CRAC was performed as described in (Tuck et al., 2018), with minor modifications, and is described in full here:

mESCs were grown in 2x 15-cm dishes to ~80% confluency, dishes washed 2x with PBS, the PBS removed, then cells crosslinked on ice (with dishes facing up) in a Stratagene Stratalinker 2400 (400 mJ cm^{-2}). Cells were lysed by incubating with 5 mL of either TN150+NP40 (50 mM Tris-HCl pH 7.8, 150 mM NaCl, 0.5% Nonidet P40 substitute and 1x cOmplete Protease Inhibitor Cocktail), or of RIPA (50 mM Tris-HCl pH 7.8, 300 mM NaCl, 1.0% Nonidet P40 substitute, 0.1% SDS, 10% (v/v) glycerol, 0.5% sodium deoxycholate, 1 mM beta-mercaptoethanol, 1x cOmplete Protease Inhibitor Cocktail), as indicated in Table S2. The harsher buffer (RIPA) was initially used to ensure complete extraction, but as this can reduce FLAG binding we later switched to a milder buffer (TN150), which did not affect library content. The cells were further disrupted using a cell scraper then lysates collected and centrifuged (6500 xg for 20 min at 4°C). Supernatants were frozen in liquid nitrogen and stored at −80°C.

Lysates were thawed on ice and incubated with 100 μL anti-FLAG M2 magnetic beads overnight. The supernatant was discarded and beads washed 3x with 1 mL TN150 (50 mM Tris-HCl pH 7.8, 150 mM NaCl, 0.1% Nonidet P40 substitute). Protein:RNA complexes were eluted by incubating beads in 1.5 mL TN150 supplemented with 5 mM beta-mercaptoethanol and 0.3 mg/ml 3xFLAG peptide, rotating at 4°C for 2 hr. The eluate was then incubated with 50 μL Dynabeads M-280 Streptavidin, rotating at 4°C overnight. Beads were washed 2x in TN600 (50 mM Tris-HCl pH 7.8, 600 mM NaCl, 0.1% Nonidet P40 substitute, 5 mM beta-mercaptoethanol) and 2x in TN150 supplemented with 5 mM beta-mercaptoethanol. RNA was fragmented by incubating beads in 500 μL TN150 supplemented with 5 mM beta-mercaptoethanol and 1 μL of 0.1 U diluted RNace-IT. After 4 min at 37°C, the RNases were denatured by replacing the solution with 400 μL WBI (50 mM Tris-HCl pH 7.8, 300 mM NaCl, 0.1% Nonidet P40 substitute, 5 mM beta-mercaptoethanol and 4.0 M guanidine hydrochloride). The beads were washed 2x in WBI, then 3x in 400 μL 1xPNK (50 mM Tris-HCl pH 7.8, 10 mM $MgCl_2$, 0.5% Nonidet P40 substitute, 5 mM beta-mercaptoethanol).

The following four enzymatic reactions were then performed in 80 μL 1xPNK buffer (omitting the Nonidet P40 substitute), to ligate 3′ and 5′ adapters onto RNA fragments. After each reaction, beads were washed 1x in WBI and 3x in 1xPNK:

(i) Alkaline phosphatase treatment (30 min, 37°C): 8 U TSAP, 80 U RNasIN.

(ii) 3′ linker ligation (overnight, 16°C): 0.1 nmol miRCat-33 DNA linker, 40 U T4 RNA Ligase 1, 80 U RNasIN, 12.5% (v/v) PEG8000.
(iii) 5′ phosphorylation (1 hr, 37°C): 40 U T4 PNK, 2 μL γ32P-ATP (after 30 min, add 1 μL 100 mM rATP and an additional 20 U T4 PNK).
(iv) 5′ linker ligation (overnight, 16°C): 0.2 nmol 5′ linker, 40 U T4 RNA Ligase 1, 1.25 mM rATP, 80 U RNasIN, 12.5% (v/v) PEG8000.

After the final reaction, beads were washed 3x in WBII (50 mM Tris-HCl pH 7.8, 50 mM NaCl, 0.1% Nonidet P40 substitute, 5 mM beta-mercaptoethanol), resuspended in 30 μL 1x NuPAGE LDS sample buffer, heated at 95°C for 2 min, and the eluate quickly removed and loaded onto a NuPAGE 4%–12% polyacrylamide gel. The gel was run at 100 V for ~1 hr, then protein:RNA complexes transferred to Hybond-C extra nitrocellulose membrane (Amersham) at 150 V for 1.5 hr using a wet transfer system and NuPAGE transfer buffer with 15% methanol. The membrane was then briefly dried, exposed to BioMax MS film (4 hr to overnight) and the region corresponding to the protein:RNA complex cut out.

The membrane slice was then incubated in 400 μL WBII with 1% (w/v) SDS, 5 mM EDTA and 100 μg Proteinase K at 55°C for 2 hr. The solution was then removed to another tube, 50 μl 3M NaAc pH 5.2 and 500 μl of 1:1 phenol:chloroform mix added, and the mixture vortexed then centrifuged at 14,000 xg for 20 min. The top phase was transferred into a new tube and 1 mL ethanol and 20 μg glycogen added. The solution was stored at −20°C overnight to precipitate RNA, then centrifuged at 14,000 xg for 1 hr. The pellet was washed once with 70% ethanol and allowed to briefly air dry, before resuspending in 11 μL water + 1 μL 10 μM miRCat-33 RT oligo + 1 μL 10 mM dNTP mix. The solution was heated to 80°C for 3 min, snap cooled on ice for 5 min, then the following mix added: 4 μL 5x first strand buffer (SSIII kit) + 1 μL 100 mM DTT (SSIII kit) + 1 μL recombinant RNasIN. After incubating for 3 min at 50°C, 200 U of SuperScript III was added and the reverse transcription allowed to proceed for 1 hr at 50°C. The reaction was stopped by heating to 65°C for 15 min, then RNA digested with 10 U RNase H at 37°C for 30 min. PCR reactions (80 μL) were then prepared, each with 2 μL cDNA, 10 pmol P5, 10 pmol PE, 12.5 nmol each dNTP and 2.5 U LA Taq. Typically, we ran five PCR reactions and then concentrated the products by ethanol precipitation before resolving on a 3% metaphor agarose gel in 0.5x TBE. A smear corresponding to the size of the two adapters plus inserts (total size ~120-300 bp) was then excised, and DNA extracted using the MinElute gel extraction kit, eluting in 20 μL water. If the experiment was successful, we repeated the PCRs with the remaining half of the cDNA.

The above CRAC protocol is referred to as the "long" protocol. For some samples (indicated in Table S2), a shorter version of the protocol was used, which did not affect library content. The shorter version omits radiolabelling (using cold rATP instead), SDS-PAGE and transfer to nitrocellulose. Instead, after 3′ linker ligation, beads were washed and added directly to 400 μL WBII with 1% (w/v) SDS, 5 mM EDTA and 100 μg Proteinase K. This version of the CRAC protocol is referred to as the "short" protocol (indicated in Table S2).

Translation inhibition experiments for CRAC

Cells grown to ~80% confluency on 15cm dishes were incubated with media supplemented with either 100 μg/mL cycloheximide or 5 μM harringtonine for 30 min at 37°C. Cells were then washed twice with PBS containing the same concentration of the corresponding inhibitors. PBS was removed after the last wash and the cells were cross-linked on ice, with the dishes facing up, in a Stratagene Stratalinker 2400 (400 mJ·cm−2) and processed for CRAC as described above.

Ribosome profiling

Cells were harvested (without cycloheximide pretreatment) and flash-frozen in liquid nitrogen. From the cell pellets, lysates were prepared and ribosome-protected mRNA fragments were generated by RNase I digestion as previously described (Janich et al., 2015). For the excision of footprints from 15% urea-polyacrylamide gels, single strand RNA oligonucleotides of 26 nt and 34 nt (for monosome footprints) and of 52 nt and 69 nt (for disome footprints) served as size markers for excision of footprints. After fragment purification with miRNeasy RNA Extraction kit, 5μg fragmented RNA was used for ribosomal RNA removal using Ribo-Zero Gold rRNA Removal Kit according to Illumina's protocol for TruSeq Ribo Profile (RPHMR12126 Illumina).

Sequencing libraries were generated according to Illumina's TruSeq Ribo-Profile protocol with minor modifications. Monosomes and disomes were treated as independent libraries. cDNA fragments were separated on a 10% urea-polyacrylamide gel and gel slices between 70-80 nt for monosomes and 97-114 nt for disomes were excised. The PCR-amplified libraries were size selected on an 8% native polyacrylamide gel. Monosome libraries were at ~150 bp and disome libraries at ~180 bp.

Parallel RNA-seq libraries were prepared essentially following the Illumina protocol (Janich et al., 2015); briefly, after total RNA extraction using miRNeasy RNA Extraction kit, ribosomal RNA was depleted using Ribo-Zero Gold rRNA Kit, and sequencing libraries generated from the heat-fragmented RNA as described (Janich et al., 2015). All libraries were sequenced on Illumina HiSeq 2500.

Western Blotting

Cells were lysed for 30 min on ice in 50 mM Tris-HCl, pH 7.5, 150 mM NaCl, 1% Triton-X, 0.5 mM EDTA, 5% glycerol, 1x protease inhibitor cocktail (Roche) and 1 mM DTT. Lysates were clarified by centrifugation at 16,000 xg for 10 min at 4°C and protein concentration was measured using the BioRad protein assay. Approximately 20 μg of total protein extract was resolved on

NuPAGE-Novex Bis-Tris 4%–12% gradient gels (Thermo Fisher NP0322BOX), transferred semi-dry to a polyvinylidene fluoride (PVDF) membrane, blocked in 5% non-fat milk in TBS+0.05% Tween (TBST) for 30 min at room temperature and incubated with primary antibodies at 4 °C overnight. The following primary antibodies were used for western blotting: mouse anti-Flag (1:1,000, Sigma clone M2), rabbit anti-AVEN (1:2,000, ProScience 2417), rabbit anti-ATF4 (1:1,000, Cell Signaling D4B8 mAb11815), rabbit anti- Phospho-eIF2α Ser51 (1:1,000, Cell Signaling D9G8 mAb3398), rabbit anti-eIF4E (1:1,000, Bethyl A301-154A) and rat anti-tubulin (1:5,000, Abcam clone YL1/2). Following incubation with corresponding HRP-conjugated secondary antibodies, signal was visualized using Immobilon Western Chemiluminiscent HRP Substrate. To detect biotinylated proteins, after transfer, membranes were blocked in 2% bovine serum albumin (BSA) in TBST for 30 min and incubated with HRP-conjugated streptavidin (Strep-HRP) diluted 1:10,000 in 2% BSA-TBST for 30 min at room temperature. For detection of ATF4 and Phospho-eIF2α Ser51, membranes were first probed for ATF4, stripped in 25 mM Glycine, pH 2 and 1% SDS for 5 min at room temperature, rinsed with TBST, blocked in 5% non-fat milk TBST for 30 min and re-probed for Phospho-eIF2α Ser51.

Co-immunoprecipitations

Dis3l$^{2xHA\text{-}FKBP12(F36V)/2xHA\text{-}FKBP12(F36V)}$ (cMB510) cells grown to ~80% confluency in a 10 cm dish were trypsinized, collected in media and washed twice with PBS. The cells were lysed for 40 min at 4°C in 500 μl lysis buffer (10 mM Tris-HCl, pH 7.4, 150 mM NaCl, 2.5 mM $MgCl_2$, 0.5% NP-40), supplemented with 1X protease inhibitor cocktail (Roche). Lysates were clarified by centrifugation at 16,000 g for 5 min and mixed with 30 μl Protein-G Dynabeads (Thermo Fisher 10004D) coupled to 2 μg anti-HA antibody (Roche 11867423001). The sample was incubated for 1 hour at 4°C on a rotating wheel. The beads were then washed four times in wash buffer (10mM Tris-HCl, pH 7.4, 150 mM NaCl, 2.5 mM $MgCl_2$, 0.1% NP-40), resuspended in 60 μl 1X Bolt LDS Sample Buffer (Thermo Fisher B0007) and incubated at 85°C for 5 min to elute captured proteins from the beads. Following this, 2% of the input and 30% of the IP material were resolved on a NuPAGE-Novex Bis-Tris 4%–12% gradient gel (Thermo Fisher NP0322BOX), transferred semi-dry to a polyvinylidene fluoride (PVDF) membrane, blocked in 5% non-fat milk in TBS+0.05% Tween (TBST) for 30 min at room temperature and incubated with primary antibodies at 4 °C overnight. The following primary antibodies were used for western blotting: rat anti-HA (1:1,000, Roche 11867423001), mouse anti-Flag (1:1,000, Sigma clone M2), rabbit anti-Mtr4 (1:1,000, Thermo Fisher PA5-57927).

Affinity purification for LC–MS/MS

For tandem FLAG-streptavidin affinity purification, two confluent 15 cm dishes seeded with equal number *Skiv2l*$^{3xFLAG\text{-}Avi/3xFLAG\text{-}Avi}$ cells or the corresponding untagged parental line were harvested by trypsinization, washed twice in PBS and lysed 2 hours to overnight at 4°C in whole cell lysis buffer (10 mM Tris-HCl pH 7.4, 150 mM KCl, 2.5 mM $MgCl_2$, 0.5% NP-40), supplemented with 1X protease inhibitor cocktail, 50 units benzonase and 10 μg RNase A. Lysates were clarified by centrifugation at 16,000 g for 15 min and incubated with 20 μl anti-FLAG M2 Dynabeads for 4 hours at 4°C. After washing the FLAG beads three times with wash buffer (10mM Tris-HCl (pH 7.4), 150 mM KCl. 2.5 mM $MgCl_2$, 0.1% NP-40), proteins were eluted from the beads three times for 15 min at 4°C with 100 μg/ml 3xFLAG peptide diluted in 150 μl wash buffer. The eluates were combined and incubated with 20 μl M-280 Streptavidin Dynabeads for 2 hours at 4°C, washed four times in wash buffer and two times in wash buffer without NP-40. For mass spectrometry analysis, captured proteins were digested with trypsin directly on the streptavidin beads. High-salt tandem FLAG-strep affinity purifications were essentially carried out as described above with the following modifications: cells from two confluent 10 cm dishes were lysed in buffer containing 350 mM KCl. For single-step streptavidin pull-downs, the FLAG purification step was omitted and total cell lysates from two confluent 10 cm dishes were applied directly to streptavidin beads. Every affinity purification experiment contained three separate technical replicates for each cell line.

Mass spectrometry analysis

Peptides generated by trypsin digestion (see 'Affinity purification for LC–MS/MS') were acidified with 0.8% TFA (final) and analyzed by LC–MS/MS on an EASY-nLC 1000 with a two column set-up (Thermo Scientific). The peptides were applied onto a peptide trap (Acclaim PepMap 100, 75 μm × 2 cm, C18, 3 μm, 100 Å) in 0.1% formic acid, 2% acetonitrile in H_2O at a constant pressure of 80 MPa. Using a flow rate of 150 nl min^{-1}, peptides were separated with a linear gradient of 2%–6% buffer B in buffer A in 3 min followed by an linear increase from 6 to 22% in 40 min, 22%–28% in 9 min, 28%–36% in 8 min, 36%–80% in 1 min and the column was finally washed for 14 min at 80% buffer B in buffer A (buffer A: 0.1% formic acid; buffer B: 0.1% formic acid in acetonitrile) on a 50 μm × 15 cm ES801 C18, 2 μm, 100 Å column (Thermo Scientific) mounted on a DPV ion source (New Objective) connected to a Orbitrap Fusion (Thermo Scientific). Data acquisition was performed using 120,000 resolution for the peptide measurements in the Orbitrap and a top T (3 s) method with HCD fragmentation for each precursor and fragment measurement in the ion trap following the manufacturer guidelines (Thermo Scientific).

Peptide identification was performed with MaxQuant version 1.5.3.8 using Andromeda as search engine (Cox et al., 2011). The mouse subset of the UniProt version 2015_01 combined with the contaminant DB from MaxQuant was searched and the protein and peptide FDR values were set to 0.05. All MaxQuant parameters can be found in Table S5.

Statistical analysis was done in Perseus (version 1.5.2.6) (Tyanova et al., 2016). Results were filtered to remove reverse hits, contaminants and peptides found in only one sample. Missing values were imputed and potential interactors were determined using t test and visualized by a volcano plot. Significance lines corresponding to an FDR of 0.05 and S0 (curve bend) between 0.2 and

2.0 are shown in the corresponding Figures. Results were exported from Perseus and visualized using statistical computing language R.

Cell cycle analysis

Cells were synchronized at the G1/S boundary using a double-thymidine block. Briefly, 300,000 cells of each indicated cell line were seeded on 6-well plates and grown overnight in normal serum/LIF media, or media containing 0.1 μM 4-OHT to induce *Skiv2l* knockout where necessary. On the following day, the cells were switched to media supplemented with 2 mM Thymidine and cultured for 18 hours, released into the cell cycle for 9 hours by removal of the drug with three PBS washes and cultured in 2 mM thymidine media for another 18 hours. The cells were then released from the second block by three PBS washes and harvested at 0, 4 and 8 hours after thymidine withdrawal. For each sample, equal number of cells were fixed in ice-cold 70% ethanol and incubated overnight at 4°C. The cells were then permeabilized with PBS + 0.1% triton X200 for 2 min, stained with 1 μg/mL DAPI in PBS+0.1% triton X200 and DNA content was analyzed by flow cytometry. Histogram plots were generated using the FlowJo software.

ATF4 induction with Thapsigargin

Approximately 120,000 cells per sample were seeded in 24-well plates and cultured in normal serum/LIF media overnight. On the following day the cells were switched to media supplemented with 200 mM Thapsigargin to induce the integrated stress response and upregulation of ATF4, and harvested for western blot analysis at 0, 0.5, 2 or 4 hours of incubation with the drug.

RT-qPCR

Total RNA was extracted from mES cells with the Agilent Absolutely RNA Miniprep Kit and 500ng of RNA was reverse-transcribed using the PrimeScript RT-PCR Kit. qPCR was performed with SsoAdvanced SYBR Green Supermix on a CFX96 Real-Time PCR System (Bio-Rad) and relative RNA levels were calculated using the ΔC_t method and normalization to TBP mRNA abundance. A list of qPCR primers is provided in Table S1.

QUANTIFICATION AND STATISTICAL ANALYSIS

CRAC data preprocessing and alignment

CRAC reads were preprocessed with the FASTX Toolkit 0.0.14. Adapters were removed with fastx_clipper, low quality bases trimmed/reads removed using fastq_quality_trimmer -t 25 and fastq_quality_filter -q 20 -p 90, and sequencing artifacts removed using fastx_artifacts_filter. Duplicate reads (including UMI) were collapsed, then pyCRAC (Webb et al., 2014) used to split samples by their inline barcodes and extract the UMIs . Low complexity regions were removed (“low complexity stripping”) from the 3′ end of sequences (defined as stretches of 2 nt more where 80% of positions are the same nucleotide, e.g., AAAAGAA), and prinseq-lite-0.20.4 (Schmieder and Edwards, 2011) used as an additional filter to remove low complexity reads (settings -lc_threshold 20 -lc_method dust). We then applied a set of filters to obtain uniquely mapping reads, and remove reads mapping to repeats or abundant non-coding RNAs (e.g., tRNA, snoRNA or rRNA). For this, reads were separately mapped with bowtie2-2.3.4.1 (Langmead and Salzberg, 2012) (settings–local -p 10 -a–very-sensitive) to three indexes:

Genome: mm10 genomic sequence.

Gencode non-coding RNAs: Gencode release M16 Mt_rRNA, Mt_tRNA, miRNA, rRNA, scRNA, snoRNA, sRNA, scaRNA and snRNA features from the file gencode.vM16.annotation.gtf, and predicted tRNAs from the file gencode.vM16.tRNAs.gtf, and a repeat-masked version of mouse pre-rRNA (Grozdanov et al., 2003).

Gencode mRNAs/lincRNAs: Gencode release M16 protein_coding and lincRNA features from the file gencode.vM16.annotation.gtf.

Reads were assigned as “repeats/ncRNAs” and excluded if they mapped best or equally well either to regions of the genome overlapping RepeatMasker repeats (downloaded from UCSC table browser, version 2012-02-07) or to Gencode non-coding RNAs. From the remaining reads, those mapping better or equally well to Gencode mRNA/lincRNAs as to the genome were extracted. A filter was then applied, by mapping these reads (bowtie2-2.3.4.1–local -p 10 -a–very-sensitive) to an index with one transcript isoform per protein-coding gene (the APPRIS principal isoform was selected, taking one at random if a gene possessed multiple; were refer to these as “Appris transcripts”), and selecting reads with (i) a second best match score (if detected) < 0.8 times the best match score and (ii) a MAPQ $\geq$ 8. Reads were removed if the 5′ end was soft-clipped, and duplicate reads collapsed based on their UMIs and 5′ end mapping positions (retaining one read at random).

For comparing CRAC data with ribosome profiling, an identical procedure was used, except using a list of transcripts robustly detected in the ribosome profiling experiments instead of Appris transcripts. Where multiple transcripts were detected from the same gene, alignments were prioritized to the primary isoform if it could be determined (see ribosome profiling methods).

CRAC quantification of non-templated 3′ tails

To identify reads containing non-templated 3′ tails, preprocessed reads identified as uniquely mapping to Appris transcripts were extracted, then filtered to retain those for which the 3′ adaptor could be identified and stripped. Bowtie2-2.3.4.1 was then used to align these reads (which were not subject to low complexity stripping from the 3′ end) to the genome, transcriptome and Appris transcripts (defined above), with the following settings, as described in (Travis et al., 2014): -D 20 -R 3 -N 0 -L 16–local -i S,1,0.50–score-min L,18,0–ma 1–np 0–mp 2,2–rdg 5,1–rfg 5,1. Tails were extracted by identifying examining alignments for 3′ soft clipping. Reads were required to align better to the transcriptome than to the genome (to prevent "tails" being identified that in fact correspond to exon-exon junctions). Only homopolymeric tails were analyzed, with no minimum length requirement.

CRAC PCA, correlation matrix and tSNE

Filtered, uniquely mapping CRAC reads were counted for all mRNAs (using alignments to Appris transcripts, as defined above, and thus excluding reads mapping to introns). Each CRAC replicate was processed separately. For tSNE analysis, CRAC replicates were pooled, to give one dataset each for MTR4, SKIV2L and XRN1. These three count datasets were then normalized to the sum of the smallest dataset, and mRNAs retained with > 1 normalized count in all datasets, and > 10 normalized counts in at least one dataset. To obtain "relative binding" to MTR4, SKIV2L and XRN1, for each transcript the normalized counts were divided by its total normalized counts. Therefore, for each transcript, the "relative binding" of MTR4 + SKIV2L + XRN1 sums to 1. In parallel, to check that small differences in transcript levels between cell lines do not distort the analysis, relative binding values were normalized to rpkm values taken from RNA-seq analysis of the three cell lines.

Differential SKIV2L binding analysis

As genes were differentially expressed in *Aven*$^{-/-}$ cells versus WT, an interaction model accounting for differences in transcript levels, as described by (Chothani et al., 2017), was used to compare SKIV2L binding for these two cell lines.

Identification of rRNA binding sites by CRAC

The human ribosome structure was downloaded from PDB (4UG0) and its 18S rRNA sequence extracted. This was then substituted to match the mouse 18S rRNA sequence where possible, to facilitate alignment. CRAC reads with adapters and barcodes removed were aligned to this modified 18S rRNA sequence using bowtie2-2.2.3 (–sensitive mode). Alignments were filtered to remove those less than 20 bp long, or with an edit distance > 1. Reads were then piled up across the modified 18S rRNA sequence using samtools-1.3 (depth command) (Li et al., 2009). For each CRAC sample, these values were converted to single position counts per million, then mean normalized counts calculated for 20 nt bins across the entire 18S rRNA. This was repeated for 99 CRAC datasets, including several replicates each for SKIV2L, AVEN and XRN1, and an in-house collection of datasets from many unrelated proteins or untagged cell lines which served as controls. This extensive control dataset enabled specific signal to be robustly distinguished from background or technical artifacts. We also included additional SKIV2L, AVEN and XRN1 datasets for which global coverage was too low for mRNA analysis, but rRNA coverage sufficiently high for rRNA analysis. All raw data are deposited in GEO under accession GSE134020 and the control datasets indicated.

To quantify specific binding of AVEN, a negative binomial model was then used to fit the AVEN and control values for each 20 nt 18S rRNA bin. This model contained AVEN versus control as a factor, and was compared to a null (intercept only) model using a $\chi 2$ test (accounting for multiple hypothesis testing using the Benjamini Hochberg method with an FDR of 0.05). The $\chi 2$ p values were then used to color significantly bound regions of the 18S rRNA, in the context of the 40S ribosome structure. This procedure was repeated for SKIV2L and XRN1. Note that SKIV2L and XRN1 were included as controls for each other, and XRN1 (but not SKIV2L) was included as a control for AVEN.

CRAC plots around start and stop codons

Uniquely mapping CRAC reads were piled up across each Appris transcript, and each transcript normalized by dividing by its the maximum read depth, and transcripts with fewer than five reads excluded. These normalized values were then either plotted around the start or stop codon of each individual transcript (arranging transcripts by 5′ UTR or 3′ UTR length), or values were summed to produce a metaplot.

CRAC enrichment at amino acid combinations

The 3′ end positions of filtered uniquely mapping (but not low complexity stripped) SKIV2L CRAC reads were extracted for all mRNAs. These reads were further filtered to retain only those for which the 3′ adaptor had been identified and removed (so the 3′ end of the remaining read corresponds to the true 3′ end of a captured RNA fragment). Taking mRNAs with at least five CRAC reads passing these filters, 204 nt sliding windows were generated across the CDS, with an offset of 1 between each window. For each window, the hexamer (6 nt sequence) at its center was recorded, together with its frame (0, 1 or 2) relative to the start codon. SKIV2L CRAC 3′ ends were then piled up across every window for each transcript, and windows with at least five counts retained. The values for each position of a given window were then divided by the sum for that window. Windows were then grouped by their central hexamer (e.g., AAAAAG) and its frame (e.g., 0), and pileups summed for each group, dividing by the number windows within each

group. This provides an average distribution of SKIV2L-bound RNA fragment 3′ ends around every 6 nt motif, for frames 0, 1 and 2. This process was repeated for XRN1, RPS10 (a ribosomal protein) and TRIM71 CRAC datasets, which were used as controls.

For each dataset, hexamer and frame, the values for the central 6 nt were summed (these correspond precisely to the hexamer). "SKIV2L-bound" hexamer/frame combinations were then defined as those for which the SKIV2L value was higher than any of the other (control) datasets, and higher than that expected if SKIV2L reads had been distributed evenly across the 204 nt window. Hexamers were then translated into amino acid pairs (e.g., AAAAAG = > KK), then for each amino acid pair, the proportion of SKIV2L-bound hexamers calculated. This was repeated for in-frame hexamers (frame 0) and out of frame hexamers (frames 1 and 2). Only amino acid pairs with at least four contributing hexamers were evaluated. These final values (ranging from 0 to 1) give an indication of whether SKIV2L binds preferentially to particularly amino acid pairs, and whether this is frame specific (i.e., likely to reflect the encoded amino acids) or not (i.e., likely to reflect the underlying sequence).

CRAC and disome profiling repeat analyis

For plots around amino acids repeats, the 3′ ends of uniquely mapping CRAC reads (not low complexity stripped) for which adapters were detected and removed, and uniquely mapping ribosome profiling reads, were used. These were piled up across windows centered on all 3-4 amino acid repeat tracts in Appris mRNAs (e.g., KKKK, AAA, etc), including 96 nt either side. Data were binned into 6 nt bins and normalized to the maximum count for each window. Values were then summed for each repeat type (e.g., K, A, etc) at each position.

For plots around polypurine ($[G/A]_{12+}$) tracts, a similar approach was used, piling up CRAC 3′ read positions or ribosome profiling reads across windows centered on the GA tract and including 96 nt flanks. For each window, pileups were normalized to the total counts, then pileups summed for windows grouped by G or A content, or grouped by the encoded amino acids (e.g., those with > 30% lysine). Note that repeat tracts were only identified in-frame, and were required to be a multiple of three nt long (to enable them to be translated into an amino acid sequence).

RNA-seq analysis

RNA-seq reads were aligned to mm10 and counted using STAR_2.5.0a (–runMode alignReads –outSAMtype BAM SortedByCoordinate–outFilterType BySJout–outFilterMultimapNmax 1–outFilterMismatchNmax 3–outSAMmultNmax 1–out SAMattributes NH HI NM MD AS nM–outMultimapperOrder Random–outSAMunmapped None–quantMode GeneCounts). The STAR index was made using the Mus_musculus.GRCm38.75.dna.primary_assembly.fa file, providing gencode.vM16.annotation.gtf as the sjdbGTF file. DESeq2 (Love et al., 2014) was used to test for differential gene expression, with the model formula including biological clone (where at least two were available), sequencing batch (where more than one was performed) and genotype/treatment (WT versus knockout, or time of 4OHT treatment).

RNA half-life analysis

Mapped RNA-seq reads (ERCC sequences were included as extra chromosomes for the mapping) were counted for each gene and ERCC using the–quantMode GeneCounts mode in STAR. Counts were normalized separately for each time point using the estimateSizeFactorsForMatrix function from DESeq2, then recombined. A single size factor was calculated for each time point to account for the overall decay of mRNAs. For this, the ratio of total mRNA counts to total ERCC counts (using the set of ERCCs with a mean of > 50 counts across all samples) was calculated for each sample. A median value (size factor) was then calculated for each time point, size factors scaled so that the size factor for time = 0 was 1, then all mRNA count tables for a given time point divided by the corresponding size factor.

Half-lives were then calculated by fitting a linear model for ln(normalized counts + 1) versus time, and using the formula $t_{1/2} = -\ln(2)/k$, where k is the coefficient for time (i.e., the gradient of the linear fit in semi-log space). The residual standard error was also calculated, as a measure of fit.

CRAC sequence and structure motif analysis

Our approach was based on that described in (Welte et al., 2019). Filtered CRAC reads aligned to mRNAs robustly detected in ribosome profiling experiments were counted for 50 nt sliding windows (offset 10 nt) across the 5′ UTRs and CDSes of these transcripts. These windows were also folded *in silico* using RNAfold 2.1.5 (Lorenz et al., 2011) (including the option -g, to incorporate G-quadruplex formation into the prediction). A given "foreground" sample (e.g., AVEN) together with several control datasets ("background" samples, comprising MTR4, XRN1, RPS10 (a ribosomal protein) and TRIM71 (Welte et al., 2019) were analyzed, and for each dataset, counts converted to RPKM. Median RNA-seq RPKM values for our SKIV2L, XRN1 and MTR4 tagged mESCs were also extracted for each transcript, removing transcripts with < 10 RPKM. CRAC values were then divided by RNA-seq values to obtain enrichments for each window and foreground or background dataset. "Bound" windows were defined as those where the foreground enrichment was higher than any of the background enrichments, and at least 10 (i.e., 10x CRAC coverage versus RNA-seq coverage). The highest bound 5′ UTR and CDS window was then selected for each transcript (final foreground window set). Transcripts with fewer than six analyzed windows were excluded. As a control, windows were randomly selected from the same transcripts, requiring them to be within 400 nt of the foreground window set, and excluding the foreground window set. This process was repeated 100 times, to generate a 100 final background window sets.

For the final foreground and each final background window set, the total number of windows containing each possible 10-mer structural motif (based on the RNAfold output) or 6-mer sequence motif were counted. For each motif, the mean background total and its standard deviation were calculated, and used to calculate a z-score (foreground minus mean background occurrence, divided by background standard deviation). The z-score was plotted for each motif, versus its log_2 total occurrence (foreground plus mean background). Motifs with a z-score magnitude > 2.5, and sufficiently high log_2 total occurrence, were highlighted.

To compare SKIV2L CRAC data from WT and *Aven*$^{-/-}$ cells, the same approach was used, except both rather than comparing the number of bound windows containing each structure/sequence motif to a randomly sampled set of windows, SKIV2L-bound windows for the two cell lines were compared directly.

CRAC/ribosome profiling at structured regions

For mRNAs robustly detected in ribosome profiling experiments, 50 nt non-overlapping windows were defined across the 5′ UTR and CDS, and folded *in silico* using RNAfold 2.1.5. Windows with a minimum free energy < -12 kcal/mol and continuous stretch of ≥ 10 paired nucleotides were selected, and extended by 96 nt either side. CRAC and ribosome profiling reads were piled up across these 242 nt windows, and these values normalized for each dataset and window to the window sum. Windows were then divided into 6 nt bins, and normalized counts summed for each bin and plotted.

CRAC/ribosome profiling for genomic windows

For this analysis, CRAC reads uniquely mapping to the genome were used. RNA-seq reads, and ribosome profiling reads that had been trimmed, quality filtered, size selected ([26,35] for monosome footprints, [45,70] for disome footprints and [21,70] for total input RNA), and filtered against rRNA and tRNA libraries, were mapped to the genome using STAR_2.5.0a (settings–runMode alignReads–outSAMtype BAM SortedByCoordinate–outFilterType BySJout–outFilterMultimapNmax 1–outFilterMismatchNmax 3–outSAMmultNmax 1–outSAMattributes NH HI NM MD AS nM–outMultimapperOrder Random–outSAMunmapped None–quantMode GeneCounts. CRAC, RNA-seq and ribosome profiling reads were then counted (in a strand specific manner) for 1 kb windows tiling the genome in both orientations, generated using bedtools (Quinlan, 2014).

Windows were also overlapped with protein-coding genes, or genes encoding abundant ncRNAs (e.g., rRNA, snRNA and snoRNA genes) but not lincRNAs. This enabled windows to be classified based upon whether they overlapped abundant ncRNAs, mRNA exons (sense orientation), mRNA introns (sense orientation), 1 kb regions upstream of mRNAs (sense orientation), mRNA exons (antisense orientation), mRNA introns (antisense orientation) or 1 kb regions upstream of mRNAs (antisense orientation), with priority given in that order (i.e., if a window overlapped an mRNA exon and a ncRNA, it would be classified as a ncRNA window). All other windows were classified as intergenic. GC contents, predicted minimum free energy (using RNAfold), potential small ORFs (sequences starting with ATG and ending with an in-frame stop codon), and average phyloCSF scores for these small ORFs, were also calculated for each window.

Window counts were then normalized using the DESeq2 function “estimateSizeFactorsForMatrix” (for RNA-seq and ribosome profiling) or to the minimum library size (for CRAC). Normalized counts were then log_2 transformed, including a pseudocount of 4. Log_2 fold changes were then calculated for *Aven*$^{-/-}$ versus WT datasets, or comparing CRAC datasets (e.g., AVEN versus SKIV2L), as indicated. Genomic windows were also classified based on their SKIV2L CRAC counts in *Aven*$^{-/-}$ versus WT, into the categories “down,” “slight change,” “up” and “strongly up.” This enabled log_2 fold changes (e.g., comparing RNA-seq for *Aven*$^{-/-}$ versus WT) to be median centered on the “down” category of windows, facilitating comparison of different data types. Where more than one batch was available for RNA-seq datasets, batches were merged by calculating mean values for each window at the end of the above procedure.

Ribosome profiling analysis

Preprocessing of Ribosome Footprints

Initial quality assessment of the sequencing reads was conducted based on the preliminary quality values produced by the Illumina pipeline 2.19.1 such as the percentage of clusters passed filtering (%PF clusters) and the mean quality score (PF clusters). Adaptor sequences were removed using cutadapt utility (Martin, 2011) with following options: -a AGATCGGAAGAGCACACGTCT GAACTCCAGTCAC–match-read-wildcards. Next, trimmed read sequences were filtered by their size using an in-house Python script with following inclusive ranges: [26,35] for monosome footprints, [45,70] for disome footprints and [21,70] for total RNA. Smaller or larger fragments were kept separately and not used in further analysis. Finally, the reads were filtered for quality using fastq_quality_filter tool from the FASTX-toolkit with the following arguments: -Q33 -q 30 -p 80.

Mapping of Footprints to Mouse Genome

The preprocessed insert sequences were mapped sequentially to following databases: mouse rRNA, human rRNA, mt-tRNA, mouse tRNA, mouse cDNA from Ensembl mouse database release 91 (Flicek et al., 2013) and, finally, mouse genomic sequences (Genome Reference Consortium GRCm38.p2). In all but the final mapping against genomic sequences, bowtie version 2.3.0 (Langmead and Salzberg, 2012) was used with the following parameters: -p 2 -L 15 -k 20–no-unal,

After each alignment, only reads that were not aligned were used in the following mapping. For further analysis, only alignments against mouse cDNA were used, unless specifically stated otherwise. For each query sequence, only alignments with maximum alignment scores were kept.

Separately from this sequential alignment strategy, trimmed and filtered total RNA sequences from each sample were also directly aligned against the mouse genome. This mapping and the final mapping of the sequential alignment strategy were performed using STAR version 2.5.3a (Dobin et al., 2013) with the following parameters:

```
--runThreadN 6 –genomeDir=mouse/star/Mmusculus.GRCm38.91
--readFilesCommand zcat –genomeLoad LoadAndKeep
--outSAMtype BAM SortedByCoordinate Unsorted
--alignSJDBoverhangMin 1 –alignIntronMax 1000000
--outFilterType BySJout –alignSJoverhangMin 8
--limitBAMsortRAM 15000000000
```

The output of this alignment was used to estimate expressed transcript models out of all models contained in Ensembl mouse database release 91. To this end, we used StringTie version 1.3.3b (Pertea et al., 2015) to estimate the number of fragments per kilo base of exon per million fragments mapped (FPKM) for each transcript, with the following parameters:

```
-p 8 -G Mmusculus.GRCm38.91.gtf -A gene_abund.tab
-C cov_refs.gtf -B -e
```

The resulting FPKM estimate information was parsed with an in-house Python script to identify transcripts which had an FPKM > 0.2 and an isoform abundance fraction > 0.05 in at least 2 samples. A database of expressed transcripts based on this filtering was used in further analysis. Among those, genes that were estimated to have a single expressed isoform were annotated as single transcript genes.

Quantification of Footprint Abundance/Density

Abundance of mRNA and monosome or disome protected fragments was estimated per gene as described in (Janich et al., 2015). For this quantification, only reads that were mapped uniquely to a single gene and only to transcripts that were identified to be expressed (see Mapping of Footprints to Mouse Genome) were used. We used a limited size range of disome fragments (56-64 nt), as this facilitated subsequent A-site assignment and high-resolution analysis of stall sites.

Read counts of total RNA and RPF were normalized with upper quantile method of R package edgeR v3.16.5 (Robinson et al., 2010). Prior to normalization, transcripts which did not have at least 10 counts in at least one third of the samples were removed from the datasets. For better comparability between datasets, RPKM values were calculated as the number of counted reads per 1000 mappable and countable bases per geometric mean of normalized read counts per million. Genes that had an average total RNA RPKM > 5 were designated as robustly expressed.

Ribosome densities (alternatively known as translational efficiencies, TE) were then calculated as the ratio of footprint-RPKM to total RNA-RPKM for monosomes and disomes per sample. For most analysis downstream, densities were $\log_2$ transformed. Significant changes in abundances of total RNA, monosomes and disomes between control and treated samples were assessed by DESeq2 package for the R statistical environment (Team, 2013). Significant changes in densities of monosomes and disomes between control and treated samples were assessed by R package xtail. The false discovery rate (FDR) adjusted p values were used to identify statistically significant changes at 0.05 FDR.

Monosome and Disome Positions on Transcripts

For total RNA and RPF reads that were counted toward genes, we have also tracked the position of the reads relative to the 5′ end of its corresponding transcript. For total RNA reads and monosome footprints we have used the 5′ end of the reads and the estimated A-site of the ribosomes, respectively, as described in (Janich et al., 2015). For disome footprints we have established an empirical offsetting scheme based on the size and the frame (relative to the main CDS) of the footprints. We estimated the A-site of the pausing ribosome at the disome site by adding 45, 44, or 43 to the map position of the 5′ end of 58nt-long foot- prints that were at 1st, 2nd or 3rd frame, respectively. Similarly, we used the following offsets for 59nt, 60nt, 62nt and 63nt long disome footprints, respectively: [45, 44, 46], [45, 44, 46], [48, 47, 46], [48, 47, 49]. These coordinates then were converted into Wiggle Track Format (WIG) by in-house Python scripts.

Ribosome profiling versus CRAC for uORFs

uORFs were defined using our ribosome profiling data, as described in (Castelo-Szekely et al., 2019). Briefly, transcripts that are the only protein-coding isoform expressed were used (n = 7593), so that footprints can be unambiguously assigned to the 5′ UTR. uORFs were annotated based on the following criteria: 1) started with AUG, 2) had an in-frame stop codon within the 5′ UTR or within the CDS (overlapping uORFs) and 3) were at least 6 nt long (including the stop codon).

CRAC and ribosome profiling reads overlapping with uORFs and main CDSes were counted, and normalized to 100000 for each sample. To group uORFs by AVEN occupancy in WT cells, AVEN uORF CRAC was normalized to uORF ribosome profiling counts in WT cells, then uORFs classified by this value (low ≤ 5; medium > 5 and ≤ 20; high > 20). Differential uORF translation for *Aven*$^{-/-}$ versus WT cells was then calculated by normalizing uORF ribosome profiling counts to main CDS ribosome profiling counts for the two cell lines, then calculating a $\log_2$ fold change.

A similar approach was used to compare SKIV2L binding to uORFs in *Aven*$^{-/-}$ versus WT cells, whereby SKIV2L uORF CRAC counts were normalized to main CDS ribosome profiling counts (to account for changes in overall mRNA translation) before

calculating a $\log_2$ fold change for *Aven*$^{-/-}$ versus WT. Only uORFs with at least 20 counts per 100k for either SKIV2L in WT, or SKIV2L in *Aven*$^{-/-}$, were used. Additionally, the main CDS was required to have at least 2 counts per 100k for monosomes in both WT and *Aven*$^{-/-}$ conditions. The rationale here was to only include uORFs where uORF:CDS ratios could be calculated without being dominated by noise/background.

DATA AND CODE AVAILABILITY

The accession number for the sequencing data reported in this paper is GEO: GSE134020. R code and scripts used for analysis are available upon request. Original western blots were deposited in Mendeley Data and are available at https://data.mendeley.com/datasets/c6zdfw957p/1

Molecular Cell, Volume *77*

Supplemental Information

Mammalian RNA Decay Pathways Are Highly Specialized and Widely Linked to Translation

Alex Charles Tuck, Aneliya Rankova, Alaaddin Bulak Arpat, Luz Angelica Liechti, Daniel Hess, Vytautas Iesmantavicius, Violeta Castelo-Szekely, David Gatfield, and Marc Bühler

SUPPLEMENTAL FIGURE LEGENDS

Figure S1: Profiling mammalian RNA decay pathways (related to Figure 1). A Western blots showing expression and biotinylation of endogenously tagged 3xFLAG-AviTag SKIV2L, XRN1 and MTR4. **B** Scree plot for the PCA in Figure 1C. **C** Western blot analysis of a co-immunoprecipitation using anti-HA to capture 2xHA-FKBP12-tagged DIS3L, and probing inputs and eluates with anti-FLAG (to detect 3xFLAG-Avi-SKIV2L) and anti-MTR4. **D** tSNE representation of mRNAs based on relative binding to MTR4, SKIV2L and XRN1, and highlighting functional classes of mRNAs. **E** RT-qPCR analysis of *Skiv2l*$^{fl/fl}$ mRNA expression levels in clones 5F and 9E (Table S1) after 0.1 µM 4OHT treatment for 0, 2, 4 and 6 days. Error bars denote standard deviation of two technical replicates. Values are normalized to TBP expression and then to untreated samples (day 0). **F** Boxplot for the 7240 mRNAs analyzed in Figure 1I, grouped by differential expression after four days of *Skiv2l* knockout, and showing the extent to which they bind SKIV2L in WT cells (relative binding based on CRAC, defined in Figure 1E). Box widths are proportional to the number of contained transcripts. $p < 10^{-15}$ for upregulated versus downregulated transcripts (Mann-Whitney U test). **G** Log_2-fold changes in transcriptome-wide mRNA half-lives for *Skiv2l*$^{fl/fl}$ cells following 0.1 µM 4OHT treatment for 4 or 0 days. Half-lives were calculated by fitting an exponential decay model to RNA-seq counts from an actinomycin D-mediated transcription shut-off time course. The x-axis shows the extent of SKIV2L binding as a fraction of total SKIV2L+XRN1 binding (i.e. SKIV2L CRAC divided by SKIV2L+XRN1 CRAC, using "relative binding" values defined in Figure 1E). High confidence SKIV2L targets are indicated in red, and are defined as mRNAs with an increased half-life after *Skiv2l* knockout (y-axis > 0.1), high SKIV2L binding (x-axis > 0.5) and significant RNA increase after 4 days of *Skiv2l* knockout (Figure 1I). The half-lives of the 200 most highly SKIV2L-bound mRNAs (right of green dashed line) were compared to those of all other mRNAs using a Student's t-test. **H** RNA levels for high confidence SKIV2L targets (red circles in G) for wild-type, tagged (*Skiv2l*$^{3xFLAG\text{-}Avi/3xFLAG\text{-}Avi}$, *Mtr4*$^{3xFLAG\text{-}Avi/3xFLAG\text{-}Avi}$ and *Xrn1*$^{3xFLAG\text{-}Avi/3xFLAG\text{-}Avi}$) and *Skiv2l*$^{fl/fl}$ (+ 4 days 4OHT) cells. Each point refers to a separate cell line. See also Tables S1-S3.

Figure S2: Defining triggers of RNA decay (related to Figure 2). A Proportion of CRAC mRNA reads with non-templated homopolymeric 3' tails, for XRN1, SKIV2L and MTR4. **B** SKIV2L specific enrichment at amino acid pairs, compared to a panel of control datasets. For each amino acid pair, the proportion of hexamers encoding that amino acid pair and bound by SKIV2L is shown. This calculation was performed for in-frame hexamers (y-axis) and out-of-frame hexamers (x-axis). **C** Disome profiling (A sites) and SKIV2L and XRN1 CRAC coverage (3' end of RNA fragments) around runs of 3-4 identical amino acids (e.g. KKK/KKKK), normalized to downstream signal. **D** Pileups around polypurine tracts for monosome and disome profiling and SKIV2L, MTR4 and XRN1 CRAC signal. Polypurine tracts are divided by lysine content (top) or A versus G content ($\geq$ 50 % A, or > 50 % G).

Figure S3: AVEN modulates SKIV2L binding (related to Figure 4). A Scree plot for the PCA in Figure 4C. **B** Left, schematic of CRISPR-Cas9-induced *Aven* knockout and RNA-seq tracks in the parental *Skiv2l*$^{3xFLAG\text{-}Avi/3xFLAG\text{-}Avi}$ and *Aven*$^{-/-}$ clones 4H and 6G. Right, western blot analysis of AVEN expression. **C** RT-qPCR analysis of *Focad* mRNA levels in *Focad*$^{-/-}$ clones 2F and 4B and WT cells. Error bars denote standard deviation of four technical replicates. **D and E** Boxplot representation of the data shown in Figure 4F, including all transcripts (not only those differentially bound or expressed). AVEN CRAC counts in WT are shown (y-axis) for transcripts grouped by changes in SKIV2L binding (B; $p < 10^{-15}$) or RNA abundance (C; $p = 0.002$) in *Aven*$^{-/-}$ versus WT (x-axis). P-values calculated using a Mann-Whitney U test to compare up- and down-regulated transcripts. See also Tables S1 and S3.

Figure S4: AVEN and SKIV2L counteract ribosome stalling (related to Figures 4 and 5). A Changes in mRNA monosome and disome densities in *Aven*$^{-/-}$ versus WT. Transcripts are colored by AVEN binding in WT cells (calculated as in Figure 4F; high, n = 777; low, n = 5471), and a linear best fit line plotted for each group (shaded area = 95 % confidence interval). **B** Western blot analysis of SKIV2L levels in *Aven*$^{-/-}$

Skiv2l$^{3xFLAG\text{-}Avi/3xFLAG\text{-}Avi}$ *Skiv2l*$^{fl/fl}$ cells after treatment with 0.1 µM 4OHT for 0, 2 or 4 days to induce *Skiv2l* knockout. **C** Log$_2$ fold change in the half-lives of AVEN targets, which are defined as mRNAs that accumulate upon combined knockout of *Aven* and *Skiv2l* ($p < 0.01$; Figure 5D) and that have increased SKIV2L binding in *Aven*$^{-/-}$ cells (differential binding > 0.5, Figure 5D). Three comparisons are shown (left to right): (i) *Skiv2l*$^{fl/fl}$ 0 vs 4 days 4OHT (i.e. *Skiv2l* knockout vs wild-type), (ii) *Skiv2l*$^{fl/fl}$ vs *Aven*$^{-/-}$ *Skiv2l*$^{3xFLAG\text{-}Avi/3xFLAG\text{-}Avi}$ *Skiv2l*$^{fl/fl}$ with no 4OHT treatment (i.e. *Aven* knockout vs wild-type), and (iii) *Aven*$^{-/-}$ *Skiv2l*$^{3xFLAG\text{-}Avi/3xFLAG\text{-}Avi}$ *Skiv2l*$^{fl/fl}$ 0 vs 4 days 4OHT (i.e. *Skiv2l Aven* double knockout versus *Aven* knockout). Half-lives are calculated from RNA-seq decay curves following actinomycin D-mediated transcription shut-off. Replication-dependent histone mRNAs are colored red. For each of the three comparisons, a Student's t-test was used to test whether the distribution of half-life log$_2$-fold changes for the set of mRNAs shown here (AVEN targets) differs from that of all other mRNAs. **D** Top, scheme depicting mESC cell cycle synchronization at the G1/S boundary using a double-thymidine block. Bottom, flow cytometry analysis of DAPI-stained asynchronous or thymidine-synchronized cells at 0, 4 and 8 hours after release from the second thymidine block for the indicated cell lines. **E** Boxplot showing the change in uORF monosome profiling counts (normalized to main CDS counts) for *Aven*$^{-/-}$ versus WT cells. uORFs are categorized (x-axis) by their AVEN CRAC counts in WT cells (n = 558, 215 and 53 for low, medium and high categories; Mann-Whitney U test $p = 3.19 \times 10^{-6}$ comparing high and low categories). **E** Western blot analysis of ATF4 and eIF2α-phospho-Serine51 levels in WT and *Aven*$^{-/-}$ cells after 0, 0.5, 2 and 4 hours of treatment with 200 nM thapsigargin. See also Tables S3 and S4.

Figure S5: Defining sites dependent on AVEN and SKIV2L (related to Figure 6). **A** Log$_2$ fold changes in SKIV2L CRAC counts for 1 kb windows tiling the genome, in *Aven*$^{-/-}$ versus WT cells. Points are colored by the proportion of U-tailed reads within each window in WT (left) or *Aven*$^{-/-}$ (right) cells. **B** SKIV2L CRAC, disome and monosome profiling reads piled up around predicted structured regions (minimum free

energy <-12 kcal/mol and continuous stretch of paired nucleotides ≥10), normalized to the downstream region. For SKIV2L CRAC in *Aven*$^{-/-}$, U-tailed reads are also shown.

Figure S6: Features of translated non-coding RNAs (related to Figure 7). A GC content and **B** predicted free energy for the "non-coding" 1 kb genomic windows analyzed in Figure 7C. Windows are split into four categories based on differential SKIV2L CRAC counts in *Aven*$^{-/-}$ versus WT cells (defined in Figure 7C). **C** PhyloCSF scores for predicted small ORFs within these windows. Windows are classified based upon whether SKIV2L CRAC and monosome profiling counts increase in *Aven*$^{-/-}$ versus WT cells, or not. See also Tables S6 and S7.

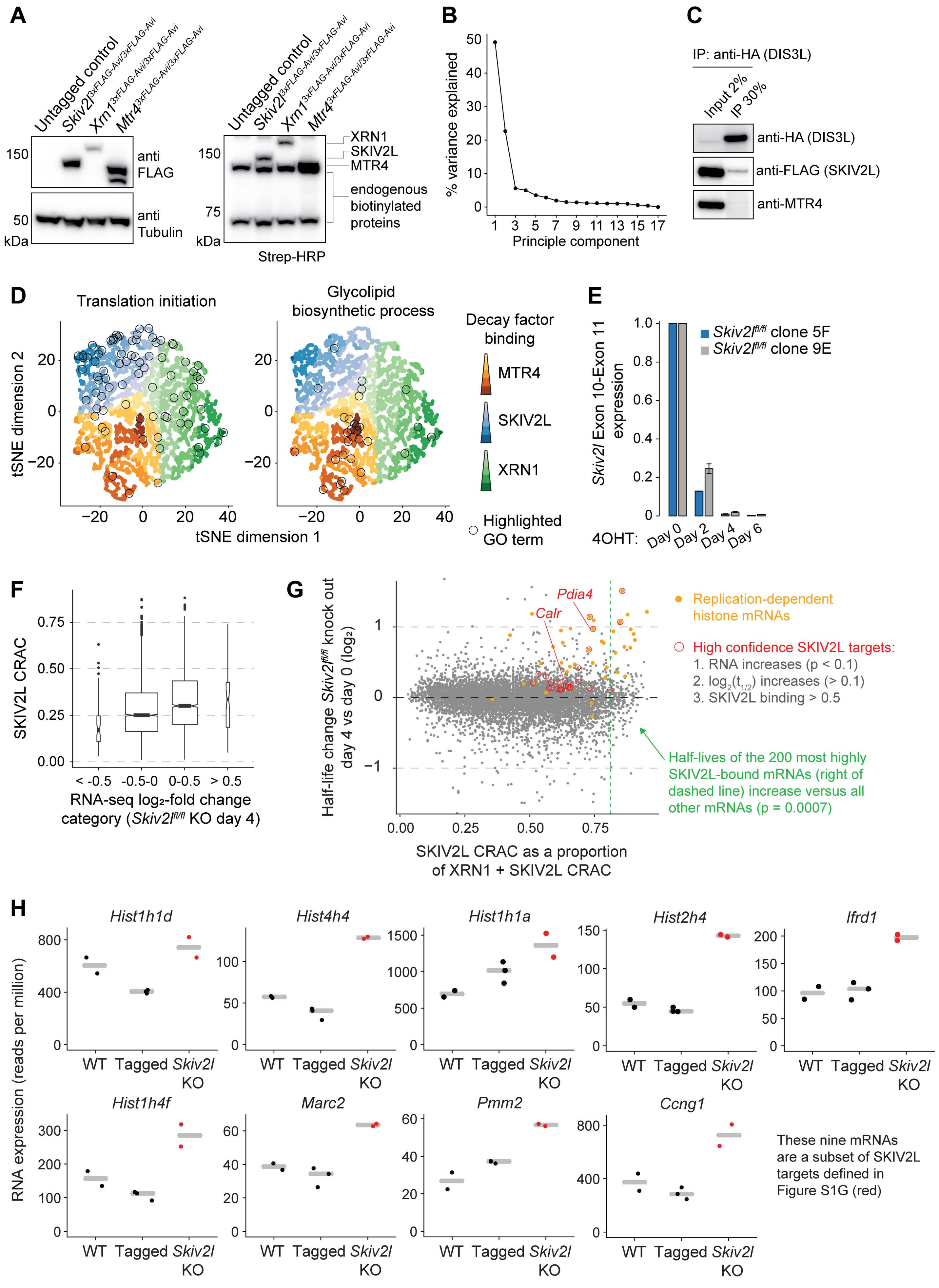

A
Untagged control
Skiv2l3xFLAG-Avi/3xFLAG-Avi
Xrn13xFLAG-Avi/3xFLAG-Avi
Mtr43xFLAG-Avi/3xFLAG-Avi
150
50
kDa
anti FLAG
anti Tubulin
75
XRN1
SKIV2L
MTR4
endogenous biotinylated proteins
Strep-HRP
B
% variance explained
Principle component
C
IP: anti-HA (DIS3L)
Input 2%
IP 30%
anti-HA (DIS3L)
anti-FLAG (SKIV2L)
anti-MTR4
D
Translation initiation
Glycolipid biosynthetic process
tSNE dimension 2
tSNE dimension 1
Decay factor binding
MTR4
SKIV2L
XRN1
Highlighted GO term
E
Skiv2l Exon 10-Exon 11 expression
Skiv2lfl/fl clone 5F
Skiv2lfl/fl clone 9E
4OHT: Day 0 Day 2 Day 4 Day 6
F
SKIV2L CRAC
< -0.5
-0.5-0
0-0.5
> 0.5
RNA-seq log2-fold change category (Skiv2lfl/fl KO day 4)
G
Half-life change Skiv2lfl/fl knock out day 4 vs day 0 (log2)
Pdia4
Calr
SKIV2L CRAC as a proportion of XRN1 + SKIV2L CRAC
Replication-dependent histone mRNAs
High confidence SKIV2L targets:
1. RNA increases (p < 0.1)
2. log2(t1/2) increases (> 0.1)
3. SKIV2L binding > 0.5
Half-lives of the 200 most highly SKIV2L-bound mRNAs (right of dashed line) increase versus all other mRNAs (p = 0.0007)
H
RNA expression (reads per million)
Hist1h1d
Hist4h4
Hist1h1a
Hist2h4
Ifrd1
Hist1h4f
Marc2
Pmm2
Ccng1
WT
Tagged
Skiv2l KO
These nine mRNAs are a subset of SKIV2L targets defined in Figure S1G (red)

Figure S1

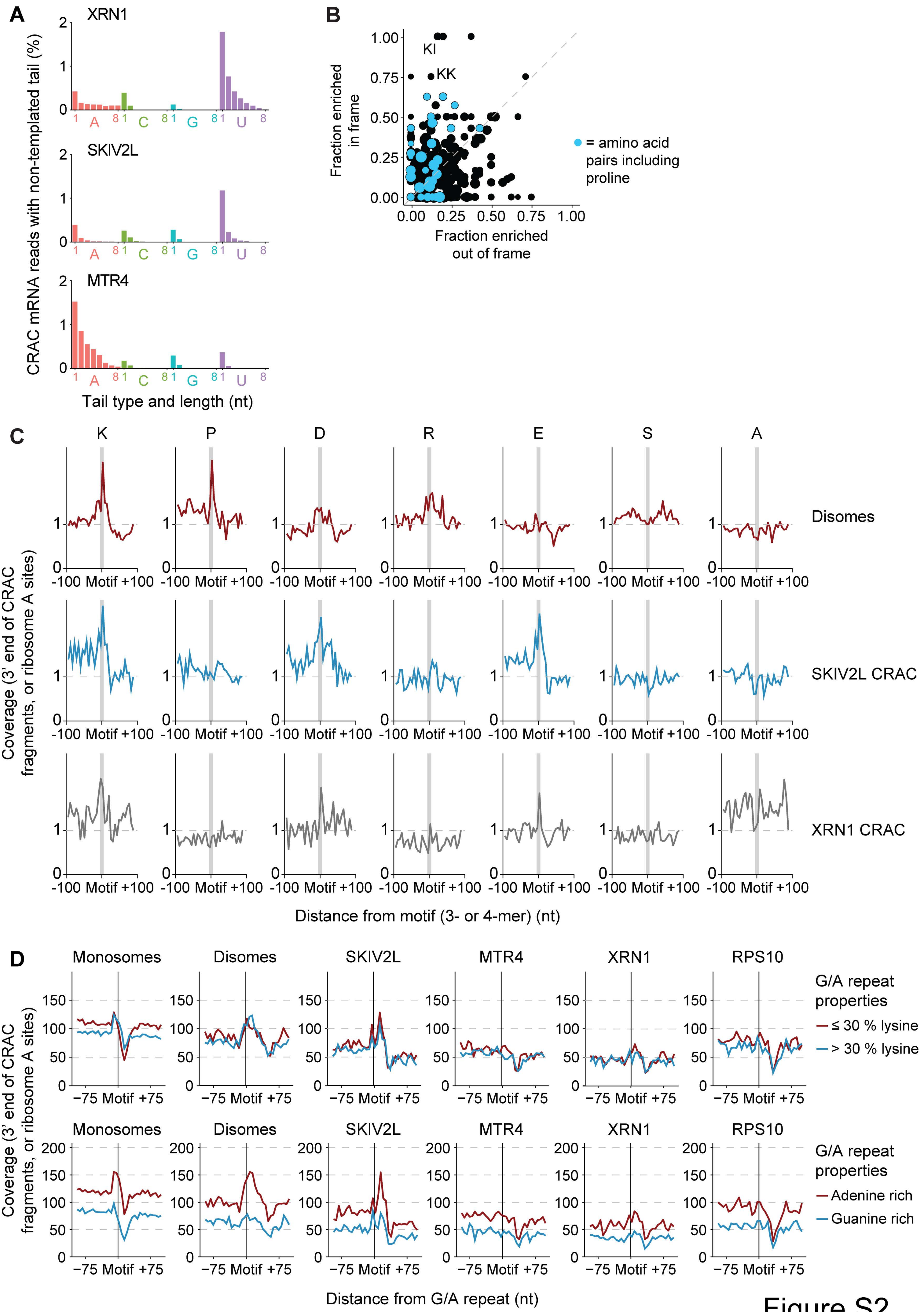
A
XRN1
SKIV2L
MTR4
CRAC mRNA reads with non-templated tail (%)
Tail type and length (nt)
A
C
G
U
B
KI
KK
Fraction enriched in frame
Fraction enriched out of frame
= amino acid pairs including proline
C
K
P
D
R
E
S
A
Disomes
SKIV2L CRAC
XRN1 CRAC
-100 Motif +100
Coverage (3' end of CRAC fragments, or ribosome A sites)
Distance from motif (3- or 4-mer) (nt)
D
Monosomes
Disomes
SKIV2L
MTR4
XRN1
RPS10
−75 Motif +75
G/A repeat properties
≤ 30 % lysine
> 30 % lysine
Adenine rich
Guanine rich
Coverage (3' end of CRAC fragments, or ribosome A sites)
Distance from G/A repeat (nt)

Figure S2

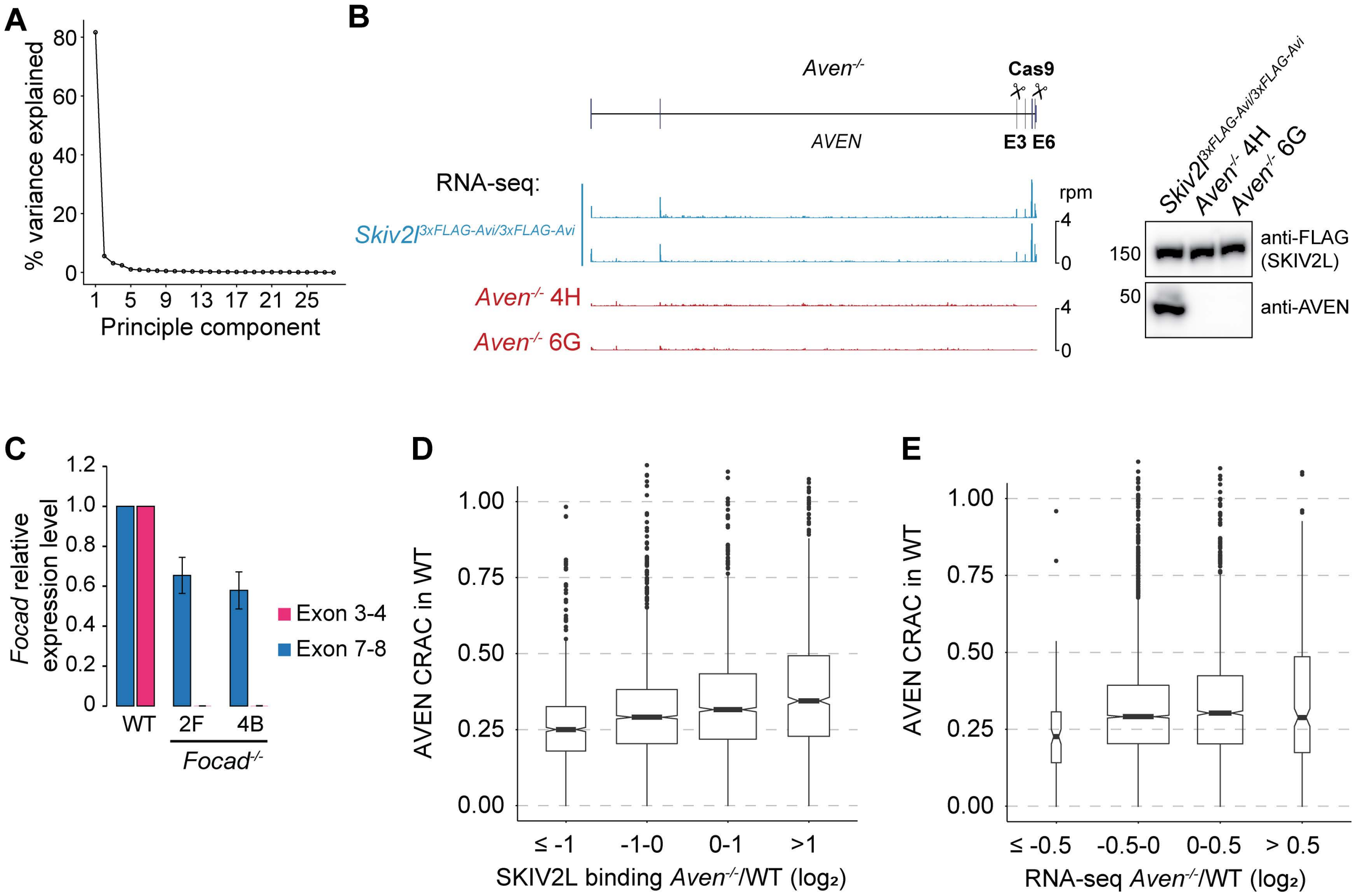
A
% variance explained
Principle component
B
Aven-/-
Cas9
AVEN
E3 E6
RNA-seq:
Skiv2l3xFLAG-Avi/3xFLAG-Avi
Aven-/- 4H
Aven-/- 6G
rpm
Skiv2l3xFLAG-Avi/3xFLAG-Avi
Aven-/- 4H
Aven-/- 6G
150
50
anti-FLAG
(SKIV2L)
anti-AVEN
C
Focad relative expression level
Exon 3-4
Exon 7-8
WT
2F
4B
Focad-/-
D
AVEN CRAC in WT
SKIV2L binding Aven-/-/WT (log2)
≤ -1
-1-0
0-1
>1
E
AVEN CRAC in WT
RNA-seq Aven-/-/WT (log2)
≤ -0.5
-0.5-0
0-0.5
> 0.5

Figure S3

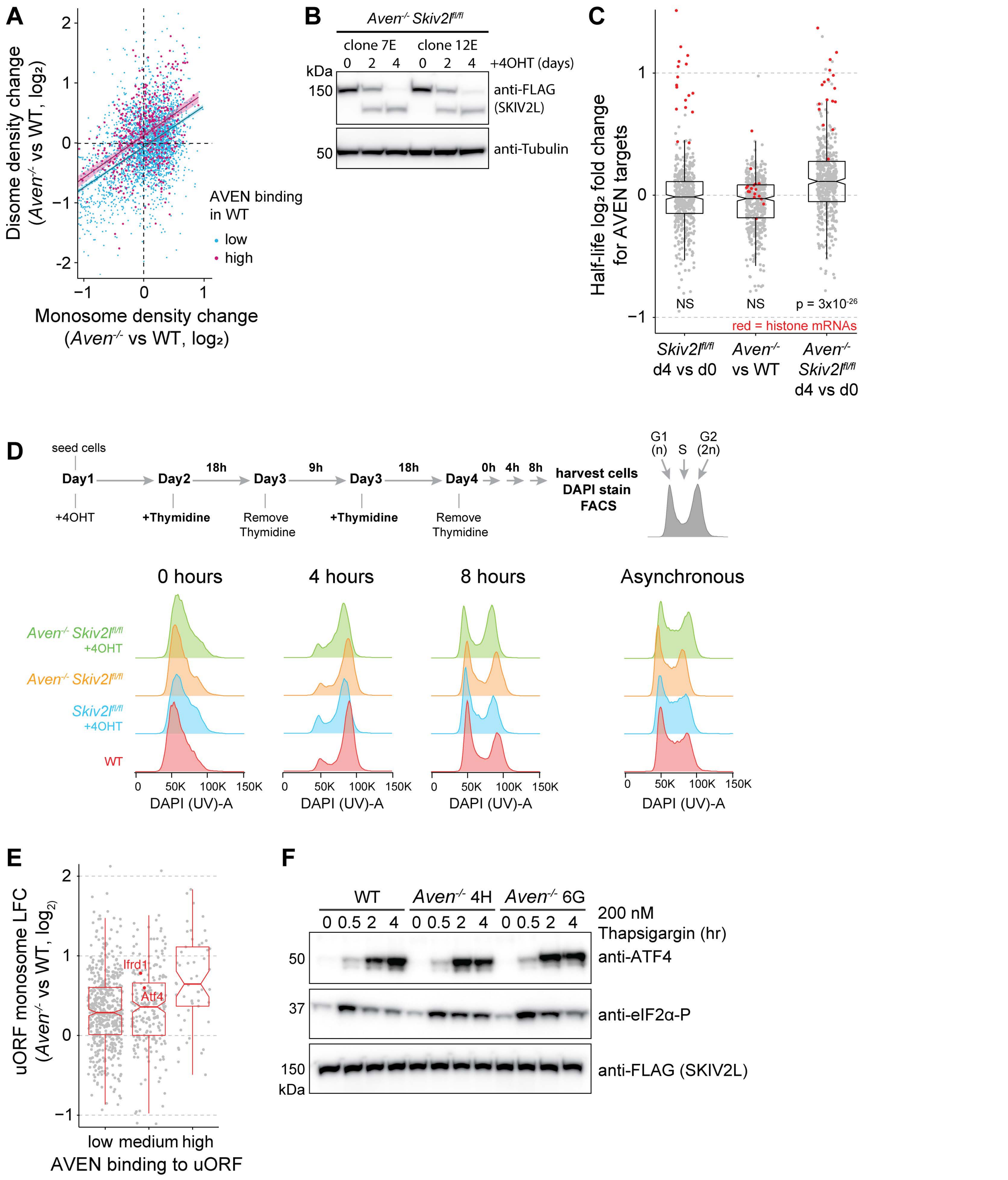

A
Disome density change (Aven-/- vs WT, log2)
Monosome density change (Aven-/- vs WT, log2)
AVEN binding in WT
low
high
B
Aven-/- Skiv2lfl/fl
clone 7E
clone 12E
+4OHT (days)
kDa
150
50
anti-FLAG (SKIV2L)
anti-Tubulin
C
Half-life log2 fold change for AVEN targets
NS
NS
p = 3x10-26
red = histone mRNAs
Skiv2lfl/fl d4 vs d0
Aven-/- vs WT
Aven-/- Skiv2lfl/fl d4 vs d0
D
seed cells
Day1
Day2
Day3
Day3
Day4
18h
9h
18h
0h
4h
8h
+4OHT
+Thymidine
Remove Thymidine
+Thymidine
Remove Thymidine
harvest cells DAPI stain FACS
G1 (n)
S
G2 (2n)
0 hours
4 hours
8 hours
Asynchronous
Aven-/- Skiv2lfl/fl +4OHT
Aven-/- Skiv2lfl/fl
Skiv2lfl/fl +4OHT
WT
DAPI (UV)-A
E
uORF monosome LFC (Aven-/- vs WT, log2)
Ifrd1
Atf4
low medium high
AVEN binding to uORF
F
WT
Aven-/- 4H
Aven-/- 6G
200 nM Thapsigargin (hr)
anti-ATF4
anti-eIF2α-P
anti-FLAG (SKIV2L)
kDa

Figure S4

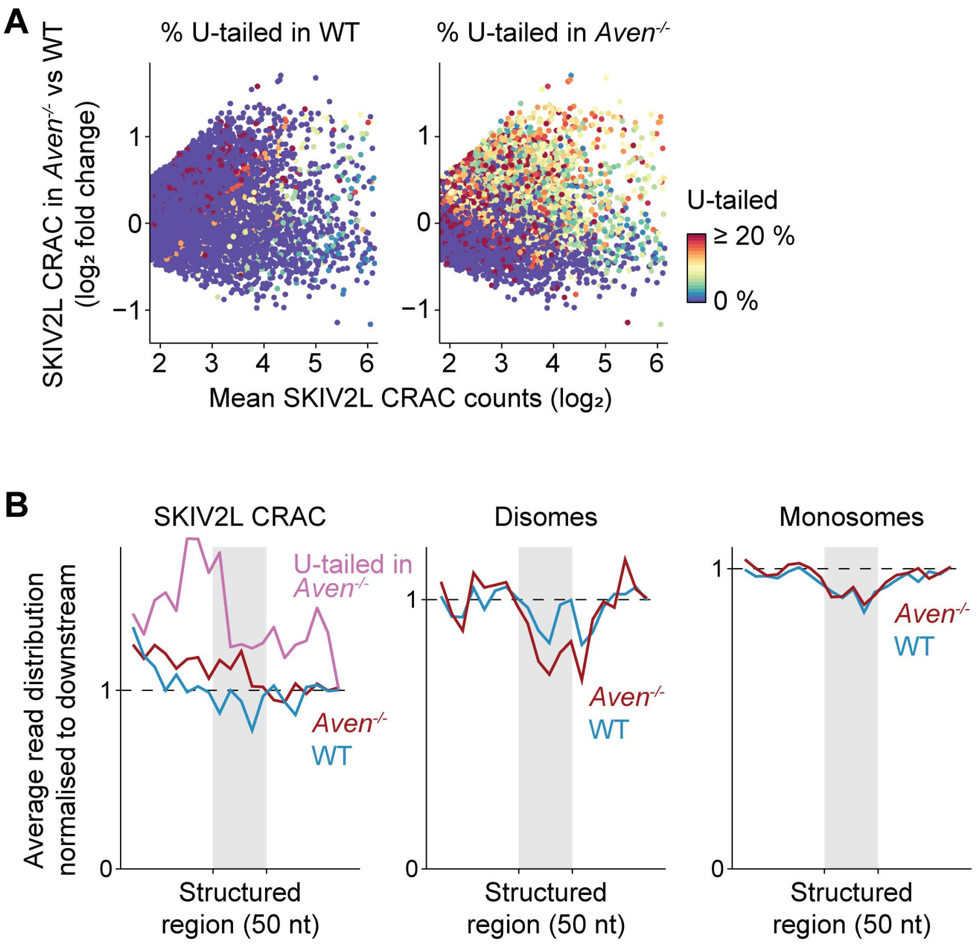

A
% U-tailed in WT
% U-tailed in Aven-/-
SKIV2L CRAC in Aven-/- vs WT (log₂ fold change)
Mean SKIV2L CRAC counts (log₂)
U-tailed
≥ 20 %
0 %
B
SKIV2L CRAC
Disomes
Monosomes
Average read distribution normalised to downstream
U-tailed in Aven-/-
Aven-/-
WT
Structured region (50 nt)

Figure S5

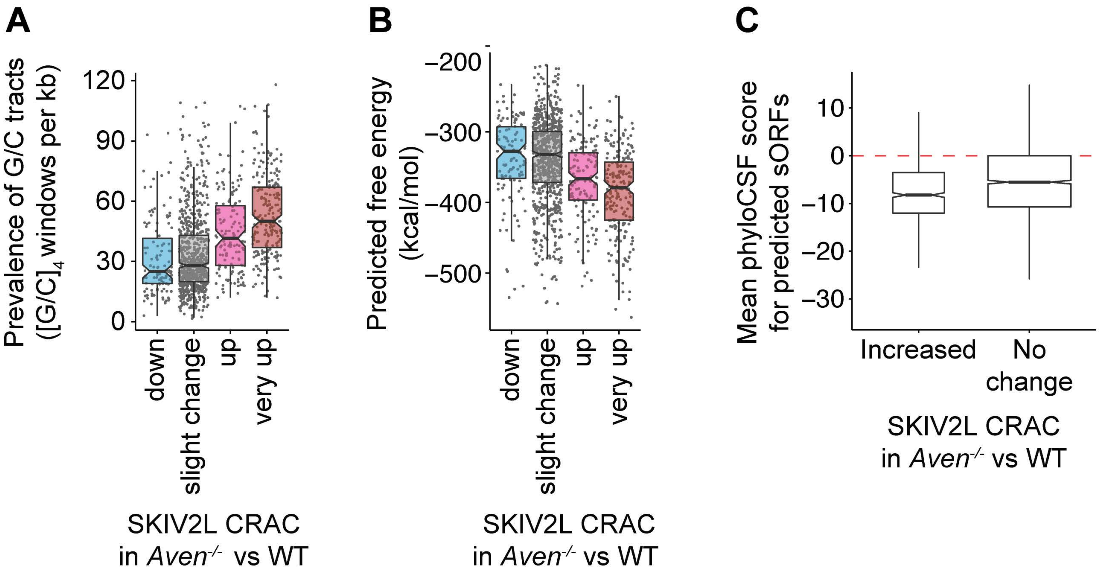
A
Prevalence of G/C tracts
([G/C]4 windows per kb)
120
90
60
30
0
down
slight change
up
very up
SKIV2L CRAC
in Aven-/- vs WT
B
Predicted free energy
(kcal/mol)
−200
−300
−400
−500
down
slight change
up
very up
SKIV2L CRAC
in Aven-/- vs WT
C
Mean phyloCSF score
for predicted sORFs
10
0
−10
−20
−30
Increased
No
change
SKIV2L CRAC
in Aven-/- vs WT

Figure S6
